# Nonparametric bounds in two‐sample summary‐data Mendelian randomization: Some cautionary tales for practice

**DOI:** 10.1002/sim.9368

**Published:** 2022-03-30

**Authors:** Ralph Møller Trane, Hyunseung Kang

**Affiliations:** ^1^ Department of Statistics University of Wisconsin–Madison Madison Wisconsin USA

**Keywords:** causal inference, instrument strength, Mendelian randomization, nonparametric bounds, two‐sample studies

## Abstract

Recently, in genetic epidemiology, Mendelian randomization (MR) has become a popular approach to estimate causal exposure effects by using single nucleotide polymorphisms from genome‐wide association studies (GWAS) as instruments. The most popular type of MR study, a two‐sample summary‐data MR study, relies on having summary statistics from two independent GWAS and using parametric methods for estimation. However, little is understood about using a nonparametric bound‐based analysis, a popular approach in traditional instrumental variables frameworks, to study causal effects in two‐sample MR. In this article, we explore using a nonparametric, bound‐based analysis in two‐sample MR studies, focusing primarily on implications for practice. We also propose a framework to assess how likely one can obtain more informative bounds if we used a different MR design, notably a one‐sample MR design. We conclude by demonstrating our findings through two real data analyses concerning the causal effect of smoking on lung cancer and the causal effect of high cholesterol on heart attacks. Overall, our results suggest that while a bound‐based analysis may be appealing due to its nonparametric nature, it is far more conservative in two‐sample settings than in one‐sample settings to get informative bounds on the causal exposure effect.

## 1 INTRODUCTION

In recent years, genetic variants, often in the form of single nucleotide polymorphisms (SNPs), have been used as instrumental variables (IV) to estimate causal effects in epidemiological studies, often referred to as Mendelian randomization (MR) studies.^1^, ^2^, ^3^ Typically, MR studies are based on a two‐sample design where published summary statistics from two independent genome wide association studies (GWAS), with one providing information about the exposure and the other about the outcome, are used.^4^, ^5^, ^6^ Under a two‐sample design, investigators frequently use parametric methods to study exposure effects, for instance the IVW estimator,^4^ MR‐Egger regression,^7^ the weighted median estimator,^8^ MR‐PRESSO^9^ and MR‐RAPS;^10^ see References 3, 11, 12 for recent reviews.

An alternative approach to study exposure effects using instrumental variables without parametric assumptions is through nonparametric IV bounds.^13^, ^14^, ^15^, ^16^, ^17^ Briefly, nonparametric IV bounds use a minimum set of assumptions to provide a range of plausible values for the exposure effect. They are typically used when the outcome, the exposure, and the instrument are all binary and are simultaneously observed; we refer to this setting as the one‐sample setting to contrast it from the two‐sample setting. Arguably, the most well‐known IV bound is the Balke‐Pearl bound^13^ for the average treatment effect. The Balke‐Pearl bound has been extended to allow for a nonbinary instrument^14^, ^16^ and two‐sample designs;^18^ see Reference 19 and references therein for a recent summary of IV bounds.

Using IV bounds can be an attractive alternative to study exposure effects in MR studies given the strong parametric assumptions accompanying most MR analyses.^20^, ^21^ However, to the best of our knowledge and compared to parametric methods, there is little work on actually using these bounds in typical MR settings, that is, two‐sample designs with summary statistics, nor any practical guidance on when the bounds would be informative. For example, what kind of genetic variants provide the most informative conclusions about the exposure effect in terms of the bounds not containing the null effect? Can combining multiple variants lead to shorter and tighter bounds? How do the bounds change if many instruments are weak, which is typical in MR studies? The overall goal of this article is to offer some practical guidance on using IV bounds in two‐sample MR studies. We focus on two aspects of bounds that will better inform MR investigators about the exposure effect: (1) the length of the bounds and (2) whether the bounds cover the null effect of zero (ie, direction/sign of the effect).

The article is organized as follows. Section 2 reviews notation, definition, and methods for studying the exposure effect with parametric models and nonparametric bounds. Section 3 presents four results where we show the behavior of the bounds in two‐sample settings when we have one or multiple instruments, and when some of the core assumptions are violated. Section 4 quantifies how bounds from two‐sample data is more conservative than bounds from one‐sample data. Section 5 presents the data analysis and Section 6 lays out some concrete practical recommendations for using bounds in two‐sample MR studies.

## 2 METHODS

### 2.1 Review: Notation, definitions, and assumptions

Let *X* and *Y* be binary exposure and outcome variables, respectively, *Z* be a categorical instrumental variable taking values in {0,1,2}, and *U* be an unmeasured confounder for the effect of *X* on *Y*. We consider trivariate instruments due to the trivariate nature of SNPs that are typically used as instruments in MR studies. Also, following prior literature on bounds,^13^, ^15^, ^16^, ^19^ we consider the binary exposure and binary outcome setting. In Section 3.4 and Appendix H, we discuss how to use the bounds to detect the sign of the effect when the exposure is continuous and the outcome is binary under some an additional assumptions. In general, nontrivial bounds (eg, bounds that do not cover the entire support of the outcome) under “standard” IV assumptions stated below are impossible when the exposure or outcome is continuous. This limitation is well‐known for bound‐based analyses of causal effects and investigators who wish to obtain an effect estimate with a continuous exposure or outcome may have to make untestable, parametric modeling assumptions on top of the standard IV assumptions; see Swanson^19^ and Burgess and Labrecque^22^ for further discussions.

Let $Y^{z,x}$ be the potential outcome^23^, ^24^ had the subject received exposure value X=x and instrument value Z=z. We assume the stable unit treatment value assumption (SUTVA),^25^, ^26^ formalized as $Y=\sum_{x,z}I[Z=z,X=x]Y^{x,z}$ where I[·] is the indicator function.

We make the following set of assumptions about X,Y,Z, and *U* that are found in MR studies:^20^, ^27^

(A1) *(Relevance)*: Z⊥̸X
(A2) *(Independent instrument)*: Z⊥U
(A3) *(Exclusion restriction)*: $Y^{z,x}=Y^{z^{\prime },x}=Y^{x}$ for all $x,z,z^{\prime }$
(A4) (*Conditional ignorability of* X,Z *given U*): $Y^{z,x}⊥Z,X|U$

Briefly, (A1) can be satisfied by finding a SNP that has been consistently associated with the exposure. (A2) and (A3) are justified by scientific theory and can be violated if the SNP (i) is in linkage disequilibrium with an unmeasured SNP that affects the exposure and the outcome or (ii) has multiple functions beyond affecting the exposure (ie, pleiotropic), to name a few. Pleiotropy is often a great concern in MR studies; we will consider violations of (A3) in Section 3.3. Finally, (A4) states that if *U* is observed, then it is sufficient to unconfound the relationship between *X* and *Y*. For much of the article, we will assume (A1)‐(A4) hold to focus on the behavior of the bounds, even though these assumptions are important to assess in practice.

We make some additional remarks about assumptions (A1)‐(A4). First, in practice, most MR studies only explicitly state assumptions (A1)‐(A3) along with some parametric modeling assumptions;^3^ see Section 2.2 below. Second, one can remove (A4) and strengthen (A2) with $Z⊥U,Y^{z,x}$ without consequence on the IV bounds.^16^ Third, under SUTVA and assumptions (A3)‐(A4), we have Y⊥Z|X,U, which is another common way to express the exclusion restriction in MR studies.^19^, ^20^ Fourth, for simplicity, we do not assume the existence of a potential treatment $X^{z}$.

We define instrument strength ST as

(1) $$\text{ST}=\underset{z\neq z^{\prime }}{\max}|P(X=1|Z=z)-P(X=1|Z=z^{\prime })|.$$

ST reduces to the definition of instrument strength in Balke and Pearl's bounds when the instrument is binary. ST plays a critical role in determining the length of Balke and Pearl's IV bounds.^13^ Also, (1) differs from other definitions of instrument strength based on a parametric model between the exposure and the outcome, such as the concentration parameter $\mu^{2}$;^28^ the concentration parameter is roughly proportional to the observed first‐stage F‐statistic commonly used in linear IV models to assess instrument strength. But, under some assumptions, notably that the instrument is fixed, $\mu^{2}$ and ST are related by the following formula.

$$\mu^{2}=\frac{{\text{ST}}^{2}}{4}\overset{n}{\underset{i=1}{\sum }}z_{i}^{2}/\sigma^{2}.$$

Here $z_{1},\ldots ,z_{n}$ are observed values of the instrument, and $\sigma^{2}$ is the variance of the errors in the linear, first‐stage reduced model; see Appendix A for more details. The important take‐away from the formula is that stronger instruments as measured by ST lead to larger values of the concentration parameter $\mu^{2}$.

### 2.2 Review: Parametric models in two‐sample studies

To better contrast the bound‐based approaches we discuss below, we briefly review parametric models used to estimate exposure effects. Formally, in two‐sample MR studies, a popular parametric model for a binary exposure^2^, ^29^, ^30^, ^31^ is

(2) $$\text{logit}(P(X=1|Z_{1}=z_{1},\ldots ,Z_{p}=z_{p},U=u))=\gamma_{0}+\underset{i}{\sum }\gamma_{i}z_{i}+\gamma_{U}u,$$

and for a binary outcome^29^, ^30^ is

(3) $$\text{logit}(P(Y=1|X=x,U=u))=\beta_{0}+\beta_{X}x+\beta_{U}u,$$

where logit(a)=log(a/(1−a)). The parameter $\gamma_{i}$ corresponds to the effect that instrument *i* has on the exposure. The summary statistic reported in GWAS is the coefficient from a simple logistic regression model, that is, the model above where p=1. This summary statistic is also approximately equal to the coefficient $\gamma_{i}$ in Equation (2) if the instruments are independent of each other and the coefficients $\gamma_{1},\ldots ,\gamma_{p}$ are small, which is the case in most two‐sample MR studies.^32^ The parameter $\beta_{X}$ corresponds to the effect that the exposure has on the outcome in the logit scale; one can compute a numerical integral to compute the effect of the exposure on the outcome in the risk difference scale; see Section 2.3. The parameters $\gamma_{U}$ and $\beta_{U}$ correspond to the magnitudes that the unmeasured confounder *U* has on the exposure and outcome, respectively. Typically in the analysis of two‐sample MR studies, *U* follows a parametric distribution and each SNP is often assumed to be in Hardy‐Weinberg equilibrium. In our exposition below, we will relate the analysis from nonparametric bounds to these parametric models.

### 2.3 IV bounds under two‐sample designs and goals of article

The most popular design in MR studies is a two‐sample design which has two separate data sources, one providing information about (X,Z) in the form of P(X=1|Z=z), z∈{0,1,2}, and another providing information about (Y,Z) in the form of P(Y=1|Z=z), z∈{0,1,2}. A two‐sample design differs from a more traditional one‐sample design which has a single data source providing information on all observed variables (X,Y,Z) in the form of P(Y=y,X=x|Z=z) or P(X=x,Y=y,Z=z). IV bounds have been well‐studied in one‐sample designs and there is a rich array of guidance on how to use them in practice.^13^, ^16^, ^19^ However, as noted in the introduction, not much is known about the behavior of IV bounds under a two‐sample design.

The goal of this article is to offer useful practical advice on using IV bounds to study the average treatment effect (ATE), defined as

$$\text{ATE}=E[Y^{1}-Y^{0}]=\int P(Y=1|X=1,U=u)P(U=u)du-\int P(Y=1|X=0,U=u)P(U=u)du$$

using P(Y=1|Z=z) and P(X=1|Z=z), z∈{0,1,2} obtained from a two‐sample design. Specifically, under a two‐sample design and assumptions (A1)‐(A4),^18^ derived the following sharp bounds for the ATE:

(4) $$\begin{array}{ll} \max\left\{\begin{array}{ll} \underset{z\neq z^{\prime }}{\max} & P(Y=1|Z=z)-2\cdot P(Y=1|Z=z^{\prime })-2\cdot P(X=1|Z=z^{\prime })\\ \underset{z\neq z^{\prime }}{\max} & P(Y=1|Z=z)+P(X=1|Z=z)-P(Y=1|Z=z^{\prime })-P(X=1|Z=z^{\prime })-1\\ \underset{z\neq z^{\prime }}{\max} & 2\cdot P(Y=1|Z=z)+2\cdot P(X=1|Z=z)-P(Y=1|Z=z^{\prime })-3\\ \underset{z}{\max} & -P(Y=1|Z=z)-P(X=1|Z=z)\\ \underset{z}{\max} & P(Y=1|Z=z)+P(X=1|Z=z)-2 \end{array}\right\} & \\ \leq ATE\leq & \\ \min\left\{\begin{array}{ll} \underset{z\neq z^{\prime }}{\min} & P(Y=1|Z=z)-2\cdot P(Y=1|Z=z^{\prime })+2\cdot P(X=1|Z=z^{\prime })+1\\ \underset{z\neq z^{\prime }}{\min} & P(Y=1|Z=z)+2\cdot P(Y=1|Z=z^{\prime })-2\cdot P(X=1|Z=z^{\prime })+1\\ \underset{z\neq z^{\prime }}{\min} & P(Y=1|Z=z)-P(X=1|Z=z)+P(X=1|Z=z^{\prime })-P(Y=1|Z=z^{\prime })+1\\ \underset{z}{\min} & P(X=1|Z=z)-P(Y=1|Z=z)+1\\ \underset{z}{\min} & P(Y=1|Z=z)-P(X=1|Z=z)+1 \end{array}\right\}. & \end{array}$$

Furthermore, the assumptions imply the following checkable constraints, which are also referred to as IV inequalities,^13^, ^33^ on the observed data.

(5) $$\min\left\{\begin{array}{ll} \underset{z\neq z^{\prime }}{\min} & P(Y=1|Z=z)-P(X=1|Z=z)-P(Y=1|Z=z^{\prime })-P(X=1|Z=z^{\prime })+2\\ \underset{z\neq z^{\prime }}{\min} & P(Y=1|Z=z)+P(X=1|Z=z)-P(Y=1|Z=z^{\prime })+P(X=1|Z=z^{\prime })\\ \underset{z}{\min} & P(X=1|Z=z)\\ \underset{z}{\min} & P(Y=1|Z=z)\\ \underset{z}{\min} & 1-P(X=1|Z=z)\\ \underset{z}{\min} & 1-P(Y=1|Z=z) \end{array}\right\}\geq 0.$$

In Equation (5), we see that the constraints from the law of probability are recovered (the last four expressions in Equation (5)) along with 12 nontrivial constraints (the first two lines in Equation (5)). Appendix B provides additional discussion on Equations (4) and (5) in two‐sample MR studies.

We study two properties of the above bounds that can better guide practice: (1) the length of the bounds and (2) whether the bounds cover the null effect of zero. To better understand bound‐specific characteristics not due to sampling errors, we will assume we have population‐level quantities of P(Y=1|Z=z) and P(X=1|Z=z). In practice, these are estimated summary GWAS statistics from marginal logistic models. Specifically, the marginal proportions of the outcome, exposure, and allele frequencies are used to find the intercepts inside a logistic regression model by solving $P(X=1)=\sum_{z=0}^{2}\text{expit}(\gamma_{0}+{\hat{\gamma }}_{j}\cdot z)P(Z_{j}=z)$ and $P(Y=1)=\sum_{z=0}^{2}\text{expit}(\Gamma_{0}+{\hat{\Gamma }}_{j}\cdot z)P(Z_{j}=z)$ for $\gamma_{0}$ and $\Gamma_{0}$, respectively; here, ${\hat{\gamma }}_{j}$ and ${\hat{\Gamma }}_{j}$ are the estimated log odds ratio of the (marginal) associations from GWAS, and expit is the inverse of the logit function. This allows us to obtain estimates of $P(Y=1|Z_{j}=z)$ and $P(X=1|Z_{j}=z)$ for every *j* and z=0,1,2.

Finally, we remark that the population‐level bounds do not depend on P(Z=z),z=0,1,2. In particular, whether a variant is rare or common has no influence on the bounds. However, rare variants may make it difficult to estimate the conditional probabilities which make up these bounds. Since we are only examining population‐level characteristics of the bounds, we will assume $P(Z_{i}=0)=P(Z_{i}=2)=0.25$ and $P(Z_{i}=1)=0.5$ when we numerically illustrate our results below.

## 3 PROPERTIES OF IV BOUNDS

### 3.1 Length of bounds and coverage of null effect

Theorem 1 characterizes the length of the IV bound in Equation (4) under a two‐sample design and assumptions (A1)‐(A4).

Theorem 1 *Under assumptions (A1)‐(A4) and a two‐sample design, a sharp upper bound on the length of the bound in Equation (* *4* *) is* 2−2·ST *, that is, there exists a data generating process satisfying (A1)‐(A4) and has length equal to* 2−2·ST.

See Appendix C for the proof, which extends Theorem 1 to instruments with 2, 3, or 4 categories. Compared to the Balke‐Pearl IV bounds under a one‐sample design where the length is 1−ST for a binary or three‐leveled IV,^13^, ^16^ the length of two‐sample IV bounds can be twice as long. Also, the length of two‐sample IV bounds is only guaranteed to be less than 1 if the instrument strength ST is greater than 0.5; note that this does not imply that instruments with ST less than 0.5 has length greater than 1. In contrast, one‐sample IV bounds always have length less than 1 unless ST is zero. In short, there is a cost of using a two‐sample MR design instead of a one‐sample MR design when performing a bound‐based analysis of the ATE.

**FIGURE 1:**
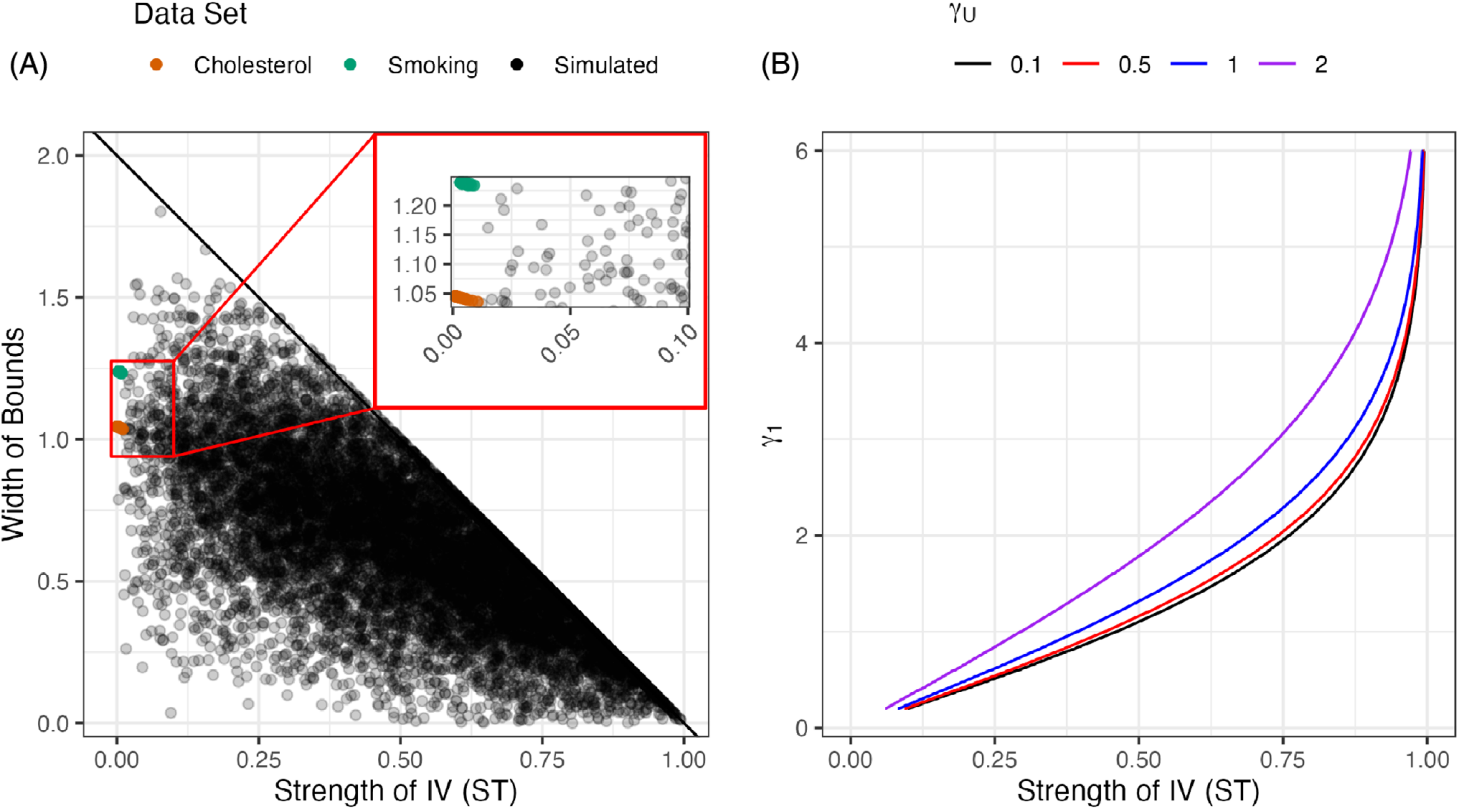
Illustration of the relationship between instrument strength, length of bounds, and coefficients from logistic regression model in two‐sample MR settings. (A) Relationship between instrument strength (ST) and length of the IV bounds. Black line is the upper bound on the two‐sample IV bounds from Theorem 1. Black dots indicate one of the 10,000 IV bounds. Colored dots indicate bounds from real data; see Section 5 for details. (B) Coefficients from logistic regression model and instrument strength (ST). Each color represents different magnitudes of unmeasured confounding

Figure 1A numerically illustrates the consequences of Theorem 1 by calculating the bounds in Equation (4) from 10,000 randomly generated sets of values of P(X=1|Z=z) and P(Y=1|Z=z) that satisfy the IV inequalities and assumptions (A1)‐(A4). We also use two real‐world data examples where the causal effects are known to exist: the effect of high cholesterol on incidence of heart attacks,^34^ and the effect of smoking on incidence of lung cancer.^35^ We see that the length of the bounds often exceed 1 as the instrument strength decreases. Also, the two real‐world studies generally do not lead to bounds with length less than 1.

Figure 1B further illustrates this point by characterizing the relationship between ST and the summary statistic coefficient $\gamma_{1}$ from the logistic exposure model in Section 2.2 when p=1 and *U* following a standard Normal. Specifically, $\gamma_{U}$ was set to 0.1,0.5,1 and 2, $\gamma_{1}$ varied between 0.2 and 6, and $\gamma_{0}=-\gamma_{1}$. We see that instrument strength ST of 0.5 corresponds to a coefficient $\gamma_{1}$ of approximately 1.1,1.16,1.4, and 1.8 if $\gamma_{U}$ is 0.1,0.5,1, and 2, respectively. Coefficients with such magnitudes are rare in GWAS where genetic variants often explain a small amount of variation in the exposure. More broadly, these values of $\gamma_{1}$ correspond to odds ratios between 3 and 6 and exceed some well‐known magnitudes of causal effects in cancer studies, say the effect of exposure to ultraviolet radiation on the incidence of skin cancer where the odds ratios are estimated to be in the range from 1.4 to 2.22.^36^

**FIGURE 2:**
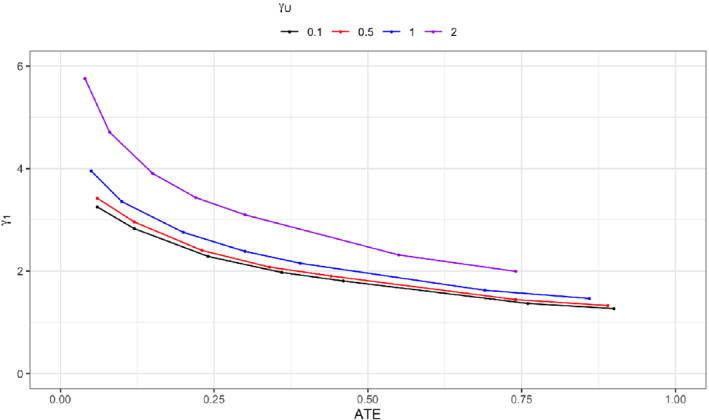
Relationship between the smallest $\gamma_{1}$ needed for a two‐sample IV bound to exclude 0 and the average treatment effect (ATE). Each color corresponds to different levels of unmeasured confounding

Next, we examine what kind of $\gamma_{1}$ is needed in order for the two‐sample IV bounds to exclude the null effect of zero for a specified effect size of the ATE. This question is akin to computing the power of bounds but with population‐level quantities. We reuse the setup for the exposure model described above and the logistic outcome model specified in Section 2.2. Specifically, the coefficients for the exposure model are the same as before. For the outcome model, we vary $\beta_{X}$ from 0.25 to 6 and set $\beta_{0}=-\beta_{X}/2$. Then, for each parameter specification in the outcome model, we compute the corresponding ATE. Afterwards, we find the smallest $\gamma_{1}$ that leads to a bound that does not cover 0, but covers the ATE; see Appendix D for more details. Figure 2 shows that even for a moderate effect size of 0.4, the corresponding $\gamma_{1}$ must be around 2, a tall order for most GWAS. Also, as the effect of unmeasured confounding increases via $\gamma_{U}$, a larger $\gamma_{1}$ is needed to exclude the null effect. In short, analyzing the ATE using bounds in a two‐sample MR study is unlikely to be informative; the bounds will often have length greater than 1 and rarely exclude the null effect unless very strong genetic variants are used.

### 3.2 Would multiple instruments help?

Based on the results above with a single instrument, a natural question from investigators is whether using multiple instruments can lead to more informative bounds for the ATE.^21^ For example, suppose we aggregate two‐sample IV bounds across multiple instruments by taking intersections of individual IV bounds; we refer to this as intersection bounds. This approach may be inferior to another alternative where we expand the levels of *Z* to accommodate multiple instruments,^21^ but has the benefit of being applicable to most two‐sample MR studies with summary statistics from GWAS. In particular, with typical summary‐level data from two‐sample MR studies where only marginal associations are reported, it is not possible to expand the levels of Z to accommodate multiple instruments. As we show here, the strongest instrument often determines the length of the intersection bound because the bounds from each instrument exhibit a nesting property. That is, using one bound with the strongest instrument often provides the same amount of information about the ATE as the intersection of several bounds from multiple instruments. We will illustrate this numerically in this section; Appendix E provides the exact technical conditions that lead to the nesting property.

Suppose for a moment that the true model for the data follows the models in Section 2.2. We consider either p=10 instruments or p=50 instruments with the following $\gamma_{i}$ values:

1. *Many weak instruments*: $\gamma_{i}$ are spread out evenly on the interval 0 to 0.2.
2. *Many strong instruments*: $\gamma_{i}$ are spread out evenly on the interval 1 to 4.
3. *Many very weak instruments, one medium strength instrument*: $\gamma_{i}$, i=1,2,…,p−1, are evenly spread out on the interval 0 to 0.01, and $\gamma_{p}=0.2$.
4. *Many medium strong instruments, one strong instrument*: $\gamma_{i}$, i=1,2,…,p−1, are evenly spread out on the interval 1 to 1.2, and $\gamma_{p}=4$.

The first scenario mimics typical magnitudes of $\gamma_{i}$ seen in MR studies where many genetic traits weakly contribute to the expression of complex traits.^37^, ^38^, ^39^ The third scenario represents a genetic architecture where only few genetic variants have strong effects on the exposure and the rest have weak effects.^40^ Scenarios 2 and 4 are similar to scenarios 1 and 3, but with larger coefficients. We don't expect to observe scenarios 2 and 4 in practice, but the magnitudes of γs in these scenarios were shown from Section 3.1 to produce informative bounds when p=1.

**FIGURE 3:**
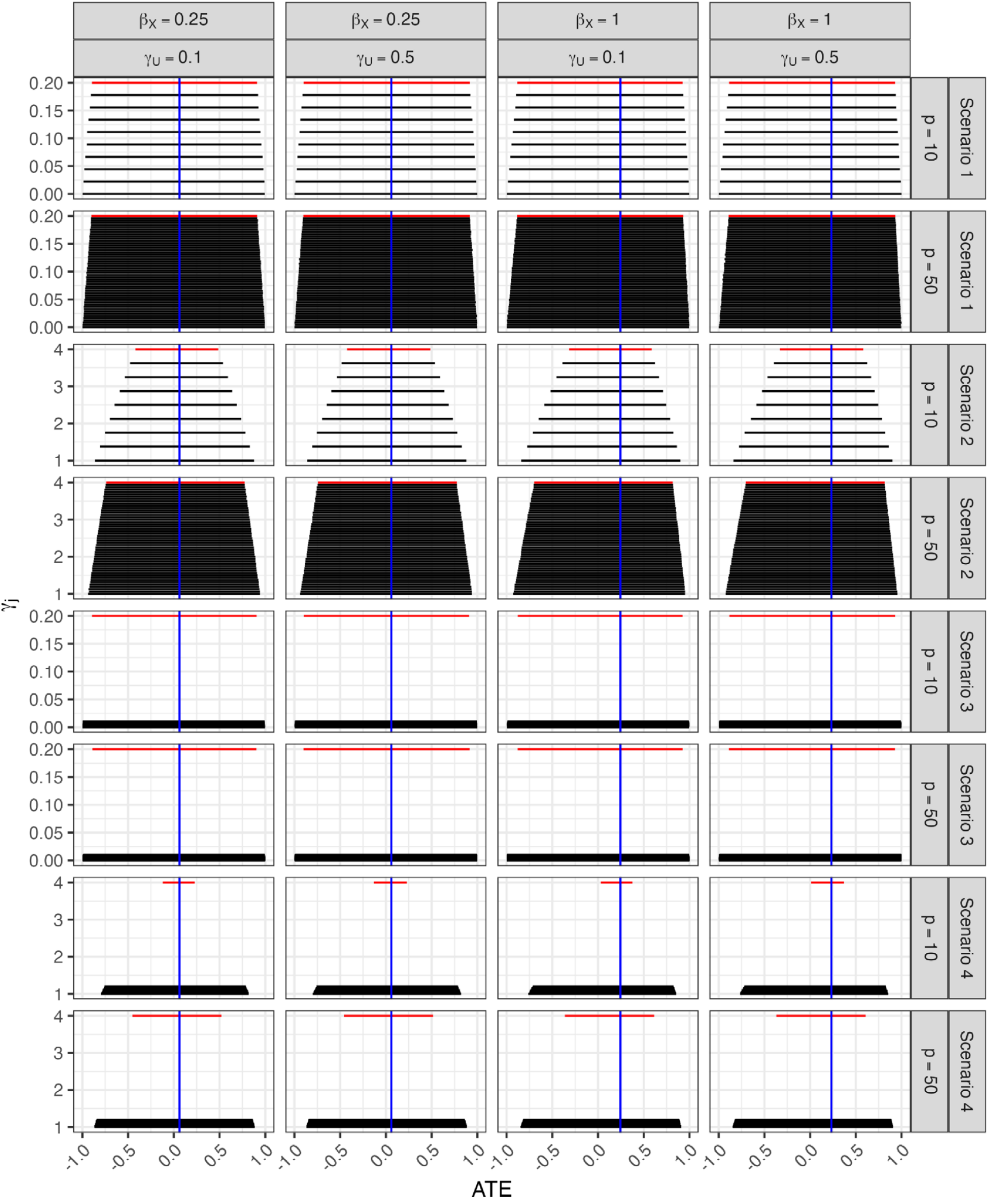
Two‐sample bounds with p=10 or p=50 instruments. Bounds from strongest instruments are highlighted in red. Blue lines denote the true average treatment effects (ATEs). Columns represent effect size of the exposure and the unmeasured confounder on the outcome on the logit scale. Rows represents different scenarios of multiple instruments. The y‐axis represents instrument strength measured by $\gamma_{j}$ and the x‐axis represents the average treatment effect

For each scenario, we use Monte Carlo integration to obtain $P(X=1|Z_{j}=z_{j})$ and $P(Y=1|Z_{j}=z_{j})$ from the data generating model. We then use these quantities to obtain two‐sample IV bounds for each of the *p* instruments. Figure 3 shows the results for $\beta_{X}=0.25$ and $\beta_{X}=1$, but similar trends are observed for $\beta_{X}=0.5$, $\beta_{X}=1.5$, or $\beta_{X}=2$; see Appendix F. In all scenarios, the bounds are nested within each other. Thus, if we were to aggregate bounds by taking intersections, the length of the intersection bounds will only be as strong as the bounds from the strongest instrument. Also, all bounds are noninformative, except for scenario 4 when p=10 and $\beta_{X}=1$.

Our results also have dire implications when some instruments turn out to be invalid. If, as suggested by Swanson,^21^ we take the union of IV bounds instead of intersections so that the union bound is guaranteed to cover the true ATE so long as there is at least one valid instrument, the union bound will likely be noninformative because there was at least one IV bound in our scenario that was noninformative. Without making some assumptions about the nature of the invalid IVs when multiple IVs are used, a bound‐based analysis will likely not reveal any useful information about the ATE.

Overall, combining our results in Section 3.1, our conclusion about using nonparametric IV bounds in two‐sample MR studies is grim. A useful bound‐based analysis would require very strong instruments and/or effect sizes; relatedly, the instruments must be stronger than those from one‐sample studies. Also, multiple instruments are no better than having a single, strong instrument.

### 3.3 Pleiotropy

A major concern in MR studies is pleiotropy, which is a violation of the exclusion restriction (A3). In particular, a practical concern is that when (A3) is violated, the two‐sample IV bounds may still produce bounds about the ATE, say in terms of length or detecting the sign of the effect, and mislead investigators about the magnitude or direction of the ATE. Or, the verifiable constraints that are part of the bounds may fail to detect the violation of (A3) and again, mislead investigators about the presence of an invalid instrument. To this end, we reuse the exposure model specified in (2) with p=1, and use the following outcome model:

(6) $$\text{logit}(P(Y=1|X=x,Z=z,U=u))=\beta_{0}+\beta_{X}x+\beta_{Z}z+\beta_{U}u.$$

**FIGURE 4:**
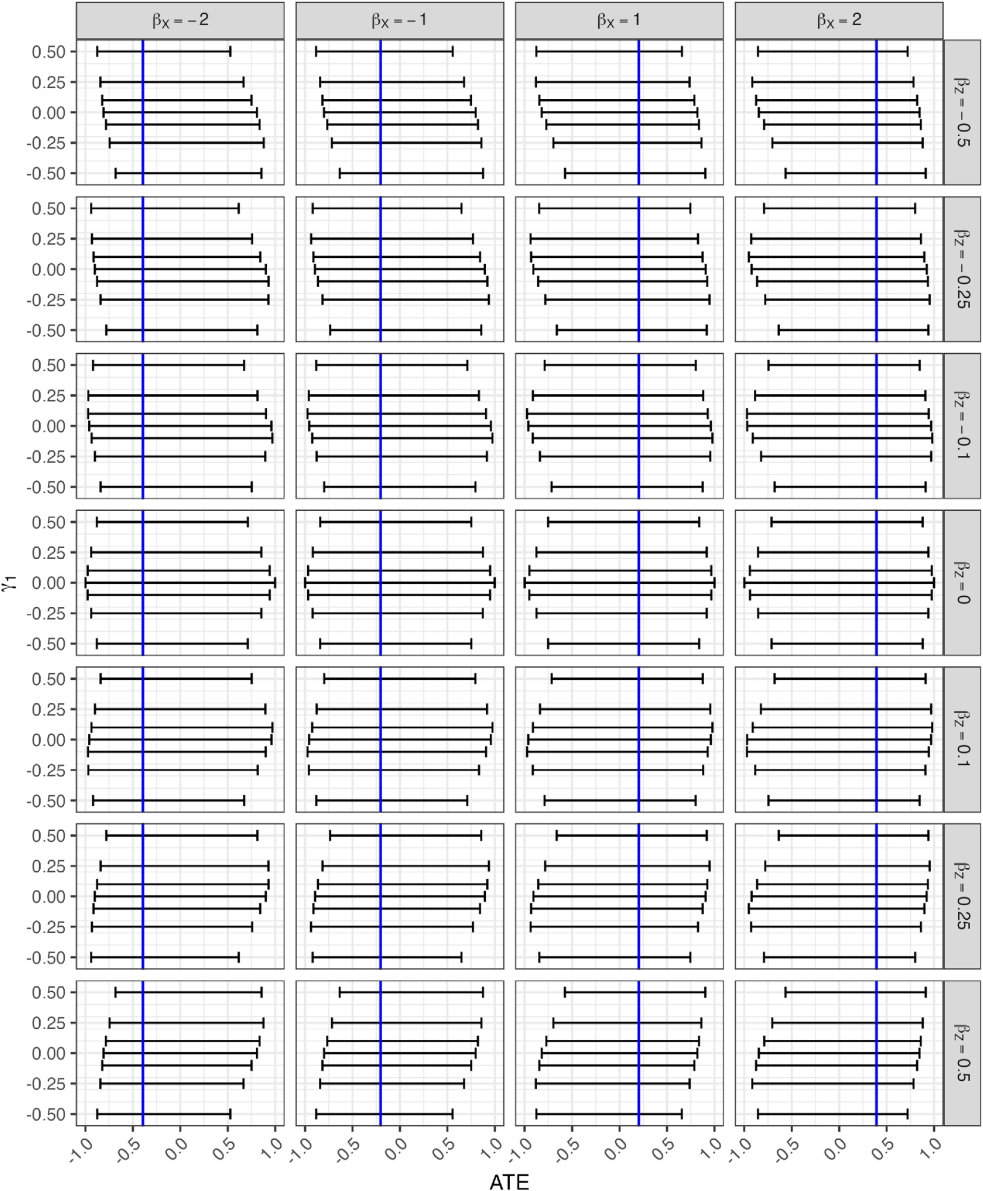
Two‐sample bounds (horizontal lines) and average treatment effects (vertical blue lines) under pleiotropy. Columns represent the effect size of the exposure on the logit scale, rows represent the magnitude of violation of assumption (A3). The x‐axis shows average treatment effect (ATE), and the y‐axis represents instrument strength as measured by $\gamma_{i}$

We set the coefficients $\beta_{X}\in \{-2,-1,0,1,2\},\beta_{Z}\in \{-0.5,-0.25,-0.1,0,0.1,0.25,0.5\},\beta_{0}=-\beta_{X}/2,\gamma_{1}\in \{-0.5,-0.25,-0.1,0,0.1,0.25,0.5\}$, and $\gamma_{0}=-\gamma_{1}$ while $\gamma_{U}=\beta_{U}=1$. Figure 4 shows the results. In every single scenario, the bounds do in fact cover the ATE. That is, weak instruments effectively dominate the behavior of the bounds, including any biases that may be incurred from a pleiotropic instrument, and produce wide bounds. Also, the verifiable constraints in Equation (5) are never violated, suggesting that they are limited in their ability to detect violations of the assumptions when instruments are weak and in general, subject‐matter knowledge may be a more powerful argument for (or against) the validity of an instrument. Appendix G provides additional results concerning the effect of pleiotropy on the bounds.

### 3.4 Dichotomizing a continuous exposure and effect on two‐sample bounds

In many MR studies, the exposure is often a continuous variable and using a bound‐based analysis on such studies require dichotomizing the exposure variable. For example, in Section 5 where we study the effect of cholesterol on heart disease, we use a dichotomized exposure variable in order to use a bound‐based analysis. Dichotomizing the exposure variable raises many important questions ranging from the interpretability of the estimator for the exposure effect to whether the estimator is actually estimating a causal quantity.^22^ In this section, we show the effect of dichotomization on two‐sample bounds by showing that if the exposure has a monotonic effect on the outcome, the two‐sample bounds using a dichotomized exposure can be used to detect the sign of the underlying causal effect.

Let $\tilde{X}$ be a continuous exposure, and ${\tilde{Y}}^{\tilde{x}}$ be a potential outcome under the continuous exposure $\tilde{x}$. We define the dichotomization of the exposure as $X=1[\tilde{X}\geq c]$ for some *c*, and link the potential outcome under a binary exposure and the continuous exposure as $Y^{1}={\tilde{Y}}^{\tilde{x}}$ for $\tilde{x}\geq c$ and $Y^{0}={\tilde{Y}}^{\tilde{x}}$ for $\tilde{x}<c$. Without loss of generality, under the monotonicity assumption where the outcome increases as the exposure increases, that is, $P({\tilde{Y}}^{\tilde{x}}\leq {\tilde{Y}}^{\tilde{x}+\epsilon })=1$ for all $\tilde{x}$ and ϵ>0, we have for any ${\tilde{x}}^{\prime }>\tilde{x}$, $\text{sign}\left(E[{\tilde{Y}}^{{\tilde{x}}^{\prime }}-{\tilde{Y}}^{\tilde{x}}]\right)=\text{sign}\left(E[Y^{1}-Y^{0}]\right)=1$; see Appendix H for a formal argument.

**FIGURE 5:**
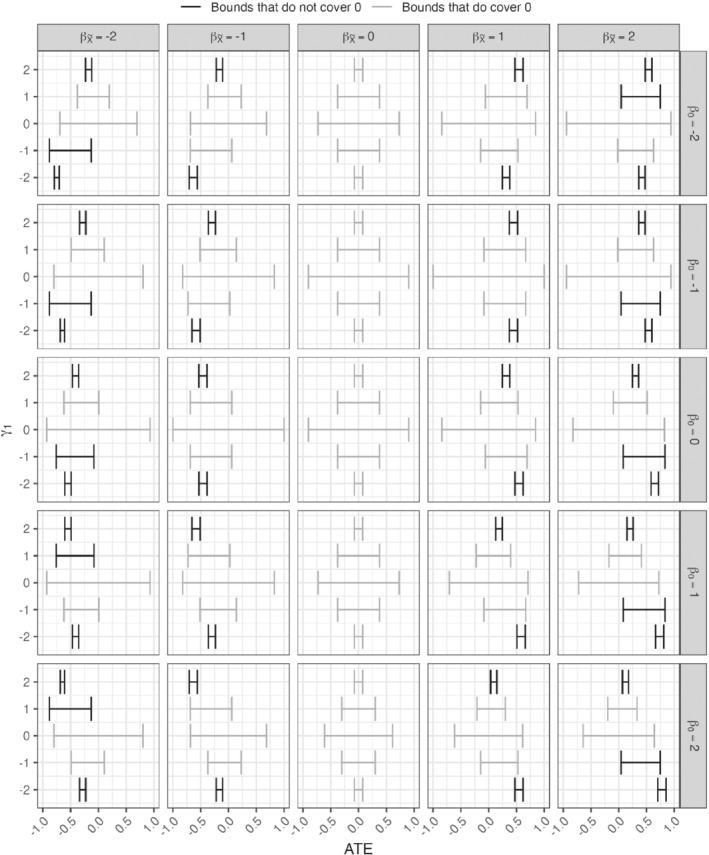
Nonparametric bounds based on a dichotomized exposure. Columns represent the effect size of the exposure on the logit scale. Rows represent different values of the intercept $\beta_{0}$. The y‐axis shows the effect of the instrument on the continuous exposure, and the x‐axis shows the average treatment effect

We numerically illustrate the result through a simulation study. We use a linear model for the continuous exposure with $\gamma_{0}=0$, and a logistic model for the outcome. We generate a data set with 10,000,000 observations, dichotomize the exposure using the observed median of the exposure, and then calculate the nonparametric two‐sample IV bounds. Figure 5 show parts of the results; the full set of results can be found in Appendix H. We see that whenever the two‐sample IV bounds based on the dichotomized exposure allow us to make conclusions about the direction of the exposure effect, the inferred direction is the same as the direction of the effect of the continuous exposure.

### 3.5 Finite‐sample behavior of bounds with estimated probabilities

The nonparametric bounds discussed above are all derived assuming population level probabilities are available. In practice, we estimate the probabilities from a sample using logistic regression; see Section 2.3, Section 5, Appendix K, and Appendix I. In particular, in Appendix I, we show numerically that for all practical values of instrument strength in MR, incorporating additional uncertainty from estimation leads to bounds with 100% empirical confidence levels. Only when the instruments are implausibly strong do we see loss in coverage. This result is not surprising since we have shown above that two‐sample IV bounds without accounting for estimation error are often wide and noninformative and adding additional uncertainty from estimation will inevitably enlarge these bounds; see section 7 in Reference 19 for a similar observation.

## 4 CHARACTERING THE LOSS OF INFORMATION IN TWO‐SAMPLE MR STUDIES

As hinted in Theorem 1, the increase in the bound's length is an inevitable “cost” of using two‐sample designs instead of one‐sample designs in MR studies. This section examines this loss of information by creating a plausible range of the joint distribution of the outcome and the exposure given the instrument, P(X=x,Y=y|Z=z), based on the observed data from two‐sample MR studies; as mentioned before, this joint distribution determines the IV bounds in one‐sample designs and seeing how much of it can be inferred from two‐sample designs provide us with a way to characterize the loss of information from using two‐sample designs.

Formally, the joint conditional distribution P(X=x,Y=y|Z=z) can be written as a function of the marginal conditional distributions P(X=x|Z=z) and P(Y=y|Z=z) from two‐sample MR studies and the conditional covariance of the exposure *X* and outcome *Y* given the instrument *Z*, that is, Cov(X,Y|Z=z), via the following formula

(7) P(X=x,Y=y|Z=z)=P(X=x|Z=z)P(Y=y|Z=z)+(2·I[x=y]−1)Cov(X,Y|Z=z).

Because Cov(X,Y|Z=z) is impossible to estimate from two‐sample MR studies, we take a random sample from −1 to 1, akin to placing a flat, uniform prior on −1 to 1. The sampled value of Cov(X,Y|Z=z) must not only produce a proper probability distribution of (X,Y|Z), but also satisfy the verifiable constraints from the IV assumptions. Specifically, Cov(X,Y|Z=z) must satisfy

$$\begin{array}{llll} \underset{z}{\max}\left\{\begin{array}{l} -\;P(X=1|Z=z)P(Y=1|Z=z)\\ -\;P(X=0|Z=z)P(Y=0|Z=z)\\ P(X=1|Z=z)P(Y=0|Z=z)-1\\ P(X=0|Z=z)P(Y=1|Z=z)-1 \end{array}\right\} & \; & & \\ \leq \text{Cov}(X, & Y|Z=z)\leq & & \\ & \underset{z}{\min}\left\{\begin{array}{l} 1-P(X=1|Z=z)P(Y=1|Z=z)\\ 1-P(X=0|Z=z)P(Y=0|Z=z)\\ P(X=1|Z=z)P(Y=0|Z=z)\\ P(X=0|Z=z)P(Y=1|Z=z) \end{array}\right\}.\; & & \end{array}$$

Also, for any pair of $\left(z,z^{\prime })\in \{0,1,2\}\times \{0,1,2\right\}$, the values of Cov(X,Y|Z=z) and $\text{Cov}(X,Y|Z=z^{\prime })$ must satisfy

$$\begin{array}{ll} \max\left\{\begin{array}{l} -\;P(X=0|Z=z)P(Y=0|Z=z)-P(X=0|Z=z^{\prime })P(Y=1|Z=z^{\prime })\\ P(X=1|Z=z)P(Y=0|Z=z)+P(X=1|Z=z^{\prime })P(Y=1|Z=z^{\prime })-1\\ P(X=0|Z=z^{\prime })P(Y=0|Z=z^{\prime })+P(X=0|Z=z)P(Y=1|Z=z)-1\\ -\;P(X=1|Z=z^{\prime })P(Y=0|Z=z^{\prime })-P(X=1|Z=z)P(Y=1|Z=z) \end{array}\right\}\;\; & \\ \;\leq \text{Cov}(X,Y|Z=z)-\text{Cov}(X,Y|Z=z^{\prime })\leq \;\;\; & \\ \min\left\{\begin{array}{l} 1-P(X=0|Z=z)P(Y=0|Z=z)-P(X=0|Z=z^{\prime })P(Y=1|Z=z^{\prime })\\ P(X=1|Z=z)P(Y=0|Z=z)+P(X=1|Z=z^{\prime })P(Y=1|Z=z^{\prime })\\ P(X=0|Z=z^{\prime })P(Y=0|Z=z^{\prime })+P(X=0|Z=z)P(Y=1|Z=z)\\ 1-P(X=1|Z=z^{\prime })P(Y=0|Z=z^{\prime })-P(X=1|Z=z)P(Y=1|Z=z) \end{array}\right\}. & \end{array}$$

We sequentially sample values of Cov(X,Y|Z=0),Cov(X,Y|Z=1),Cov(X,Y|Z=2), such that the above inequalities are satisfied. Among such samples, we calculate the joint distribution of P(X=x,Y=y|Z=z) using (7), leading us to a plausible set of values for the joint distribution P(X=x,Y=y|Z=z).

For each plausible joint distribution P(X=x,Y=y|Z=z), we use the one‐sample IV bounds^13^, ^16^ to obtain a bound for the ATE. If a large number of one‐sample IV bounds obtained from this procedure does not cover zero, then there is some evidence for a nonzero exposure effect and a one‐sample MR study may yield informative bounds on the ATE. However, if a large number of the one‐sample IV bounds covers zero, there is little hope of obtaining information about the ATE if we used a one‐sample MR design; in other words, the one‐sample IV bounds are equally likely to be conservative as the two‐sample IV bounds. This approach can be extended to intersection bounds based on multiple instruments; see Appendix J. For convenience, the approach is implemented as a Shiny web application for investigators to use at https://rtrane.shinyapps.io/potential‐one‐sample‐bounds.

**FIGURE 6:**
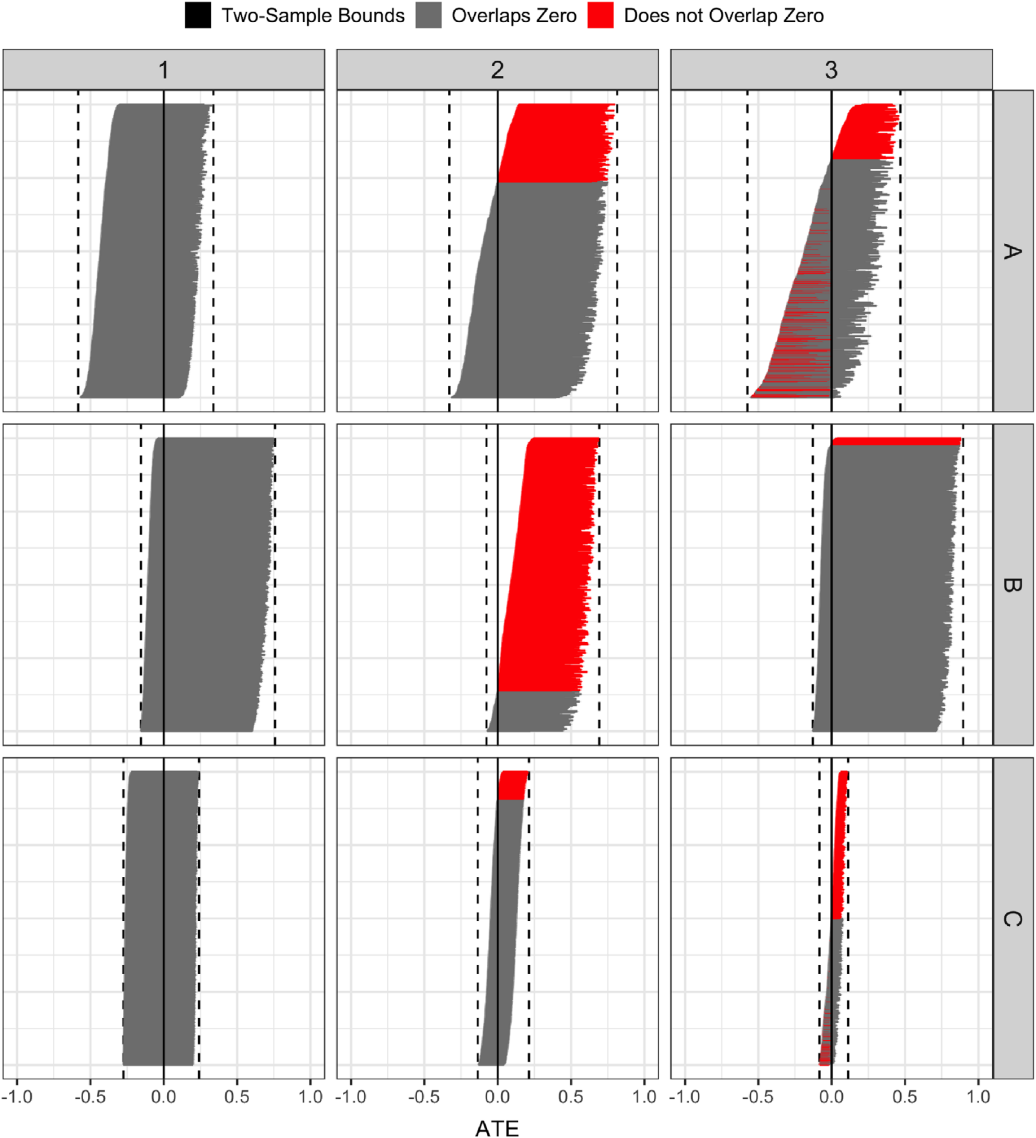
One‐sample bounds (horizontal lines) and two‐sample bounds (vertical dotted lines). Red color represents one‐sample bounds that do not cover zero and gray color represents one‐sample bounds that do cover zero

Table 1 presents nine different sets of values of the marginal distributions P(Y|Z) and P(X|Z) that investigators could theoretically obtain from hypothetical two‐sample MR studies. Figure 6 shows the one‐sample IV bounds from the procedure we illustrated above.

**TABLE 1:** Values of P(X=1|Z=z) and P(Y=1|Z=z) used to illustrate our approach. For each cell (eg, row A, column 1), we have {P(X=1|Z=0),P(X=1|Z=1),P(X=1|Z=2)} on the first row and {P(Y=1|Z=0),P(Y=1|Z=1),P(Y=1|Z=2)} on the second row

	Column 1	Column 2	Column 3
Row A	{0.125, 0.399, 0.080} {0.699, 0.840, 0.742}	{0.244, 0.275, 0.185} {0.238, 0.089, 0.146}	{0.603, 0.469, 0.310} {0.638, 0.346, 0.719}
Row B	{0.886, 0.968, 0.874} {0.805, 0.822, 0.951}	{0.139, 0.441, 0.334} {0.179, 0.359, 0.559}	{0.901, 0.909, 0.935} {0.821, 0.810, 0.905}
Row C	{0.175, 0.079, 0.365} {0.599, 0.358, 0.087}	{0.493, 0.911, 0.085} {0.360, 0.480, 0.441}	{0.434, 0.045, 0.733} {0.747, 0.370, 0.169}

Row A of Figure 6 shows three scenarios where the two‐sample bounds are all centered close to zero with similar length. But, the conclusions from the one‐sample bound analysis are rather different. Column 1 shows no one‐sample bounds would allow us to determine the presence of a nonzero exposure effect. Column 2 indicates that about 26.3% of the one‐sample IV bounds do not contain 0 while for column 3 that number is approximately 35.9%. However, the latter includes one‐sample bounds entirely above and below 0.

Row B illustrates three scenarios where the two‐sample bounds are centered well above zero and have large length. We see one case where we have no hope of determining direction of the ATE from the one‐sample bounds (column 1), one case where we are most likely to determine the ATE's direction (column 2), and one case where we are unlikely to determine the ATE's direction (column 3).

Row C is similar to row A in that all the two‐sample bounds are centered around 0, but the lengths of the two‐sample bounds are narrow. The three columns indicate similar conclusions as row A, showing that even with rather narrow two‐sample bounds centered around 0, the one‐sample bounds may not reveal information about presence or the direction of the exposure effect.

Overall, the proposed procedure and the examples above show that a bound‐based analysis could have been useful had we used a one‐sample design compared to a two‐sample design. Nevertheless, we mention a word of caution when interpreting the results above, especially concerning the sampling of the covariance values. For example, a scenario like the one resulting in the bounds presented in row B, column 2 only provides information about the plausibility of different one‐sample bounds; it does not provide the probability of each bound not covering zero. However, if the true ATE is in fact negative, the proposed procedure does rule out the possibility of one‐sample bounds being able to ascertain this because all one‐sample bounds covering a negative ATE also covers 0.

## 5 USING BOUND‐BASED ANALYSIS IN TWO, POSITIVE CONTROL EXAMPLES

We demonstrate our findings about the behavior of two‐sample IV bounds on two real MR studies. Our first study examines the effect of smoking on incidence of lung cancer and our second study examines the effect of selfreported high cholesterol status on incidence of heart attack. The effect of smoking on lung cancer is known to be strong and positive.^41^ Also, while the exact mechanism between high cholesterol and heart disease is still being discussed,^42^, ^43^ some meta‐analyses of randomized clinical trials on the effect of cholesterol‐lowering medication suggest a strong causal relationship.^34^, ^44^ In both cases, we assess what conclusions can be obtained by using bound‐based analyses in studies where the causal effects are strong and positive.

The study data were obtained from the UK Biobank data stored in the integrative epidemiology unit (IEU) GWAS database. We use the TwoSampleMR R package^45^ with the recommended defaults to extract and clean the data. For more details, see Appendix K.

**FIGURE 7:**
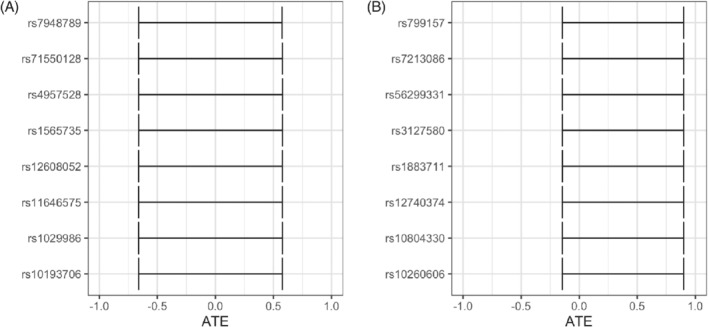
Two‐sample IV bounds for the two real data examples with 8 SNPs from each data set. (A) Two‐sample IV bounds for the ATE of smoking on the incidence of lung cancer. (B) Two‐sample IV bounds for the ATE of high cholesterol on the incidence of heart attack

**FIGURE 8:**
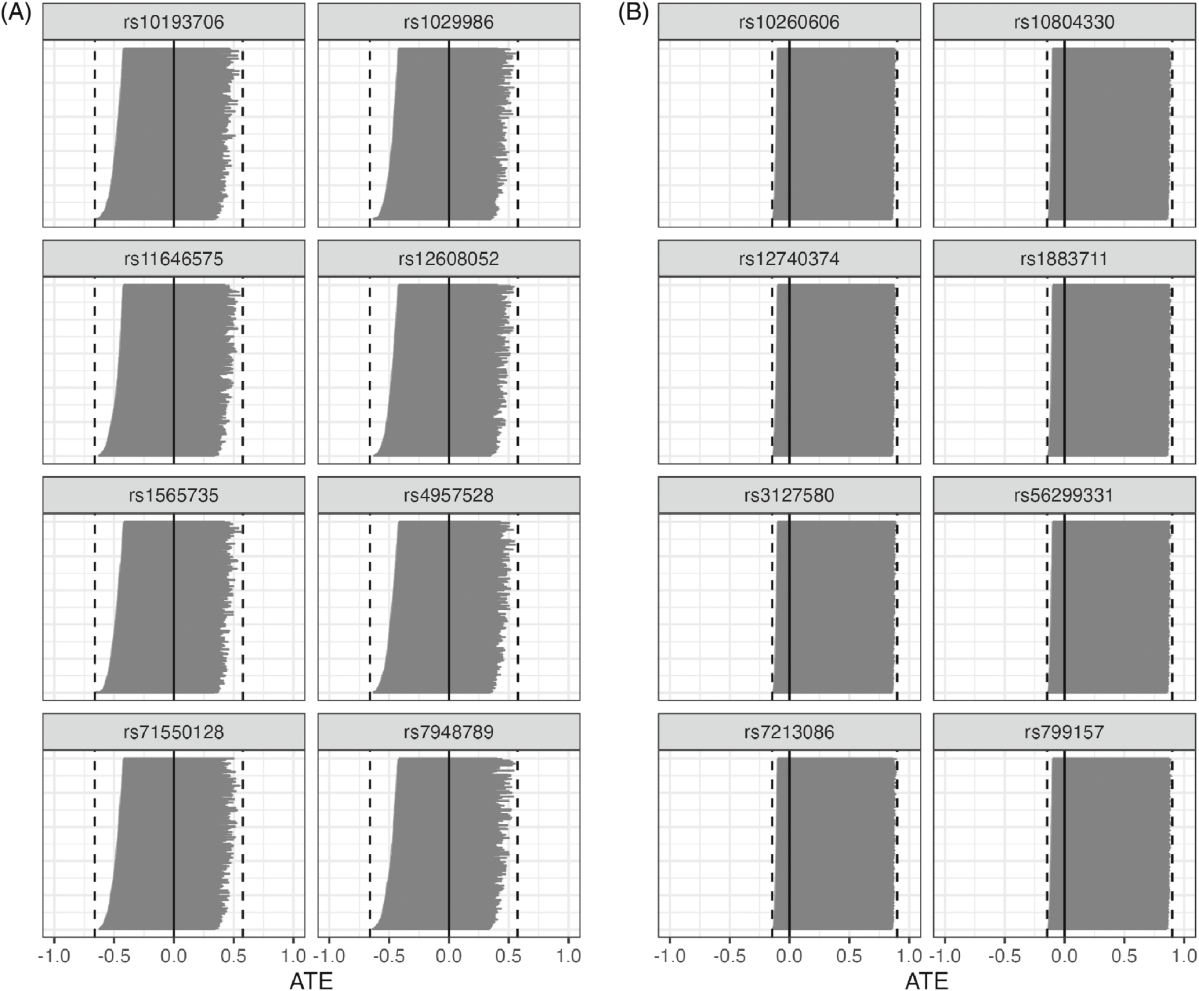
Potential one‐sample IV bounds for the two real data examples using the method described in Section 4. (A) One‐sample IV bounds for the ATE of smoking on the incidence of lung cancer from 500 potential one‐sample distributions for each SNP. (B) One‐sample IV bounds for the ATE of high cholesterol on the incidence of heart attack from 500 potential one‐sample distributions for each SNP

For the effect of smoking on lung cancer, we used 84 genetic instruments, and for the effect of cholesterol on heart attack, we used 54 genetic instruments. The average instrument strengths were 0.0042 (range: 0.0032 to 0.0091) for smoking and 0.0005 (range: 0.0002 to 0.0022) for cholesterol; these values are much smaller than the ST =0.5 needed to guarantee bounds with length less than 1. As such, the two‐sample bounds in Figure 7 are wide; all of them have length greater than 1 and they convey no information about the causal effects of interest. Additionally, using our method from Section 4, the direction of the ATE would not have been detectable had we used a one‐sample design; see Figure 8. Appendix K contains additional analysis, notably demonstrating that aggregating multiple bounds through intersections are also noninformative.

Overall, while nonparametric bounds allow us to not make parametric assumptions frequent in two‐sample MR analyses, they may provide little, if any, information about the exposure effects, even if the exposure effect is known to be positive and strong. Additionally, since many two‐sample MR studies involve weak instruments, we believe bound‐based approaches will likely have limited practical value to uncover causal effects.

## 6 DISCUSSION

Nonparametric bounds are without a doubt an attractive concept. With a minimal set of assumptions, they let investigators obtain bounds on the average treatment effect. However, as we have seen above, in typical MR studies with two‐sample summary data, a bound‐based analysis may generally be uninformative for two reasons. First, while IV bounds in one‐sample settings have length always less than 1, in two‐sample settings, this is not always the case, and the bounds are often more conservative. Second, many genetic variants in MR studies are too weakly associated with the exposure to produce bounds with length less than 1 or bounds that exclude 0. Indeed, our two real data examples showed that despite having strong causal effects, bound‐based analyses were unable to detect these effects.

We also outlined an approach to roughly quantify the information loss going from one‐sample designs to two‐sample designs and to assess the range of conclusions that can be drawn if we had one‐sample data. We demonstrate our method to a few different settings of two‐sample data and showed the range of conclusions that can be drawn about the plausible one‐sample nonparametric bounds. Investigators can also use our Shiny web application to compare one‐sample and two‐sample designs for bound‐based analysis.

Overall, our general recommendation for practice is that unless investigators have a very strong instrument, ideally exceeding ST>0.5, bounds will unlikely be useful as a nonparametric analysis of the ATE, even with multiple instruments. Even if ST>0.5, one would need strong IVs and/or strong effect sizes to make sure that the bounds do not cover 0. Finally, investigators can use our procedure above to assess whether it is worthwhile to use a one‐sample MR design over a more typical (and arguably easier) two‐sample MR design as the bounds under a one‐sample design is generally less conservative than bounds from a two‐sample design.

Nevertheless, there may be few limited, but meaningful use cases for using bounds to study the ATE in two‐sample MR studies; see Reference 33 for one example based on IV inequalities. First, when one has prior knowledge about the direction of the effect, but wish to get a better sense of its magnitude, nonparametric bounds can provide an upper limit on this magnitude. For example, when the exposure is known to cause harm or benefit, say in our smoking example, an upper bound on this effect would tell investigators about the maximum possible effect that smoking could have on increasing the incidence of lung cancer. Second, two‐sample IV bounds can be used to check estimates from parametric methods to see if they lie inside of the bounds; if the estimates lie outside of the bounds, then the parametric models underlying the estimates are likely mis‐specified.

We also note that as part of our study into two‐sample bounds, we encountered bounds where the upper bound is smaller than the lower bound in the two‐sample IV bounds above as well as existing formula for one‐sample IV bounds with multilevel instruments; see Appendix B for more details. In particular, we never see this behavior when the instrument is binary for both one‐ and two‐sample data. Also, when the instrument takes on three values, we never encounter this scenario in one‐sample bounds. But, when the instruments take on three values and we have two‐sample data, we do see it in 0.84% of the 10,000,000 unique probability distributions we tried. When the instrument takes on four categories, this behavior occurs in 0.04% of 10,000,000 unique probability distributions for one‐sample data and 1.27% of 10,000,000 unique probability distributions for two‐sample data. Note that all of these bounds passed the existing falsification constraints Equation (5). Our conjecture is that existing works on one‐sample and two‐sample bounds are correct, but the existing falsification inequalities derived from these bounds may not be tight enough under nonbinary instruments to detect potential violations of the the IV assumptions. That is, both the one‐sample and two‐sample bounds can be computed irrespective of whether the IV assumptions are satisfied or not. But, the current falsification inequalities under nonbinary IV settings may not leverage all parts of the observed data to detect violations of the IV assumptions and it may be possible to use this behavior in the resulting bounds as another falsification test in nonbinary IV settings. We leave this interesting topic as future research.

## Supporting information


**Appendix S1** Supplementary Material

## Data Availability

The data that support the findings of this study are openly available in the UK Biobank stored in the integrative epidemiology unit (IEU) GWAS database. Data on smoking was obtained from the data entry ID ukb‐d‐20116_0, data on lung cancer was from data entry ID ukb‐d‐40001_C349, data on cholesterol was from data entry ID ukb‐a‐108, and data on heart attack was from data entry ID ukb‐a‐434.
