# Supplementary material for: Nonparametric bounds in two‐sample summary‐data Mendelian randomization: Some cautionary tales for practice
Source: Stat Med. 2022 Mar 30;41(14):2523–41. doi: 10.1002/sim.9368 (PMC9314714; doi:10.1002/sim.9368)
Supplement: Supplementary file 1 — Appendix S1 Supplementary Material [file SIM-41-2523-s001.pdf]

APPENDIX

This document contains the Appendix to our paper "Non-parametric Bounds in Two-Sample Summary-Data Mendelian Randomization: Some Cautionary Tales for Practice". This includes additional details on how the ST measure relates to the concentration parameter; how we obtain bounds on the Average Treatment Effect; proof of Theorem 1; additional details and results for the simulation behind the "power" analysis presented in Section 3.1; the technical conditions that lead to the nesting property of bounds discussed in Section 3.2; more results supporting our comments made in Section 3.2 in regards to the usefulness of multiple instruments; the full set of results illustrating the effect of pleiotropy on the coverage of nonparametric bounds from two-sample MR studies; full details on the effect of using a dichotomized exposure variable; simulations showing the finite-sample behavior of the nonparametric bounds from two-sample MR data; details on extending the reconstruction of one-sample distributions introduced in Section 4 to intersection bounds; summary statistics, and complete results for the two example analyses presented in Section 5. For convenience, we include a table of contents below.

A: "Relating ST to the Concentration Parameter"
B: "Bounds on Average Treatment Effect"
C: "Proof of Theorem"
D: "Simulation Setup and Results"
E: "Nestedness of Nonparametric Bounds under Monotonicity"
F: "Would Multiple Instruments Help? Additional Figures"
G: "Coverage Under Pleiotropic Genetic Markers"
H: "Dichotomized Exposure Variable"
I: "Finite-Sample Behavior of Bounds With Estimated Probabilities"
J: "Sampling of Intersection Bounds From Two Instruments"
K: "Additional Summary Statistics and Figures for Analyses"

A RELATING ST TO THE CONCENTRATION PARAMETER

Stock et al.[28] present a different measure of the strength of an instrument called the concentration parameter. We will here see how our measure of strength ST $= \max_{z\neq z'} |P(X=1|Z=z) - P(X=1|Z=z')|$ can be related to the concentration parameter.

The concentration parameter is defined based on a linear model of the relationship between the exposure and the instrument. Here, we will consider the model with an intercept[46]

$$E[X \mid Z] = \gamma_0 + \gamma_1 Z + \epsilon,$$

where γ_0, γ_1 are real-valued parameters and Z is the vector of values of the instrument, i.e. $z_i \in \{0,1,2\}$. Note that we treat the above model as a working model and not a true model in roder to define the concentration parameter under a binary exposure. The concentration parameter is then defined as

$$\mu^2 = \gamma_1 Z' Z \gamma_1 / \sigma^2 = \gamma_1^2 \sum_{i=1}^{n} z_i^2 / \sigma^2$$

Notice that in this model, $P(X=1|Z=z) = \gamma_0 + \gamma_1 z$, meaning that ST $= 2|\gamma_1|$. Hence, larger values of ST coincides with larger values of μ^2.

B BOUNDS ON AVERAGE TREATMENT EFFECT

We briefly review the method presented by[18] to bound the average treatment effect using two-sample summary data. Let $\vec{\tau}^* = \Big(P(Y=1|X=0,U), P(Y=1|X=1,U), P(X=1|Z=0,U), ..., P(X=1|Z=k-1,U)\Big) \in [0,1]^{2+k}$ and $\vec{v}^* = \Big(P(Y=0|Z=0,U), ..., P(Y=1|Z=k-1,U), P(X=0|Z=0,U), ..., P(X=1|Z=k-1,U), \alpha^*\Big)$ where

$$\alpha^* = P(Y=1|X=1,U) - P(Y=1|X=0,U).$$

Since $U \perp Z$, $E_U[P(X=x|Z=z,U)] = P(X=x|Z=z)$ and $E_U[P(Y=y|Z=z,U)] = P(Y=y|Z=z)$. Let $\vec{v} = E_U[\vec{v}^*] = \Big(P(Y=0|Z=0),...,P(Y=1|Z=k-1),P(X=0|Z=0),...,P(X=1|Z=k-1),\alpha\Big)$, where

$$\begin{aligned}\alpha &= E_U[P(Y=1|X=1,U) - P(Y=1|X=0,U)] \\ &= E[Y^1] - E[Y^0] = \text{ATE}.\end{aligned}$$

Note that while $\vec{\tau}^*$ and $\vec{v}^*$ are both entirely unobervable, $\vec{v}$ consists of k observable values, and one unobservable value, the ATE.

By the exclusion restriction, we have

$$P(X=x,Y=y|Z=z,U) = P(Y=1|X=x,U)P(X=x|Z=z,U),$$

which means we can define a mapping $f : [0,1]^{2+k} \mapsto \mathcal{V}$ such that $f(\vec{\tau}^*) = \vec{v}^*$ as

$$f(y_0,y_1,x_0,x_1,...,x_{k-1}) = \begin{pmatrix} (1-y_0)\cdot(1-x_0)+(1-y_1)\cdot x_0 \\ y_0\cdot(1-x_0)+y_1\cdot x_0 \\ \vdots \\ (1-y_0)\cdot(1-x_{k-1})+(1-y_1)\cdot x_{k-1} \\ y_0\cdot(1-x_{k-1})+y_1\cdot x_{k-1} \end{pmatrix}$$

We define $\mathcal{V} = f([0,1]^{2+k})$.

Since $\vec{v} = E_U[\vec{v}^*]$, $\vec{v}$ must be a convex combination of $\vec{v}^*$. Let $\mathcal{H}$ be the convex hull of $\mathcal{V}$. Then $\vec{v}$ will be in $\mathcal{H}$.

Now, let $\hat{\mathcal{T}}$ be the set of extreme vertices of $[0,1]^{2+k}$, $\hat{\mathcal{V}} = f(\hat{\mathcal{T}})$, and $\hat{\mathcal{H}}$ be the convex hull of $\hat{\mathcal{V}}$. By Theorem 1 in Appendix B of[18], $\mathcal{H} = \hat{\mathcal{H}}$. This means that $\vec{v} \in \hat{\mathcal{H}}$. Utilizing a program such as Polymake, we can describe $\mathcal{H}$ with a set of inequalities, which give us constraints that $\vec{v}$ must satisfy.

This means that we can obtain inequalities that the components of $\vec{v}$ must satisfy by describing the extreme vertices of $[0,1]^{2+k}$, map them to $\mathcal{V}$ using the relatively simple function f, and then use polymake to find inequalities that characterize the convex hull of $f([0,1]^{2+k})$. This gives us a set of inequalities involving the components of $\vec{v}$. Some of these will be verifiable, as they will not include the only unobservable quantity α. Others will not be verifiable, but will allow us to obtain bounds on the unobservable quantity α using the observable entries of $\vec{v}$.

Following the approach from Ramsahai (2012) as outlined above, we obtain bounds on the average treatment effect from the quantities $P(X=1|Z=z)$ and $P(Y=1|Z=z)$, $z=0,1,2$. To do so, we first write down the most extreme values of each of $P(Y=1|X=x,U)$ and $P(X=x|Z=z,U)$ for all $x=0,1$, $z=0,1,2$. Since these are probabilities, the extreme values are 0 and 1.

TABLE B1 Most extreme values of $P(Y=1|X=x,U)$ and $P(X=1|Z=z,U)$. Here, PY1XxU $= P(Y=1|X=x,U)$ and PX1ZzU $= P(X=1|Z=z,U)$.

PY1X0U	PY1X1U	PY1Z0U	PX1Z1U	PX1Z2U
0	0	0	0	0
0	0	0	0	1
0	0	0	1	0
0	0	0	1	1
0	0	1	0	0
0	0	1	0	1
0	0	1	1	0
0	0	1	1	1
0	1	0	0	0
0	1	0	0	1
0	1	0	1	0
0	1	0	1	1
0	1	1	0	0

PY1X0U	PY1X1U	PY1Z0U	PX1Z1U	PX1Z2U
0	1	1	0	1
0	1	1	1	0
0	1	1	1	1
1	0	0	0	0
1	0	0	0	1
1	0	0	1	0
1	0	0	1	1
1	0	1	0	0
1	0	1	0	1
1	0	1	1	0
1	0	1	1	1
1	1	0	0	0
1	1	0	0	1
1	1	0	1	0
1	1	0	1	1
1	1	1	0	0
1	1	1	0	1
1	1	1	1	0
1	1	1	1	1

By applying the function f to each row, we get the most extreme vertices of $P(X = x|Z = z, U)$, $P(Y = y|Z = z, U)$, and α for all $x = 0, 1,\ y = 0, 1$ and $z = 0, 1, 2$.

TABLE B2 Most extreme values of $P(Y = y|Z = z)$ and $P(X = x|Z = z)$. Here, PYyZz $= P(Y = y|Z = z)$, PXxZz $= P(X = x|Z = z)$, and $\alpha = P(Y = 1|X = 1, U) - P(Y = 1|X = 0, U)$.

PY0Z0	PY0Z1	PY0Z2	PY1Z0	PY1Z1	PY1Z2	PX0Z0	PX0Z1	PX0Z2	PX1Z0	PX1Z1	PX1Z2	α
1	1	1	0	0	0	1	1	1	0	0	0	0
0	0	0	1	1	1	1	1	1	0	0	0	-1
1	1	1	0	0	0	1	1	1	0	0	0	1
0	0	0	1	1	1	1	1	1	0	0	0	0
1	1	1	0	0	0	0	1	1	1	0	0	0
1	0	0	0	1	1	0	1	1	1	0	0	-1
0	1	1	1	0	0	0	1	1	1	0	0	1
0	0	0	1	1	1	0	1	1	1	0	0	0
1	1	1	0	0	0	1	0	1	0	1	0	0
0	1	0	1	0	1	1	0	1	0	1	0	-1
1	0	1	0	1	0	1	0	1	0	1	0	1
0	0	0	1	1	1	1	0	1	0	1	0	0
1	1	1	0	0	0	0	0	1	1	1	0	0
1	1	0	0	0	1	0	0	1	1	1	0	-1
0	0	1	1	1	0	0	0	1	1	1	0	1
0	0	0	1	1	1	0	0	1	1	1	0	0
1	1	1	0	0	0	1	1	0	0	0	1	0
0	0	1	1	1	0	1	1	0	0	0	1	-1
1	1	0	0	0	1	1	1	0	0	0	1	1
0	0	0	1	1	1	1	1	0	0	0	1	0
1	1	1	0	0	0	0	1	0	1	0	1	0
1	0	1	0	1	0	0	1	0	1	0	1	-1

PY0Z0	PY0Z1	PY0Z2	PY1Z0	PY1Z1	PY1Z2	PX0Z0	PX0Z1	PX0Z2	PX1Z0	PX1Z1	PX1Z2	α
0	1	0	1	0	1	0	1	0	1	0	1	1
0	0	0	1	1	1	0	1	0	1	0	1	0
1	1	1	0	0	0	1	0	0	0	1	1	0
0	1	1	1	0	0	1	0	0	0	1	1	-1
1	0	0	0	1	1	1	0	0	0	1	1	1
0	0	0	1	1	1	1	0	0	0	1	1	0
1	1	1	0	0	0	0	0	0	1	1	1	0
1	1	1	0	0	0	0	0	0	1	1	1	-1
0	0	0	1	1	1	0	0	0	1	1	1	1
0	0	0	1	1	1	0	0	0	1	1	1	0

Theorem 1 of Ramsahai (2012) tells us that the values of $P(X = 1|Z = z)$, $P(Y = 1|Z = z)$, $z = 0, 1, 2$ must lie in the convex hull of the vertices given by the rows in Table B2. This means that the vector of these values must be a convex combination of the rows in said table. Using this with the fact that they must sum to 1 is what enables us to use polymake to find inequalities that the values of $P(X = 1|Z = z)$, $P(Y = 1|Z = z)$, and α must satisfy. In this particular case, these are as presented below. This table should be read as rows of coefficients for which it holds that $\sum_{z=0}^{2} c_{X1Zz} \cdot P(X = 1|Z = z) + \sum_{z=0}^{2} c_{Y0Zz} \cdot P(Y = 0|Z = z) + c_{Y1Z0} \cdot P(Y = 1|Z = 0) + c_\alpha \alpha \geq 0$.

TABLE B3 Results from polymake. Columns with all zeroes have been removed.

c_{Y0Z0}	c_{Y0Z1}	c_{Y0Z2}	c_{Y1Z0}	c_{X1Z0}	c_{X1Z1}	c_{X1Z2}	c_α
2	0	-1	0	2	0	0	-1
1	0	-1	1	0	0	0	0
1	-1	0	1	0	0	0	0
1	-1	0	0	1	1	0	0
1	0	-1	0	1	0	1	0
2	0	-1	1	1	0	-1	-1
2	-1	0	1	1	-1	0	-1
2	0	-2	1	0	0	2	1
2	-1	0	1	-1	1	0	1
4	0	-2	3	0	0	-2	-1
2	-2	0	1	0	2	0	1
4	-1	0	2	-2	0	0	1
4	0	-1	2	-2	0	0	1
2	0	-1	1	-1	0	1	1
1	0	-1	1	0	0	1	1
3	-1	0	2	-1	-1	0	0
2	-1	0	0	2	0	0	-1
4	-2	0	3	0	-2	0	-1
3	0	-1	2	-1	0	-1	0
1	-1	0	1	0	1	0	1
1	-1	1	1	0	1	-1	1
1	0	0	1	0	-1	0	0
1	0	0	1	0	0	-1	0
1	0	1	1	0	0	-1	1
2	-1	2	2	0	0	-2	1
1	1	0	1	0	-1	0	1
0	1	0	1	1	-1	0	1
0	0	1	1	1	0	-1	1

c_{Y0Z0}	c_{Y0Z1}	c_{Y0Z2}	c_{Y1Z0}	c_{X1Z0}	c_{X1Z1}	c_{X1Z2}	c_{α}
2	2	-1	2	0	-2	0	1
2	1	-1	2	0	-1	-1	0
2	-1	1	2	0	-1	-1	0
0	0	0	1	1	0	0	1
1	1	-1	1	0	-1	1	1
0	0	0	0	1	0	0	0
2	0	0	1	-1	0	0	1
0	0	1	1	-1	0	1	-1
0	0	0	0	0	1	0	0
1	-1	1	1	0	-1	1	-1
-1	2	0	0	0	2	0	-1
2	0	-1	2	0	0	-1	-1
1	0	1	3	-2	0	0	-1
1	1	0	2	-1	-1	0	0
0	1	-1	0	0	1	1	0
0	1	0	1	-1	1	0	-1
0	0	1	0	0	0	0	0
-1	0	1	1	2	0	0	1
3	-2	1	3	0	-2	0	-1
0	0	0	0	0	0	1	0
0	-1	1	0	0	1	1	0
0	1	0	0	0	0	0	0
1	1	0	3	-2	0	0	-1
1	0	0	1	-1	0	0	0
0	2	-1	0	0	2	0	-1
1	0	2	2	0	0	-2	1
0	0	0	1	0	0	0	0
1	-2	1	1	0	2	0	1
2	-1	0	2	0	-1	0	-1
1	1	-1	1	0	1	-1	-1
-1	0	1	0	1	0	1	0
1	0	0	0	1	0	0	-1
-1	0	2	0	0	0	2	-1
1	2	0	2	0	-2	0	1
1	1	-2	1	0	0	2	1
-1	1	0	0	1	1	0	0
0	1	0	0	0	1	0	-1
0	0	1	0	0	0	1	-1
1	0	0	2	-1	0	0	-1
-1	1	0	1	2	0	0	1
3	1	-2	3	0	0	-2	-1
0	-1	2	0	0	0	2	-1
1	0	1	2	-1	0	-1	0
1	0	0	0	0	0	0	0

The matrix presented in the table above simplifies to the following set of bounds on the average treatment effect. These are obtained by considering the rows above where $c_{\alpha} \neq 0$.

$$\max\left\{\begin{array}{ll} \max_{i\neq j} & P(Y=1|Z=i) - 2\cdot P(Y=1|Z=j) - 2\cdot P(X=1|Z=j) \\ \max_{i\neq j} & P(Y=1|Z=i) + P(X=1|Z=i) - P(Y=1|Z=j) - P(X=1|Z=j) - 1 \\ \max_{i\neq j} & 2\cdot P(Y=1|Z=i) + 2\cdot P(X=1|Z=i) - P(Y=1|Z=j) - 3 \\ \max_{i} & -P(Y=1|Z=i) - P(X=1|Z=i) \\ \max_{i} & P(Y=1|Z=i) + P(X=1|Z=i) - 2 \end{array}\right\}$$

$$\leq \alpha \leq \tag{B1}$$

$$\min\left\{\begin{array}{ll} \min_{i\neq j} & P(Y=1|Z=i) - 2\cdot P(Y=1|Z=j) + 2\cdot P(X=1|Z=j) + 1 \\ \min_{i\neq j} & P(Y=1|Z=i) + 2\cdot P(Y=1|Z=j) - 2\cdot P(X=1|Z=j) + 1 \\ \min_{i\neq j} & P(Y=1|Z=i) - P(X=1|Z=i) + P(X=1|Z=j) - P(Y=1|Z=j) + 1 \\ \min_{i} & P(X=1|Z=i) - P(Y=1|Z=i) + 1 \\ \min_{i} & P(Y=1|Z=i) - P(X=1|Z=i) + 1 \end{array}\right\} \tag{B2}$$

Furthermore, we obtain the following checkable constraints from the rows where $\alpha = 0$:

$$\min\left\{\begin{array}{ll} \min_{i\neq j} & P(Y=1|Z=i) - P(X=1|Z=i) - P(Y=1|Z=j) - P(X=1|Z=j) + 2 \\ \min_{i\neq j} & P(Y=1|Z=i) + P(X=1|Z=i) - P(Y=1|Z=j) + P(X=1|Z=j) \\ \min_{i} & P(X=1|Z=i) \\ \min_{i} & P(Y=1|Z=i) \\ \min_{i} & 1 - P(X=1|Z=i) \\ \min_{i} & 1 - P(Y=1|Z=i) \end{array}\right\} \geq 0 \tag{B3}$$

We notice that the constraints from the law of probability are recovered (the last four expressions above) along with 12 non-trivial constraints.

These bounds involve 24 different expressions on both the lower and upper end, making an algebraic exploration of the bounds very challenging. However, by imposing two additional monotonicity assumptions, the bounds reduce to just three expressions on the lower end and three expressions on the upper end. This is done by removing rows in the matrix of extreme vertices where the monotonicity assumptions are violated before using Polymake to get the inequalities. The additinal assumptions are

(A5) *(Monotonicity between Z and X)* $P(X=1|Z=z,U) \leq P(X=1|Z=z+1,U)$ for $z=0,1,2$

(A6) *(Monotonicity between Z and Y)* $P(Y=1|Z=z,U) \leq P(Y=1|Z=z+1,U)$ for $z=0,1,2$

and the resulting bounds are

$$\max\left\{\begin{array}{c} P(Y=1|Z=k) - 2\cdot P(Y=1|Z=0) - P(X=1|Z=0) \\ P(Y=1|Z=k) - P(Y=1|Z=0) + P(X=1|Z=2) - P(X=1|Z=0) - 1 \\ 2\cdot P(Y=1|Z=k) - P(Y=1|Z=0) + P(X=1|Z=k) - 2 \end{array}\right\}$$

$$\leq ATE \leq$$

$$\min\left\{\begin{array}{c} P(X=1|Z=0) - P(Y=1|Z=0) + 1 \\ P(Y=1|Z=k) - P(Y=1|Z=0) + P(X=1|Z=0) - P(X=1|Z=k) + 1 \\ P(Y=1|Z=k) - P(X=1|Z=k) + 1 \end{array}\right\}$$

It should be noted that imposing these additional assumptions do not tighten the bounds, it only simplifies the expressions.

We encountered one surprise when studying the behavior of the bounds in (B1). Of 10,123 randomly generated sets of values for $P(X=1|Z=z), P(Y=1|Z=z),\ z=0,1,2$, 123 resulted in bounds where the upper limit is smaller than the lower limit without violating any of the verifiable constraints presented in (B3). Table B4 gives the values of the marginal conditional distributions with the strength of the IV, the corresponding bounds, and the length. It is notable that the IVs are rather strong in all cases where we see the bounds flip, but the bounds themselves and the length vary quite a bit.

We first attributed this to the transition from one-sample to two-sample bounds, but later realized similar scenarios arise when dealing with one-sample bounds from four category IVs. We present some of these in Table B5, where we include the one-sample distributions with the strengths of the IVs, and the length of the resulting bounds. Again, it is interesting to see the large span of lengths and strengths present.

To further explore this, we randomly generated $10,000,000$ distributions of both $P(X, Y|Z)$ and $P(X|Z), P(Y|Z)$ for an instrument Z with $k = 2, 3, 4$ categories. In Table B6, we tabulate the one-sample distributions based on whether or not they violate the verifiable constraints, and whether the bounds are flipped. Table B7 is the equivalent table for two-sample distributions. We note that we do not see any flipped bounds for one-sample bounds when the instrument is binary or with three categories that meet the verifiable constraints, but we do see this when the instrument has four categories, albeit very rarely. For two-sample bounds, we see flipped bounds that are not caught by the verifiable constraints even when the instrument has three categories, and in more than one percent of the distributions when the instrument has four categories.

We have been unable to unearth a reason for why we see this phenomenon. One possible explanation is that the distributions that result in flipped bounds violate some uncheckable assumption.

TABLE B4 Marginal conditional probabilities resulting in bounds where the upper bound is smaller than the lower bound.

P(X=1|Z=0)	P(X=1|Z=1)	P(X=1|Z=2)	P(Y=1|Z=0)	P(Y=1|Z=1)	P(Y=1|Z=2)	Strength	Lower Bound	Upper Bound	Length
0.2309955	0.3669268	0.9387298	0.8850137	0.3013143	0.9801302	0.7077343	0.5364056	-0.0067221	-0.5431277
0.9404491	0.4742722	0.1448868	0.0262469	0.5741507	0.1155472	0.7955623	0.0532826	-0.4025552	-0.4558377
0.8243777	0.0826950	0.6396267	0.0984834	0.0536095	0.6267494	0.7416826	0.3541403	-0.0785379	-0.4326782
0.6253430	0.7940521	0.0769966	0.7125237	0.1332569	0.0937761	0.7170556	0.3709784	-0.0341142	-0.4050925
0.4687418	0.9885571	0.0147455	0.4269904	0.0952051	0.1145516	0.9738116	0.1683963	-0.2136943	-0.3820906
0.2384690	0.9589127	0.4551064	0.9411639	0.8220534	0.2995920	0.7204437	0.2623402	-0.1057977	-0.3681380
0.1201855	0.5087544	0.6903413	0.1553146	0.7813318	0.0153936	0.5701558	0.2303316	-0.1312272	-0.3615588
0.0558596	0.8249922	0.5150187	0.1693588	0.0317164	0.6019942	0.7691326	0.1515574	-0.1885458	-0.3401031
0.0601930	0.7105220	0.7764157	0.0349669	0.6138605	0.1288649	0.7162227	0.4235408	0.0910378	-0.3325030
0.9689451	0.3369273	0.0921191	0.9728974	0.3379845	0.6435396	0.8768260	0.5457005	0.2351435	-0.3105570
0.0272617	0.9602504	0.7090107	0.9941238	0.7603751	0.5393045	0.9329888	-0.0980534	-0.3944198	-0.2963664
0.8593575	0.5455747	0.0954651	0.7493743	0.2343858	0.8692962	0.7638924	-0.0169223	-0.3132765	-0.2963542
0.0051370	0.7930864	0.6854693	0.0171757	0.5039197	0.0258429	0.7879494	0.4592943	0.1768274	-0.2824669
0.8095621	0.0899196	0.7315497	0.1398438	0.0112235	0.5721541	0.7196425	0.3698677	0.0884094	-0.2814583
0.0312864	0.5136612	0.7187288	0.1782691	0.7144743	0.0839332	0.6874423	0.2953632	0.0159345	-0.2794287
0.2841081	0.4642261	0.9303618	0.9272837	0.3015191	0.8563395	0.6462537	0.2718836	0.0151680	-0.2567156
0.7020589	0.0426525	0.7537495	0.8146495	0.9551254	0.3030152	0.7110970	-0.2695984	-0.5219304	-0.2523321
0.7299439	0.7079992	0.0126445	0.4179246	0.9411138	0.9059591	0.7172993	-0.1196986	-0.3687044	-0.2490059
0.8553215	0.1611814	0.3987327	0.0868026	0.0650961	0.5766878	0.6941401	0.1241329	-0.1137256	-0.2378585
0.7503627	0.8262444	0.0255938	0.9023691	0.4826617	0.9697816	0.8006505	-0.1771982	-0.4057139	-0.2285157
0.7516532	0.1293625	0.6636683	0.2319998	0.0773707	0.8011377	0.6222907	0.3876713	0.1595554	-0.2281159
0.1892072	0.6542341	0.6029697	0.9717090	0.8941221	0.2186525	0.4650268	-0.1219402	-0.3463509	-0.2244107
0.9351863	0.1648035	0.3655840	0.1803887	0.1576169	0.6793117	0.7703828	0.0344709	-0.1889068	-0.2233777
0.8913881	0.2924893	0.1391987	0.0678851	0.5562612	0.1311623	0.7521894	0.0155394	-0.2032671	-0.2188065
0.2004629	0.8817321	0.4467427	0.2410824	0.0446975	0.7057212	0.6812692	-0.1773694	-0.3797903	-0.2024209
0.2713706	0.9177118	0.2155938	0.0584116	0.0235335	0.5341155	0.7021180	-0.1254488	-0.3224721	-0.1970232
0.1716186	0.9793879	0.4387238	0.0758875	0.0913810	0.4572813	0.8077692	-0.0377310	-0.2332949	-0.1955639
0.0346134	0.8601421	0.5243412	0.7170224	0.9940138	0.4402146	0.8255286	0.2680971	0.0753966	-0.1927005
0.0517557	0.9490455	0.4763609	0.2257054	0.0428283	0.4666474	0.8972898	-0.0882749	-0.2790819	-0.1908070
0.2097271	0.7849572	0.5591844	0.9851851	0.7694310	0.2353843	0.5752301	-0.1266079	-0.3155315	-0.1889237
0.8533233	0.5437889	0.3202183	0.0278734	0.0138157	0.8263378	0.5331050	-0.2888714	-0.4772378	-0.1883664
0.0781475	0.4316186	0.9562902	0.6056942	0.2534086	0.8616394	0.8781427	0.3824505	0.1983152	-0.1841354
0.7343532	0.7111032	0.0863323	0.4004145	0.9342732	0.9323079	0.6480209	-0.1096618	-0.2915366	-0.1818748
0.4855778	0.2600183	0.9736867	0.3390356	0.9283873	0.7874292	0.7136685	0.1831962	0.0022975	-0.1808987
0.6368154	0.0572293	0.8159708	0.5109590	0.0158577	0.1663634	0.7587416	0.3647850	0.1898262	-0.1749588
0.8824330	0.1367268	0.3081087	0.0653359	0.1951474	0.6000460	0.7457061	-0.0637026	-0.2342401	-0.1705375
0.8090247	0.3226145	0.5675011	0.9402684	0.9741885	0.3180210	0.4864103	0.1805653	0.0148730	-0.1656923
0.4510693	0.0872080	0.9033969	0.5323388	0.1710303	0.0969452	0.8161888	0.0158620	-0.1452420	-0.1611040
0.1518352	0.6975145	0.6509167	0.0629987	0.8097783	0.1657477	0.5456793	0.3801104	0.2198838	-0.1602266

TABLE B4 Marginal conditional probabilities resulting in bounds where the upper bound is smaller than the lower bound. *(continued)*

P(X=1|Z=0)	P(X=1|Z=1)	P(X=1|Z=2)	P(Y=1|Z=0)	P(Y=1|Z=1)	P(Y=1|Z=2)	Strength	Lower Bound	Upper Bound	Length
0.0653620	0.3813488	0.9612892	0.9275631	0.4953530	0.7515764	0.8959272	-0.0696219	-0.2290492	-0.1594273
0.2032074	0.7755576	0.4991361	0.7865987	0.9554554	0.2348516	0.5723502	0.2271745	0.0680689	-0.1591056
0.0233274	0.6660489	0.8176706	0.8429973	0.2798561	0.7213751	0.7943432	-0.2017648	-0.3594838	-0.1577189
0.9294752	0.2110150	0.4387583	0.1560685	0.0882931	0.6040925	0.7184602	0.0054762	-0.1509059	-0.1563822
0.1670113	0.6894123	0.4795673	0.0041910	0.8002859	0.0345400	0.5224010	0.4578813	0.3096595	-0.1482218
0.3785346	0.9143229	0.1322393	0.3764540	0.9927913	0.6755701	0.7820836	0.4377743	0.2897923	-0.1479819
0.1776605	0.3763786	0.8762187	0.2525663	0.7852824	0.1601145	0.6985582	-0.0751713	-0.2174909	-0.1423196
0.7676593	0.0086728	0.5238627	0.3109642	0.8841540	0.9821670	0.7589865	-0.2989048	-0.4399984	-0.1410937
0.8834087	0.2154675	0.5237259	0.9402145	0.9094435	0.4479360	0.6679412	0.1993104	0.0599839	-0.1393265
0.2128945	0.6634662	0.7020688	0.9859116	0.2297734	0.8227277	0.4891743	-0.1801804	-0.3162608	-0.1360804
0.8197957	0.4539939	0.2933378	0.1292782	0.6944266	0.0241216	0.5264579	0.0595077	-0.0754615	-0.1349692
0.8932091	0.2573860	0.3789772	0.8683447	0.8850420	0.3218777	0.6358231	0.2012298	0.0665657	-0.1346641
0.3852521	0.7681010	0.1679198	0.6200211	0.0286245	0.1269667	0.6001813	0.0302481	-0.0989742	-0.1292223
0.4450183	0.3448027	0.9580487	0.0334938	0.6223715	0.0373602	0.6132460	-0.3346527	-0.4637484	-0.1290957
0.9626206	0.3323393	0.3615993	0.8971357	0.8947940	0.3577061	0.6302814	0.3618066	0.2327966	-0.1290100
0.9579589	0.2856719	0.2557011	0.0294142	0.0312341	0.4495460	0.7022578	-0.1842660	-0.3066353	-0.1223693
0.2722892	0.1030317	0.9532750	0.3335194	0.0179986	0.1046059	0.8502432	0.0914587	-0.0308574	-0.1223161
0.2075435	0.6267518	0.9907035	0.0610969	0.8711902	0.5325762	0.7831600	0.3339092	0.2125552	-0.1213540
0.1309917	0.9511009	0.6110001	0.0092469	0.1382892	0.3862037	0.8201092	0.1057264	-0.0118269	-0.1175533
0.9469203	0.4771290	0.2975224	0.8483259	0.2756656	0.8366797	0.6493979	0.3148269	0.1973510	-0.1174758
0.9141838	0.3947449	0.2582693	0.1776121	0.6284717	0.0485084	0.6559145	0.0149163	-0.1016151	-0.1165314
0.2539480	0.3283935	0.9257231	0.5855638	0.1211694	0.0074839	0.6717752	-0.3135619	-0.4220422	-0.1084803
0.7554315	0.0394385	0.8166883	0.9193390	0.1504442	0.4920783	0.7772497	0.5395735	0.4314412	-0.1081323
0.5322302	0.8442719	0.1311744	0.7227207	0.1174348	0.2652317	0.7130975	-0.0700917	-0.1763950	-0.1063033
0.1022484	0.7850567	0.3114329	0.9983873	0.9750404	0.6040354	0.6828082	-0.0838413	-0.1882423	-0.1044009
0.8859779	0.1854690	0.2675919	0.9352886	0.8113619	0.3954484	0.7005089	0.2470847	0.1436625	-0.1034222
0.8858413	0.0577413	0.7457014	0.9231434	0.9814877	0.6837953	0.8281000	-0.0658260	-0.1636975	-0.0978715
0.5688937	0.0533840	0.9092544	0.4161218	0.0847550	0.1385937	0.8558704	0.1398438	0.0425567	-0.0972870
0.0111502	0.5785773	0.7360408	0.9491940	0.9715842	0.4417906	0.7248905	-0.3414676	-0.4342969	-0.0928294
0.8016434	0.0919814	0.6269118	0.0598012	0.0080604	0.4024806	0.7096620	0.2023970	0.1138349	-0.0885621
0.5613155	0.3343263	0.9641096	0.1739435	0.9413168	0.6466249	0.6297833	0.0475254	-0.0400375	-0.0875629
0.9421035	0.7800406	0.0170238	0.6536674	0.8584000	0.0860958	0.9250797	0.6521608	0.5647278	-0.0874330
0.4856718	0.1412137	0.8327200	0.2353279	0.7698770	0.8171080	0.6915064	0.0643282	-0.0219988	-0.0863269
0.7587967	0.2217142	0.4642144	0.1261614	0.0095185	0.6397095	0.5370825	0.1772441	0.0950201	-0.0822241
0.8476325	0.0321449	0.5761561	0.7137147	0.9222930	0.4156565	0.8154876	-0.2929622	-0.3646398	-0.0716776
0.8443266	0.0231323	0.6135112	0.5114541	0.9662261	0.9901356	0.8211943	-0.3041605	-0.3747334	-0.0705729
0.7090756	0.0306938	0.8591612	0.8275547	0.1987801	0.4221209	0.8284674	0.3686070	0.2983647	-0.0702424
0.5210445	0.6877412	0.1936365	0.2077578	0.8583608	0.8895555	0.4941047	-0.1155538	-0.1840802	-0.0685264
0.7325333	0.0360979	0.7452189	0.9243027	0.1841382	0.4150783	0.7091209	0.4838304	0.4154162	-0.0684143

TABLE B4 Marginal conditional probabilities resulting in bounds where the upper bound is smaller than the lower bound. *(continued)*

P(X=1|Z=0)	P(X=1|Z=1)	P(X=1|Z=2)	P(Y=1|Z=0)	P(Y=1|Z=1)	P(Y=1|Z=2)	Strength	Lower Bound	Upper Bound	Length
0.3112649	0.5408216	0.7700621	0.0719339	0.8911155	0.9844600	0.4587973	0.4371103	0.3713461	-0.0657642
0.6839198	0.0601158	0.7429099	0.3546209	0.0832522	0.8458772	0.6827941	0.5591411	0.4955250	-0.0636161
0.4925476	0.1475428	0.6432137	0.1357593	0.7295215	0.9418075	0.4956709	0.0342830	-0.0281982	-0.0624812
0.0567614	0.4716677	0.8412115	0.9781020	0.6182925	0.8866750	0.7844501	-0.1625195	-0.2243887	-0.0618691
0.1902110	0.3836209	0.9071890	0.8456573	0.3088491	0.0296753	0.7169780	-0.5392827	-0.6006846	-0.0614020
0.3772296	0.8822068	0.2883994	0.2173902	0.9350335	0.7191264	0.5938073	0.4170904	0.3559363	-0.0611541
0.5973862	0.8450983	0.2624347	0.1392309	0.6156584	0.9712264	0.5826636	-0.2177176	-0.2783525	-0.0606348
0.6339672	0.0297922	0.8123455	0.7376053	0.9506195	0.2630108	0.7825533	-0.5198657	-0.5786439	-0.0587783
0.0823461	0.5840173	0.6679903	0.9677474	0.8284869	0.2712011	0.5856442	-0.4461926	-0.4996015	-0.0534089
0.6535119	0.8883952	0.1073055	0.2820041	0.7154519	0.8117950	0.7810897	-0.0743099	-0.1269749	-0.0526651
0.7404535	0.1312750	0.4474163	0.1314948	0.9068344	0.9347602	0.6091785	-0.3671417	-0.4196239	-0.0524822
0.0820021	0.8994346	0.3178099	0.4734612	0.1446546	0.8253918	0.8174325	-0.2855348	-0.3349518	-0.0494170
0.0143154	0.1408971	0.9883829	0.5259441	0.4011591	0.9257180	0.9740675	0.4270428	0.3779018	-0.0491410
0.5142074	0.8446779	0.0753746	0.5067568	0.0715657	0.1808748	0.7693032	-0.0057421	-0.0529810	-0.0472389
0.1391137	0.4452852	0.7319911	0.0201224	0.4730480	0.0227584	0.5928773	0.1545757	0.1084867	-0.0460890
0.7671998	0.0911903	0.9424491	0.7190755	0.0257481	0.5228183	0.8512587	0.4851985	0.4416630	-0.0435356
0.2249334	0.9771968	0.6502243	0.9434316	0.7995282	0.4743734	0.7522634	0.0790767	0.0373769	-0.0416998
0.9124694	0.5503730	0.0400667	0.7951134	0.6099932	0.9632078	0.8724027	-0.1948275	-0.2362891	-0.0414616
0.1645046	0.8060324	0.5635964	0.9246119	0.7605022	0.3061245	0.6415279	-0.1730552	-0.2140902	-0.0410350
0.7079565	0.5723802	0.2806847	0.8839699	0.2430289	0.9515723	0.4272719	-0.0591760	-0.0987463	-0.0395703
0.2097282	0.9124687	0.2747676	0.2570863	0.1285457	0.7024909	0.7027405	-0.2311382	-0.2703369	-0.0391987
0.9736240	0.0208031	0.3737885	0.9045140	0.4334044	0.2716260	0.9528209	0.4846500	0.4464234	-0.0382266
0.1845828	0.1851770	0.8937890	0.8433725	0.4857333	0.9516657	0.7092062	0.2051761	0.1681541	-0.0370221
0.1904095	0.9898458	0.0778574	0.3241436	0.0396418	0.5826816	0.9119883	-0.4464247	-0.4830894	-0.0366648
0.3058563	0.8758829	0.3221585	0.8338573	0.0715108	0.2981029	0.5700266	-0.4066656	-0.4426015	-0.0359359
0.5517228	0.8850872	0.1379439	0.7797196	0.3208303	0.1888349	0.7471432	0.1261619	0.0917667	-0.0343952
0.0614376	0.2965834	0.9979328	0.0027831	0.1401460	0.0597136	0.9364952	0.0117046	-0.0165844	-0.0282890
0.8779495	0.4096741	0.2304406	0.7998226	0.4274697	0.9938156	0.6475089	-0.0719255	-0.0992804	-0.0273549
0.6979215	0.7737010	0.0234315	0.9852010	0.4651610	0.8182570	0.7502694	-0.0989160	-0.1244899	-0.0255739
0.6623782	0.7107869	0.1608789	0.9024376	0.2805005	0.8890312	0.5499081	-0.1508689	-0.1758042	-0.0249354
0.4107040	0.6300393	0.0755462	0.7135503	0.0247311	0.2318819	0.5544931	0.0986941	0.0758333	-0.0228608
0.2389620	0.9996788	0.3607017	0.1224239	0.2775328	0.6499732	0.7607167	-0.0727986	-0.0942652	-0.0214665
0.2466505	0.3150522	0.9973913	0.7941729	0.4943148	0.9589104	0.7507408	0.4182885	0.3992699	-0.0190186
0.1047963	0.5872602	0.6265764	0.1702907	0.0689137	0.7661262	0.5217801	0.2159521	0.1971807	-0.0187714
0.6454304	0.5477765	0.0021959	0.8270074	0.1628806	0.2007895	0.6432345	0.4210367	0.4032008	-0.0178359
0.0147348	0.9403617	0.7719393	0.1339251	0.5201033	0.7372833	0.9256270	0.4399636	0.4221999	-0.0177637
0.6149141	0.1287129	0.8052456	0.3774013	0.9281094	0.7809966	0.6765327	-0.2049168	-0.2213916	-0.0164747
0.6318831	0.8417779	0.1046526	0.1803197	0.6822984	0.0227946	0.7371254	0.4274041	0.4145748	-0.0128292
0.4658334	0.1177519	0.8202813	0.3008471	0.8740505	0.7295855	0.7025294	-0.2011135	-0.2117500	-0.0106365

TABLE B4 Marginal conditional probabilities resulting in bounds where the upper bound is smaller than the lower bound. *(continued)*

P(X=1|Z=0)	P(X=1|Z=1)	P(X=1|Z=2)	P(Y=1|Z=0)	P(Y=1|Z=1)	P(Y=1|Z=2)	Strength	Lower Bound	Upper Bound	Length
0.4692894	0.9793264	0.2505315	0.6858286	0.3586177	0.0507586	0.7287948	0.0832484	0.0727541	-0.0104943
0.9053262	0.4920161	0.2908324	0.8237065	0.8801458	0.1128271	0.6144939	0.3452384	0.3365678	-0.0086706
0.8400507	0.6066834	0.0207922	0.8392446	0.3014262	0.1199182	0.8192585	0.5578239	0.5502410	-0.0075829
0.2986999	0.3574011	0.7508847	0.7003727	0.1246649	0.9739429	0.4521849	0.3249903	0.3213192	-0.0036711
0.0463115	0.4417234	0.7452841	0.1110238	0.4748895	0.0612693	0.6989726	0.1602189	0.1570808	-0.0031381
0.8543023	0.0104242	0.1896705	0.9925313	0.2311163	0.0674310	0.8438782	0.6262363	0.6260467	-0.0001896

TABLE B5 Lower and Upper limits of bounds where the upper limit is less than the lower limit for trivariate distributions with four category instruments.

Lower	Upper	Strength	Length
0.1796920	0.0395535	0.0853119	-0.1401385
-0.0038326	-0.1264492	0.1539099	-0.1226166
-0.0169573	-0.1304422	0.2235469	-0.1134849
-0.0620851	-0.1743916	0.0805434	-0.1123066
0.0996764	-0.0065497	0.2112420	-0.1062260
-0.0348047	-0.1393748	0.1884223	-0.1045701
-0.0097177	-0.1102060	0.0874967	-0.1004882
-0.0470850	-0.1435686	0.1458296	-0.0964835
-0.1052398	-0.1993785	0.2667633	-0.0941387
0.1097975	0.0268471	0.1774704	-0.0829504
0.1884781	0.1110487	0.3297432	-0.0774293
0.0174359	-0.0580424	0.2058740	-0.0754784
-0.0530855	-0.1187770	0.2521754	-0.0656915
0.0534080	-0.0107149	0.1509847	-0.0641230
-0.0660707	-0.1258819	0.2831483	-0.0598112
0.3495840	0.2945716	0.3633999	-0.0550124
0.1665198	0.1136389	0.2131245	-0.0528809
-0.0356540	-0.0879713	0.2476628	-0.0523173
0.1089847	0.0575836	0.1941017	-0.0514012
0.0086756	-0.0338341	0.2340061	-0.0425097
0.1335166	0.0930974	0.4555966	-0.0404192
0.1163970	0.0761754	0.1573917	-0.0402216
-0.1249197	-0.1611461	0.1712798	-0.0362264
-0.1252239	-0.1581375	0.1035529	-0.0329136
-0.2954311	-0.3273509	0.3077593	-0.0319199
0.0274287	-0.0007244	0.0813449	-0.0281530
-0.1317444	-0.1586467	0.3469784	-0.0269023
0.1050533	0.0818064	0.2388595	-0.0232469
-0.1980031	-0.2156885	0.2205149	-0.0176854
0.0408272	0.0265662	0.1314643	-0.0142609
0.1255375	0.1131666	0.0426523	-0.0123709
-0.1421790	-0.1523644	0.1409053	-0.0101854
-0.0997312	-0.1083943	0.3816466	-0.0086630
-0.0304169	-0.0353880	0.1323408	-0.0049711
0.0094786	0.0046709	0.2838685	-0.0048077
-0.0217285	-0.0245811	0.3531008	-0.0028526
-0.0563955	-0.0583218	0.4092683	-0.0019263

TABLE B6 For each category, 10, 000, 000 distributions of $P(X, Y|Z)$ were randomly generated.

k	Verifiable Constraints Violated	upper < lower	n	Proportion
2	FALSE	FALSE	9611081	0.9611
	TRUE	FALSE	291802	0.0292
	TRUE	TRUE	97117	0.0097
3	FALSE	FALSE	8822427	0.8822
	TRUE	FALSE	682620	0.0683
	TRUE	TRUE	494953	0.0495
4	FALSE	FALSE	7802425	0.7802
	FALSE	TRUE	3797	0.0004
	TRUE	FALSE	1013534	0.1014
	TRUE	TRUE	1180244	0.1180

TABLE B7 For each category, 10, 000, 000 distributions of $P(X|Z)$ and $P(Y|Z)$ were randomly generated.

k	Verifiable Constraints Violated	upper < lower	n	Proportion
2	FALSE	FALSE	8333580	0.8334
	TRUE	FALSE	925808	0.0926
	TRUE	TRUE	740612	0.0741
3	FALSE	FALSE	6000482	0.6000
	FALSE	TRUE	83565	0.0084
	TRUE	FALSE	1513834	0.1514
	TRUE	TRUE	2402119	0.2402
4	FALSE	FALSE	3951817	0.3952
	FALSE	TRUE	127177	0.0127
	TRUE	FALSE	1567644	0.1568
	TRUE	TRUE	4353362	0.4353

C PROOF OF THEOREM

We present the proof of Theorem 1.

First of all, recall that the nonparametric bounds for instruments with $k = 2, 3, 4$ can be written as

$$\max\left\{\begin{array}{ll} \max_{z\neq z'} & P(Y=1|Z=z) - 2\cdot P(Y=1|Z=z') - 2\cdot P(X=1|Z=z') \\ \max_{z\neq z'} & P(Y=1|Z=z) + P(X=1|Z=z) - P(Y=1|Z=z') - P(X=1|Z=z') - 1 \\ \max_{z\neq z'} & 2\cdot P(Y=1|Z=z) + 2\cdot P(X=1|Z=z) - P(Y=1|Z=z') - 3 \\ \max_{z} & -P(Y=1|Z=z) - P(X=1|Z=z) \\ \max_{z} & P(Y=1|Z=z) + P(X=1|Z=z) - 2 \end{array}\right\}$$
$$\leq ATE \leq \tag{C4}$$
$$\min\left\{\begin{array}{ll} \min_{z\neq z'} & P(Y=1|Z=z) - 2\cdot P(Y=1|Z=z') + 2\cdot P(X=1|Z=z') + 1 \\ \min_{z\neq z'} & P(Y=1|Z=z) + 2\cdot P(Y=1|Z=z') - 2\cdot P(X=1|Z=z') + 1 \\ \min_{z\neq z'} & P(Y=1|Z=z) - P(X=1|Z=z) + P(X=1|Z=z') - P(Y=1|Z=z') + 1 \\ \min_{z} & P(X=1|Z=z) - P(Y=1|Z=z) + 1 \\ \min_{z} & P(Y=1|Z=z) - P(X=1|Z=z) + 1 \end{array}\right\}$$

By definition, $\mathrm{ST} = \max_{z,z'} |P(X=1|Z=z) - P(X=1|Z=z')|$. I.e. $\mathrm{ST} = P(X=1|Z=z_{max}) - P(X=1|Z=z_{min})$ where $z_{max} = \arg\max_z P(X=1|Z=z)$ and $z_{min} = \arg\min_z P(X=1|Z=z)$.

Next, for the second expression in the lower bound,

$$\begin{aligned}
&\max_{z\neq z'} P(Y=1|Z=z) - P(Y=1|Z=z') + P(X=1|Z=z) - P(X=1|Z=z') - 1 \\
&\quad \geq P(Y=1|Z=z_{max}) - P(Y=1|Z=z_{min}) + P(X=1|Z=z_{max}) - P(X=1|Z=z_{min}) - 1 \\
&\quad = P(Y=1|Z=z_{max}) - P(Y=1|Z=z_{min}) + \mathrm{ST} - 1
\end{aligned}$$

and therefore the lower bound is bounded below by $\mathrm{LB} \geq P(Y=1|Z=z_{max}) - P(Y=1|Z=z_{min}) + \mathrm{ST} - 1$.

Similarly, for the third expression in the upper bound,

$$\begin{aligned}
&\min_{z\neq z'} P(Y=1|Z=z) - P(Y=1|Z=z') - P(X=1|Z=z) + P(X=1|Z=z') + 1 \\
&\quad \leq P(Y=1|Z=z_{max}) - P(Y=1|Z=z_{min}) - P(X=1|Z=z_{max}) + P(X=1|Z=z_{min}) + 1 \\
&\quad = P(Y=1|Z=z_{max}) - P(Y=1|Z=z_{min}) - \mathrm{ST} + 1
\end{aligned}$$

and so the upper bound is bounded above by $\mathrm{UB} \leq P(Y=1|Z=z_{max}) - P(Y=1|Z=z_{min}) - \mathrm{ST} + 1$.

This leads us to conclude that the length can be bounded from above:

$$\begin{aligned}
\text{length} = \mathrm{UB} - \mathrm{LB} &\leq P(Y=1|Z=z_{max}) - P(Y=1|Z=z_{min}) - \mathrm{ST} + 1 \\
&\quad - \big(P(Y=1|Z=z_{max}) - P(Y=1|Z=z_{min}) + \mathrm{ST} - 1\big) \\
&= 2 - 2\cdot \mathrm{ST}.
\end{aligned}$$

□

D SIMULATION SETUP AND RESULTS

Here we provide details on the simulation used to obtain the results presented in Section 3.1.

Since GWAS results are most often reported as summary statistics and coefficients from a logistic model, we use monte carlo integration to show the relationship between ST and coefficients in a logistic model. We use the model introduced in Section 2.2 with $p = 1$. Throughout, we set $\gamma_0 = -\gamma_1$ and $\beta_0 = -\beta_X/2$. This is done to maximize the differences between probabilities $P(X = 1|Z = z)$, $z = 0, 1, 2$, and $P(Y = 1|Z = z)$, $z = 0, 1, 2$. For simplicity, we also keep $\beta_U = \gamma_U$.

For each combination of values of the coefficients $\gamma_1, \gamma_U, \beta_X$ listed below, $10,000,000$ realizations of the unmeasured confounder U are drawn from a standard normal distribution. For each realization, a value of Z is drawn such that $P(Z = 0) = P(Z = 2) = 0.25$, and $P(Z = 1) = 0.5$. Next, values of X and Y are generated using these values such that $\text{logit}(P(X = 1|Z = z, U = u)) = \gamma_0+\gamma_1 z+\gamma_U u$ and $\text{logit}(P(Y = 1|X = x, U = u)) = \beta_0+\beta_X x+\beta_U u$. This results in $10,000,000$ realizations of (X, Y, Z, U). From these, we find the marginal probabilities $P(X = 1|Z = z)$ and $P(Y = 1|Z = z)$, $z = 0, 1, 2$, the values of $\text{ST} = \max_{z \neq z'} |P(X = 1|Z = z) - P(X = 1|Z = z')|$ and the $\text{ATE} = P(Y = 1|X = 1) - P(Y = 1|X = 0)$.

TABLE D8 The monte carlo integration was performed for all combinations of values of the coefficients γ_1, γ_U, and β_X presented below.

β_1	γ_1	γ_U
0.25, 0.5, 1, 1.5, 2, 4, 6	0.2, 0.4, 0.6, 0.8, 1, 1.2, 1.4, 1.6, 1.8, 2, 2.2, 2.4, 2.6, 2.8, 3, 3.2, 3.4, 3.6, 3.8, 4, 4.2, 4.4, 4.6, 4.8, 5, 5.2, 5.4, 5.6, 5.8, 6	0.1, 0.5, 1, 2

Each set of marginal probabilities leads us to a set of non-parametric bounds from two-sample data. These are shown on Figure D1 together with the ATE, while Figure 1b shows the values of γ_1 plotted against ST.

To find the smallest value of γ_1 that results in bounds excluding 0, we fit a loess curve to the lower bounds in Figure D1, and find the value where this curve crosses 0. This results in the values depicted on Figure 2.

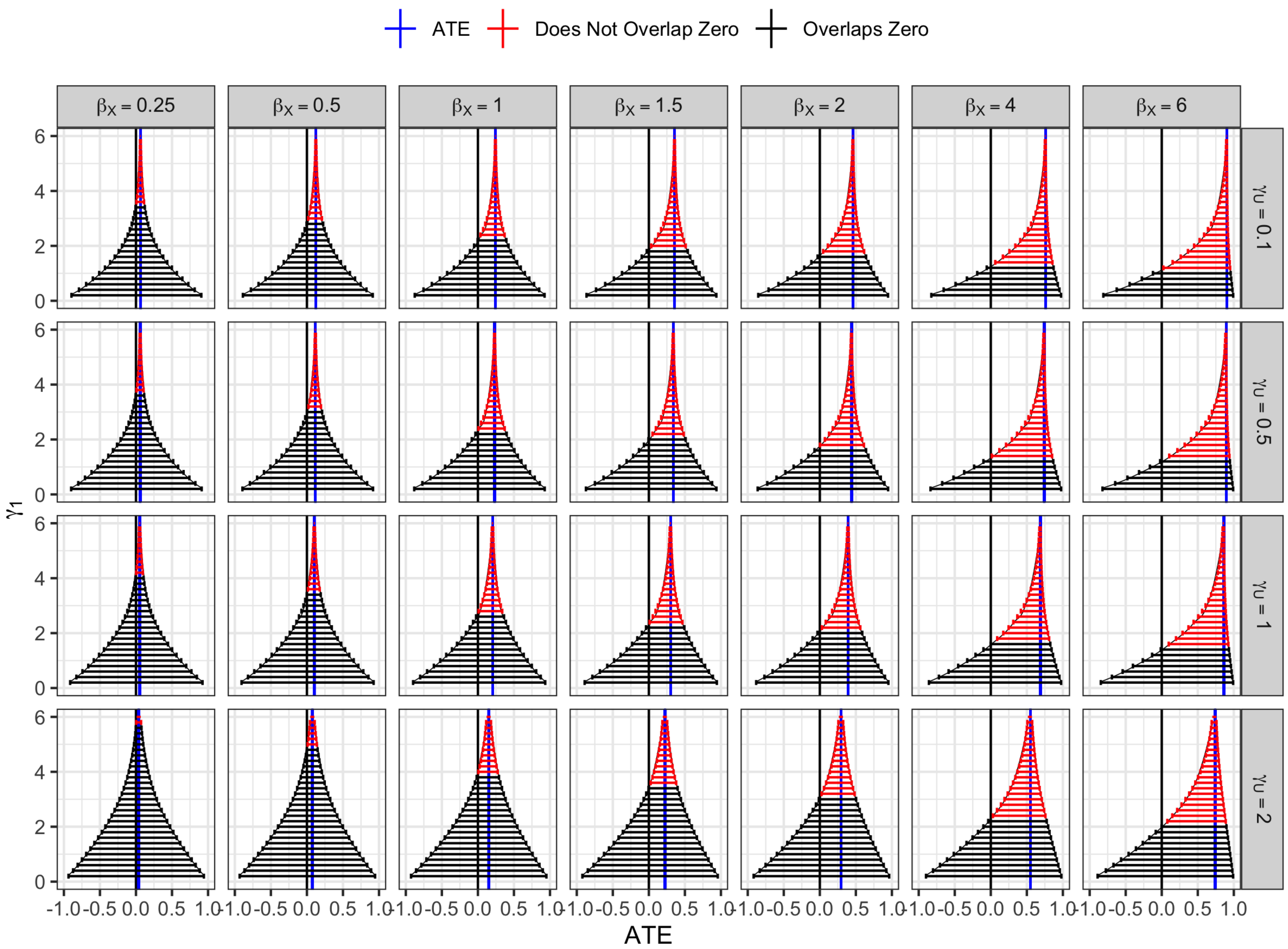

FIGURE D1 Bounds based on simulations as described. Upper and lower bounds are connected by a curve (dotted lines) based on a loess extrapolation. This curve is used to find the smallest coefficients needed to detect direction as plotted on Figure 2.

E NESTEDNESS OF NONPARAMETRIC BOUNDS UNDER MONOTONICITY

Here we will show that the nonparametric bounds are nested as instrument strength increases when the logistic regression model holds for both exposure and outcome, and the instruments are independent and identically distributed. In particular, we assume that

$$\text{logit}(P(X=1|Z_1=z_1,...,Z_p=z_p,U=u))=\gamma_0+\sum_{j=1}^{p}\gamma_j z_j+\gamma_U u,$$

and

$$\text{logit}(P(Y=1|X=x,U=u))=\beta_0+\beta_X x+\beta_U u.$$

Finally, we assume that $P(Z_i=z)=p_z$ for $z=0,1,2$ for any i.

Under the exposure and outcome models specified, assumptions (A5) and (A6) hold. We note that the bounds under (A5) and (A6) can be written as

$$\max\left\{\begin{matrix} P(Y=1|Z=k)-P(Y=1|Z=0)+P(X=1|Z=k)-P(X=1|Z=0)-1 \\ 2\cdot P(Y=1|Z=k)-P(Y=1|Z=0)+P(X=1|Z=k)-2 \\ P(Y=1|Z=k)-2\cdot P(Y=1|Z=0)-P(X=1|Z=0) \end{matrix}\right\}\begin{matrix}(L1)\\(L2)\\(L3)\end{matrix}$$

$$\leq ATE \leq$$

$$\min\left\{\begin{matrix} 1-P(Y=1|Z=0)+P(X=1|Z=0) \\ P(Y=1|Z=k)-P(Y=1|Z=0)-P(X=1|Z=k)+P(X=1|Z=0)+1 \\ P(Y=1|Z=k)-P(X=1|Z=k)+1 \end{matrix}\right\}\begin{matrix}(U1)\\(U2)\\(U3)\end{matrix}$$

when $k=2,3,4$. We can rewrite $P(Y=1|Z=z)$ as a function of $P(X=1|Z=z)$ as follows:

$$\begin{aligned} P(Y=1|Z=z) &= \sum_x P(Y=1|X=x,Z=z)P(X=x|Z=z) \\ &= P(Y=1|X=0,Z=z)\{1-P(X=1|Z=z)\}+P(Y=1|X=1,Z=z)P(X=1|Z=k) \\ &= P(X=1|Z=z)\cdot(P(Y=1|X=1,Z=z)-P(Y=1|X=0,Z=z))+P(Y=1|X=0,Z=z) \\ &= P(X=1|Z=z)\cdot\left(E_U\left[E[Y|X=1,U=u]\right]-E_U\left[E[Y|X=0,U=u]\right]\right)+E_U\left[E[Y|X=0,U=u]\right] \\ &= P(X=1|Z=z)\cdot \text{ATE}+E_U\left[E[Y|X=0,U=u]\right] \end{aligned}$$

Assumptions (A5) and (A6) imply that ATE ≥ 0. Now, we can rewrite the bounds as a function of $P(X=1|Z=k)$, $P(X=1|Z=0)$ and ATE:

$$\max\left\{\begin{matrix} (1+ATE)(P(X=1|Z=k)-P(X=1|Z=0))-1 \\ (2\cdot\text{ATE}+1)P(X=1|Z=k)+E_U\left[E[Y|X=0,U=u]\right]-\text{ATE}\cdot P(X=1|Z=0)-2 \\ P(X=1|Z=k)\cdot\text{ATE}-P(X=1|Z=0)\cdot(2\cdot\text{ATE}+1)-E_U\left[E[Y|X=0,U=u]\right] \end{matrix}\right\}\begin{matrix}(L1)\\(L2)\\(L3)\end{matrix}$$

$$\leq ATE \leq$$

$$\min\left\{\begin{matrix} 1+P(X=1|Z=0)\cdot(1-\text{ATE})-E_U\left[E[Y|X=0,U=u]\right] \\ P(X=1|Z=0)(1-\text{ATE})-P(X=1|Z=k)\cdot(1-\text{ATE})+1 \\ -P(X=1|Z=k)\cdot(1-\text{ATE})+E_U\left[E[Y|X=0,U=u]\right]+1 \end{matrix}\right\}\begin{matrix}(U1)\\(U2)\\(U3)\end{matrix}$$

This means that the lower bound is non-decreasing as a function of $P(X=1|Z=k)$ and non-increasing as a function of $P(X=1|Z=0)$. Since $1-\text{ATE}\geq 0$, the upper bound is non-increasing as a function of $P(X=1|Z=k)$ and non-decreasing as a function of $P(X=1|Z=0)$.

In a logistic model where all instruments $Z_1,...,Z_p$ are independent and have the same distribution, i.e. $P(Z_i=z)=p_z$ for $i=1,...,p$ and $z=0,1,2$, the rank of the coefficients $\gamma_1,...,\gamma_p$ gives the rank of $P(X=1|Z_j=2)$ and $-P(X=1|Z_j=0)$, i.e. the instrument with the largest coefficient has the largest value of $P(X=1|Z_j=2)$ and the smallest value of $P(X=1|Z_j=0)$. To see this, assume without loss of generality $\gamma_1\geq\gamma_j$ for any j. Then

$$\begin{aligned}P(X=1|Z_1=2,Z_2=z_2,...,Z_p=z_p,U=u) &= \text{expit}\left(\gamma_0+2\cdot\gamma_1+\sum_{j=2}^{p}\gamma_j z_j+\gamma_U u\right)\\ &\geq \text{expit}\left(\gamma_0+\gamma_1 z_i+2\cdot\gamma_i+\sum_{j\notin\{1,i\}}^{p}\gamma_j z_j+\gamma_U u\right)\\ &= P(X=1|Z_1=z_i,Z_i=2,Z_2=z_2,...,Z_p=z_p,U=u).\end{aligned}$$

By the law of total expectation,

$$\begin{aligned}P(X=1|Z_1=2,Z_2=z_2,...,Z_p=z_p) &= E[X|Z_1=2,Z_2=z_2,...,Z_p=z_p]\\ &= E_U[E[X|Z_1=2,Z_2=z_2,...,Z_p=z_p,U=u]]\\ &= E_U[P(X=1|Z_1=2,Z_2=z_2,...,Z_p=z_p,U=u)]\\ &\geq E_U[P(X=1|Z_1=z_i,Z_i=2,Z_2=z_2,...,Z_p=z_p,U=u)]\\ &= P(X=1|Z_1=z_i,Z_i=2,Z_2=z_2,...,Z_p=z_p).\end{aligned}$$

Using Bayes' Theorem, the laws of probability, and the independence of the instruments,

$$\begin{aligned}P(X=1|Z_1=2) &= \frac{P(X=1,Z_1=2)}{P(Z_1=2)}\\ &= \frac{\sum_{z_2,...,z_p}P(X=1,Z_1=2,Z_2=z_2,...,Z_p=z_p)}{P(Z_1=2)}\\ &= \sum_{z_2,...,z_p}P(X=1|Z_1=2,Z_2=z_2,...,Z_p=z_p)P(Z_2=z_2)\cdots P(Z_p=z_p)\\ &= \sum_{z_2,...,z_p}P(X=1|Z_1=2,Z_2=z_2,...,Z_p=z_p)p_{z_2}\cdots p_{z_p}\\ &\geq \sum_{z_2,...,z_p}P(X=1|Z_1=z_j,Z_j=2,Z_2=z_2,...,Z_p=z_p)p_{z_2}\cdots p_{z_p}\\ &= P(X=1|Z_j=2)\end{aligned}$$

where the inequality follows from the inequality illustrated above. Similarly, it can be seen that $P(X=1|Z_1=0)\leq P(X=1|Z_j=0)$ for all j. Therefore, Z_1 gives the largest lower bound and smallest upper bound of any instrument. Under (A5) and (A6), instrument strength simplifies to

$$\text{ST}=\max_{z\neq z'}|P(X=1|Z=z)-P(X=1|Z=z')|=P(X=1|Z=k)-P(X=1|Z=0).$$

which means Z_1 is also the strongest instrument. So, under the conditions specified, the strongest instrument will give bounds that are nested in the bounds based on weaker instruments.

F WOULD MULTIPLE INSTRUMENTS HELP? ADDITIONAL FIGURES

The following figures provide the full results described in Section 3.2. Figure F2 illustrate how similarly sized γ_i coefficients lead to weaker instruments when p is increased. Figures F3, F4, F5, and F6 show the full results for all four scenarios.

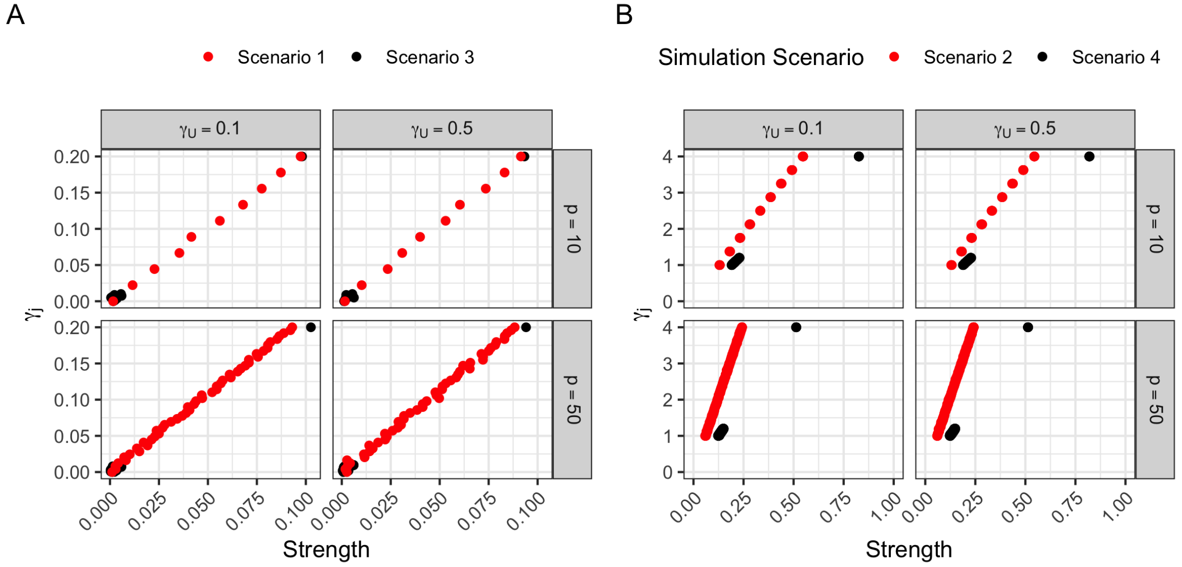

FIGURE F2 Figure showing the dilution effect described in Section 3.2 in each of the four scenarios. When p is larger, similar sized coefficients lead to lower strength. The effect is smaller when we are in a scenario where one coefficient is relatively much larger than the rest, rather than when the coefficients are evenly spread out. A: Scenarios 1 and 3. B: Scenarios 2 and 4.

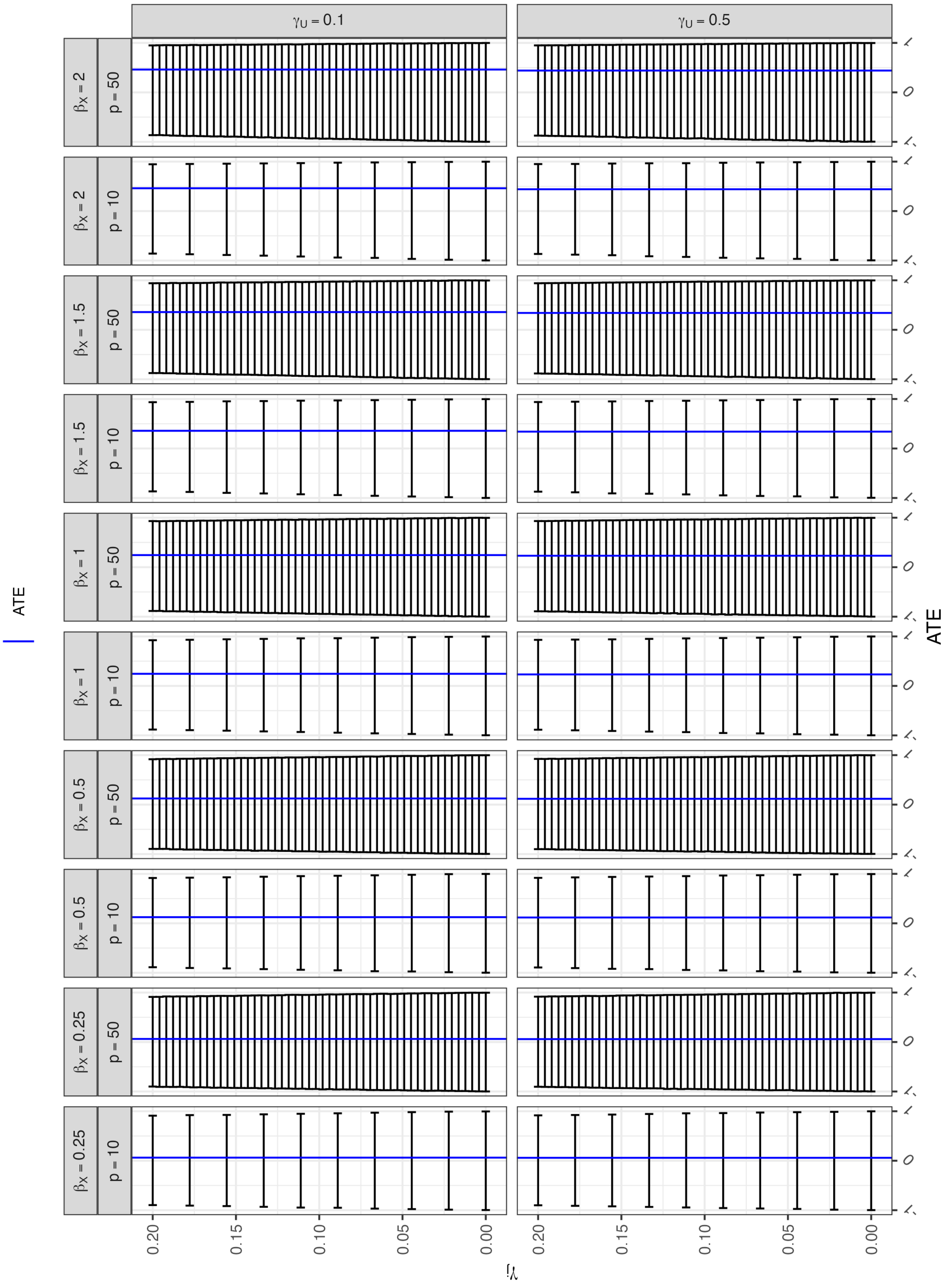

FIGURE F3 Bounds based on monte carlo integration with 1,000,000 resamples in scenario 1.

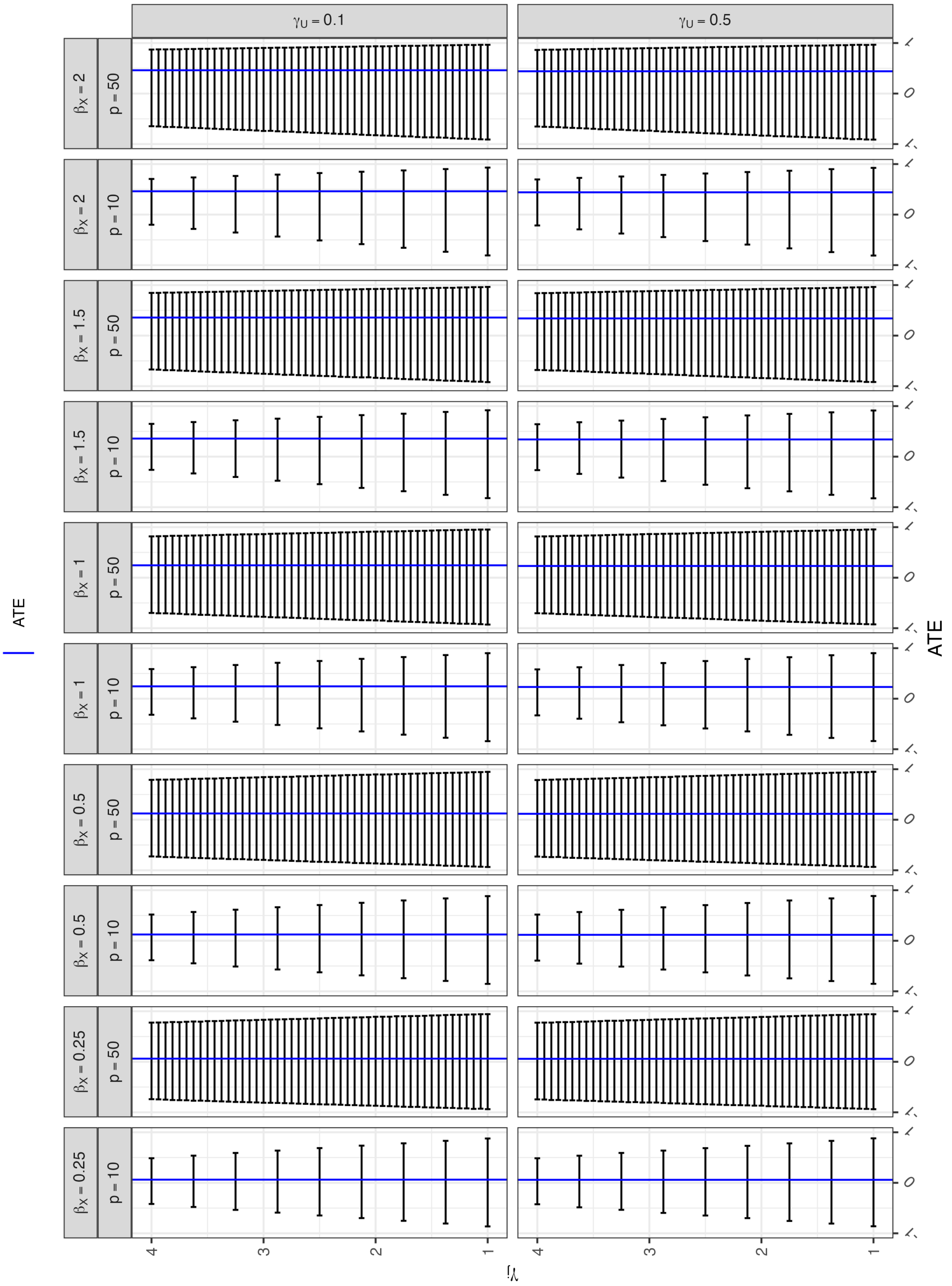

FIGURE F4 Bounds based on monte carlo integration with 1,000,000 resamples in scenario 2.

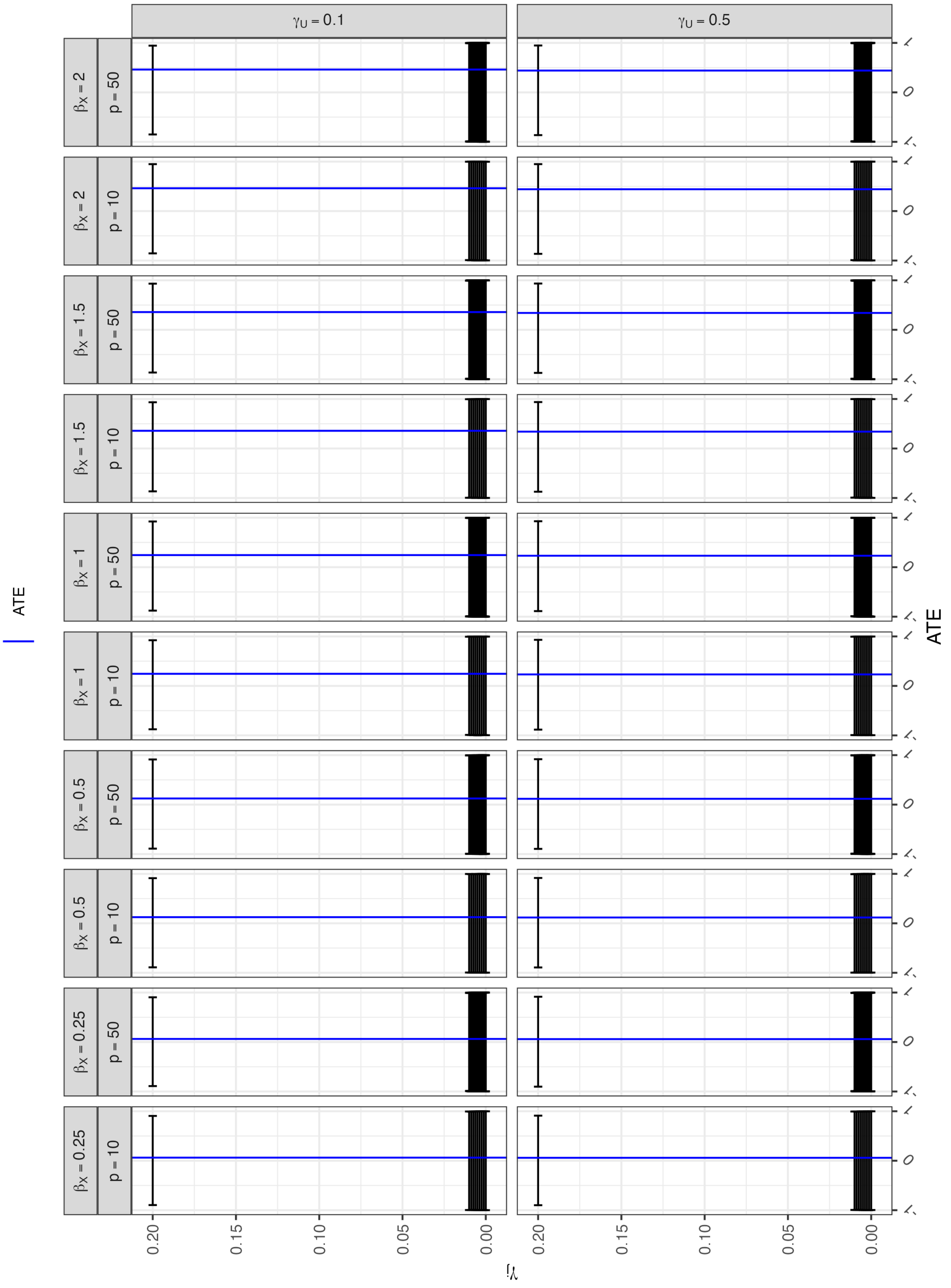

FIGURE F5 Bounds based on monte carlo integration with 1,000,000 resamples in scenario 3.

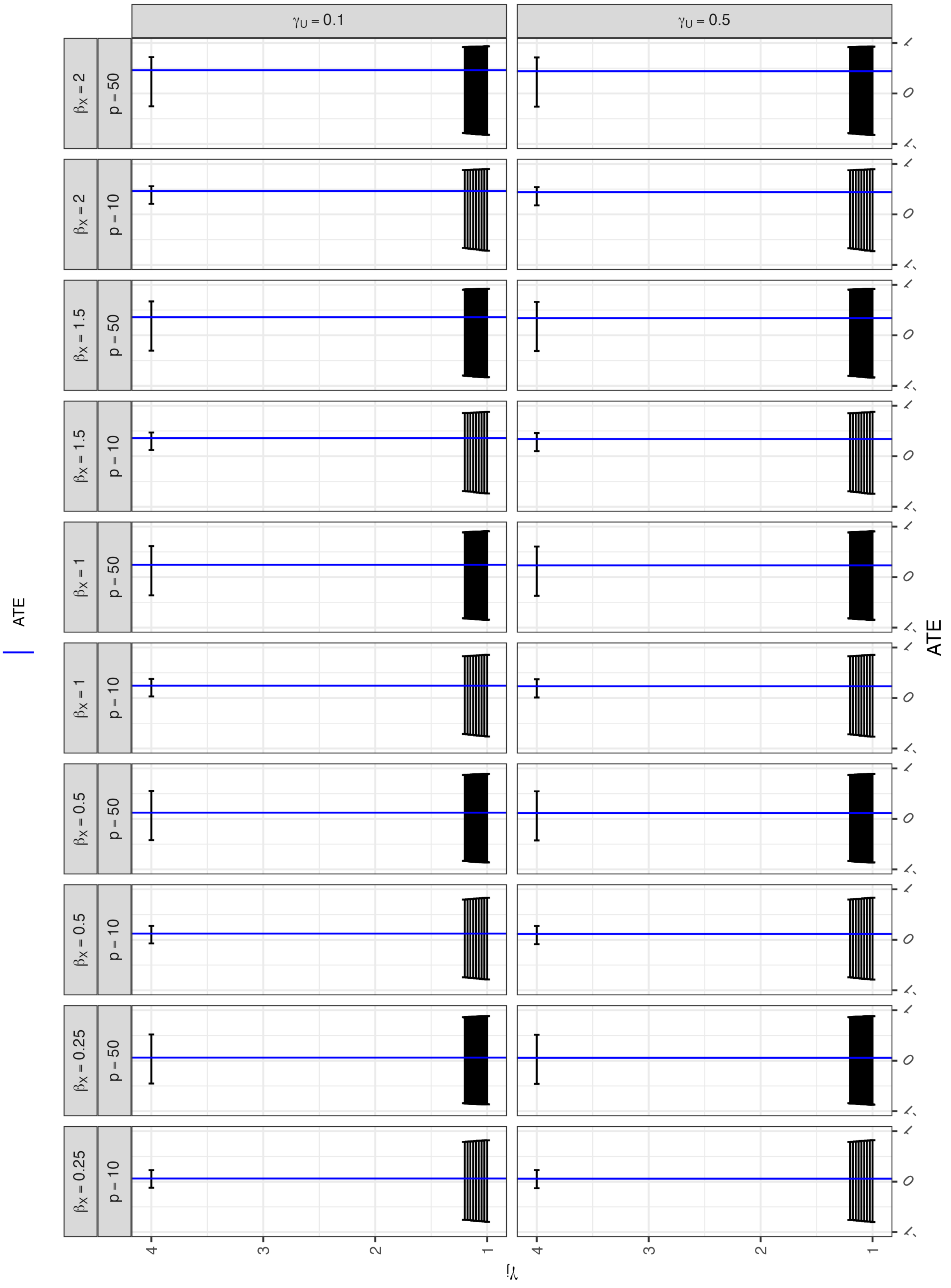

FIGURE F6 Bounds based on monte carlo integration with 1,000,000 resamples in scenario 4.

G COVERAGE UNDER PLEIOTROPIC GENETIC MARKERS

A common concern in MR studies is the possibility of using a genetic marker that is pleiotropic, i.e. that affects the likelihood of both exposure and outcome, and thereby violate the exclusion restriction assumption (A3). To assess the potential damage such a violation might bring, we simulate $10,000,000$ observation from the set of exposure and outcome models described in Section 3.3 with varying values of the coefficients γ_1, β_Z, and β_X. The results are presented on Figures G7 and G8. These figures show the nonparametric bounds (horizontal lines) and actual Average Treatment Effects (solid dots). Blue lines indicate that the bounds do in fact cover the true ATE, while red lines indicate that the bounds do not. Finally, the lines are dotted if any of the verifiable constraints are violated in the given scenario.

These two figures reveal a few noteworthy observations. For coefficients in line with what we see in MR studies (Figure G7), there are two important points to make. First, we see that the ATE is always covered by the bounds. In other words, pleiotropy on the scale we would expect to see in MR analyses do not cause bounds to not cover the ATE. Second, the verifiable constraints are not powerful enough to detect violation of the exclusion restriction in most MR studies.

For Figure G8, we pushed the size of the coefficients to a scale that would provide more information about the power of the verifiable constraints, and the power of the nonparametric bounds. We see that either the effect of the instrument on the outcome or the ATE has to be very large for the bounds to not cover the ATE. Likewise, the effect of the instrument on the outcome has to be large for the verfiable constraints to be violated.

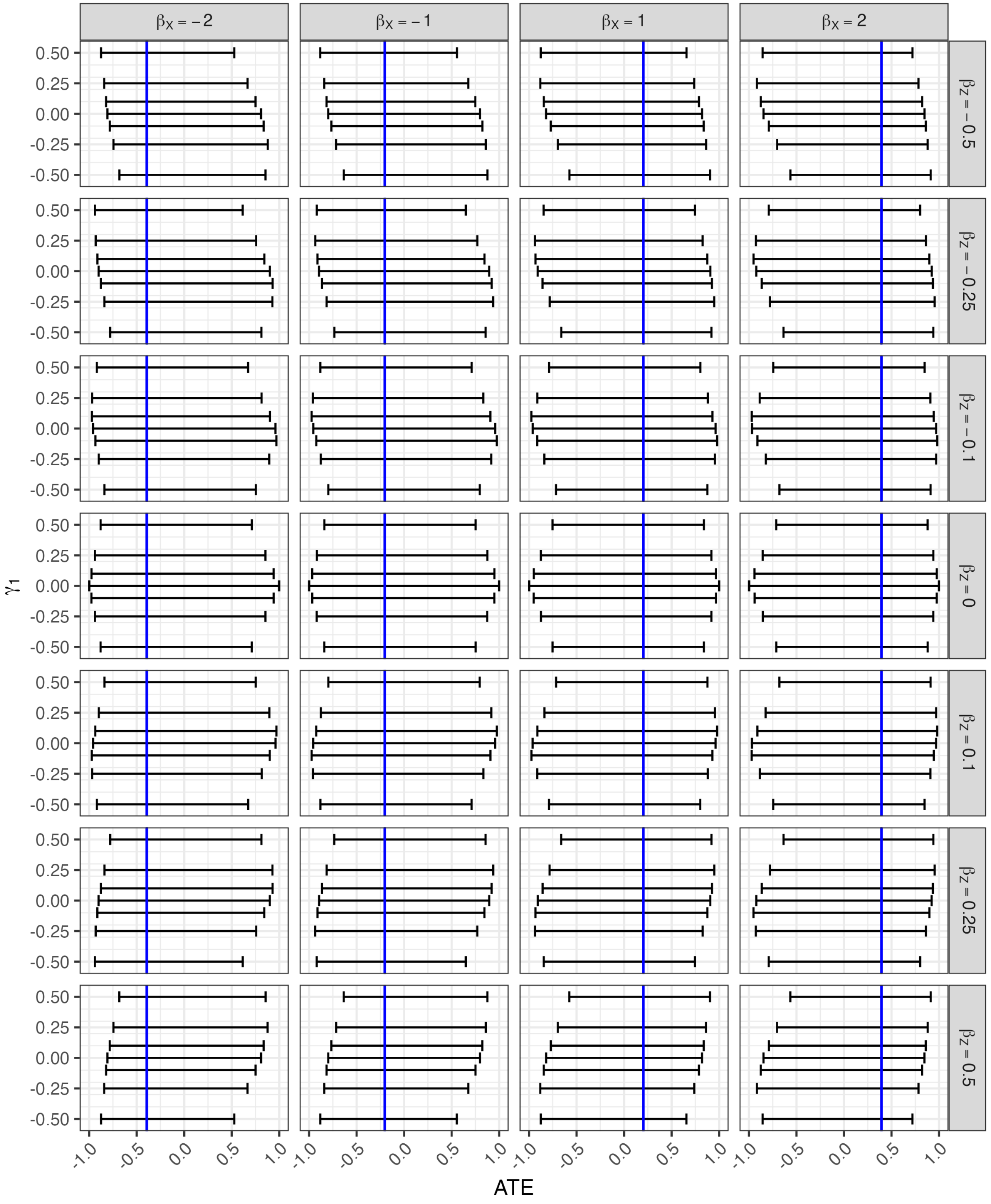

FIGURE G7 Two-sample bounds (horizontal lines) and average treatment effects (vertical blue lines) under pleiotropy. Columns represent effect size of the exposure on the logit scale, rows represent the magnitude of the violation of assumption (A3). X-axis show average treatment effect, and Y-axis represent instrument strength.

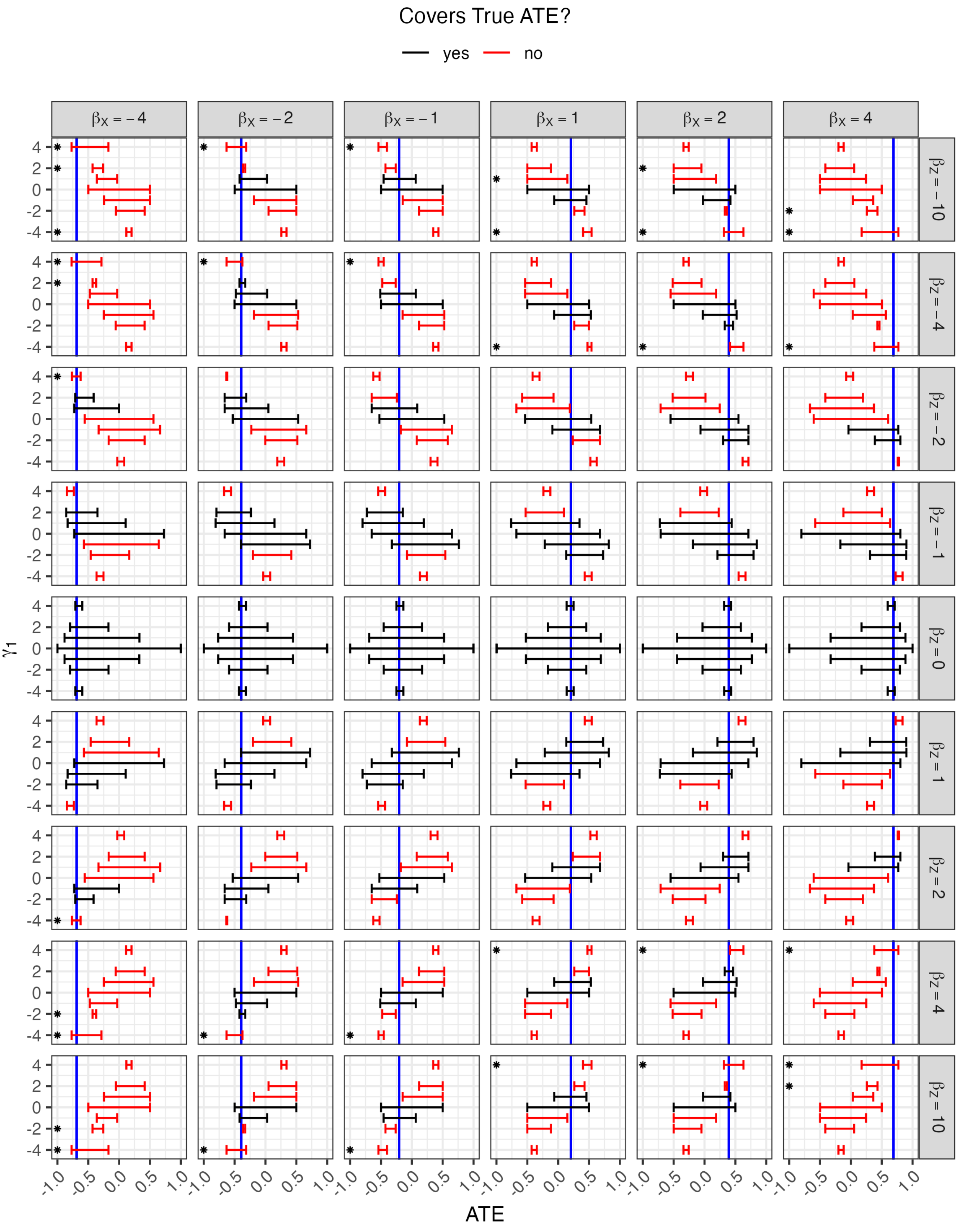

FIGURE G8 Two-sample bounds (horizontal lines) and average treatment effects (vertical blue lines) under pleiotropy. Columns represent effect size of the exposure on the logit scale, rows represent the magnitude of the violation of assumption (A3). X-axis show average treatment effect, and Y-axis represent instrument strength. Cases where any of the verifiable constraints were violated are denoted by an asterix to the left.

H DICHOTOMIZED EXPOSURE VARIABLE

In many MR studies, the exposure and/or the outcome is dichotomization of a continuous variable. For example, in Section 5 we explore the effect of having high cholesterol on the incidence of heart attack. The exposure variable in this example is a dichotomization of the continuous measure of cholesterol.

In this section, we will explore the effect of using a dichotomized version of a continuous variable in nonparametric bounds with a focus on detection of the direction of the causal effect. The main takeaway is that if the analysis using the dichotomized version is able to conclude the direction of the causal effect, the detected direction is the same as the direction of the causal effect of the underlying continuous variable. There are other concerns one should be aware of when using a dichotomization of a continuous variable as a binary exposure[22].

H.1 Theoretical Setup

Let X be a continuous exposure, and Y^x the binary potential outcome. For simplicity, we will work under the monotonicity assumption that $P(Y^x \leq Y^{x+\epsilon}) = 1$ for all x and all $\epsilon > 0$. This assumption simplifies the math, and it is often implicitly made in mendelian randomization studies that rely on regression frameworks. Also note that this assumption gives us the direction of the causal effect of X on Y. In this case, it is positive. Next, let $\tilde{X}$ be a dichotomization of the exposure, that is $\tilde{X} = 1[X \geq c]$ for some c.

Now, we define the potential outcomes under the dichotomized exposure as being one of the potential outcomes under the continuous exposure. I.e. $\tilde{Y}^1 = Y^x$ for some $x \geq c$, and $\tilde{Y}^0 = Y^x$ for some $x < c$.

If we consider the average treatment effect under the dichotomized exposure, we see that

$$\begin{aligned} E[\tilde{Y}^1 - \tilde{Y}^0] &= \sum_{k \in \{-1,0,1\}} k \cdot P(\tilde{Y}^1 - \tilde{Y}^0 = k) \\ &= P(\tilde{Y}^1 - \tilde{Y}^0 = 1) - P(\tilde{Y}^1 - \tilde{Y}^0 = -1) \end{aligned}$$

Note that if $\tilde{Y}^1 - \tilde{Y}^0 = -1$, then $\tilde{Y}^1 < \tilde{Y}^0$. Since $\tilde{Y}^1 = Y^{x'}$ for some $x' \geq c$, and $\tilde{Y}^0 = Y^{x''}$ for some $x'' < c$,

$$P(\tilde{Y}^1 < \tilde{Y}^0) = P(Y^{x'} < Y^{x''}) = 0$$

since $P(Y^x \leq Y^{x+\epsilon}) = 1$ for all x, and all $\epsilon > 0$. Therefore, $P(\tilde{Y}^1 - \tilde{Y}^0 = -1) = 0$, which leads us to conclude that

$$E[\tilde{Y}^1 - \tilde{Y}^0] = P(\tilde{Y}^1 - \tilde{Y}^0 = 1) \geq 0$$

and therefore the sign of the causal effect is preserved.

Similar calculations show that if we instead assume $P(Y^x \geq Y^{x+\epsilon}) = 1$, then

$$E[\tilde{Y}^1 - \tilde{Y}^0] = -P(\tilde{Y}^1 - \tilde{Y}^0 = -1) \leq 0$$

H.2 Simulation Results

To investigate what behavior we can expect in our logistic regression framework, we carried out a simulation study. We use a very similar framework as what is introduced in Section 2.2. In particular, we let the exposure follow

$$\tilde{X} = \gamma_0 + \gamma_1 Z + U + \epsilon_{\tilde{X}},$$

where Z is the instrument taking values in $\{0, 1, 2\}$, U is a binary unmeasured confounder, and $\epsilon_{\tilde{X}}$ is the error term following the standard normal distribution. The outcome follows the logistic model

$$\text{logit}(P(Y = 1 | \tilde{X} = x, U = u)) = \beta_0 + \beta_{\tilde{X}} x + u + \epsilon_Y.$$

where ϵ_Y is the error term following the standard normal distribution.

Based on these two models, we simulate $10,000,000$ observations for each combination of parameters given in Table H9 based on which we find $P(Y = 1 | Z = z)$, and $P(X = 1 | Z = z)$, where $X = 1[\tilde{X} < M_{\tilde{X}}]$, where $M_{\tilde{X}}$ is the median of $\tilde{X}$.

The resulting bounds can be found in Figures H9-H13. We notice that when we are able to detect a direction of the ATE, it is consistently the correct direction.

TABLE H9 Parameters used in simulation to explore the behavior of the bounds when using an exposure that is a dichotomization of an underlying continuous variable.

γ_0	γ_1	β_0	$\beta_{\tilde{X}}$
-2, -1, 0, 1, 2	-2, -1, 0, 1, 2	-2, -1, 0, 1, 2	-2, -1, 0, 1, 2

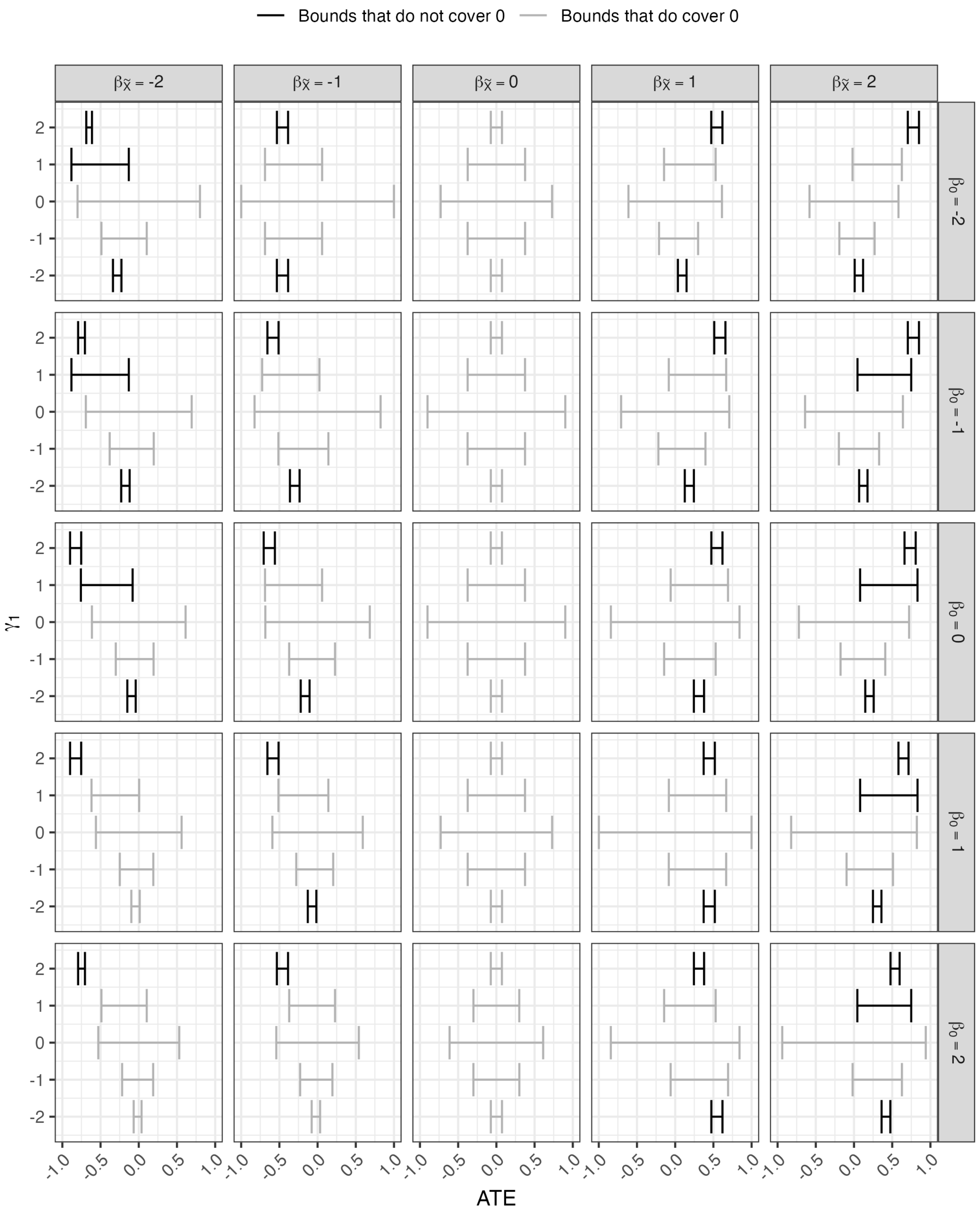

FIGURE H9 Nonparametric bounds based on a dichotomized exposure; here $\gamma_0 = -2$. Columns represent the effect size of the exposure on the logit scale. Rows represent different values of the intercept β_0. The y-axis shows the effect of the instrument on the continuous exposure, and the x-axis shows the average treatment effect.

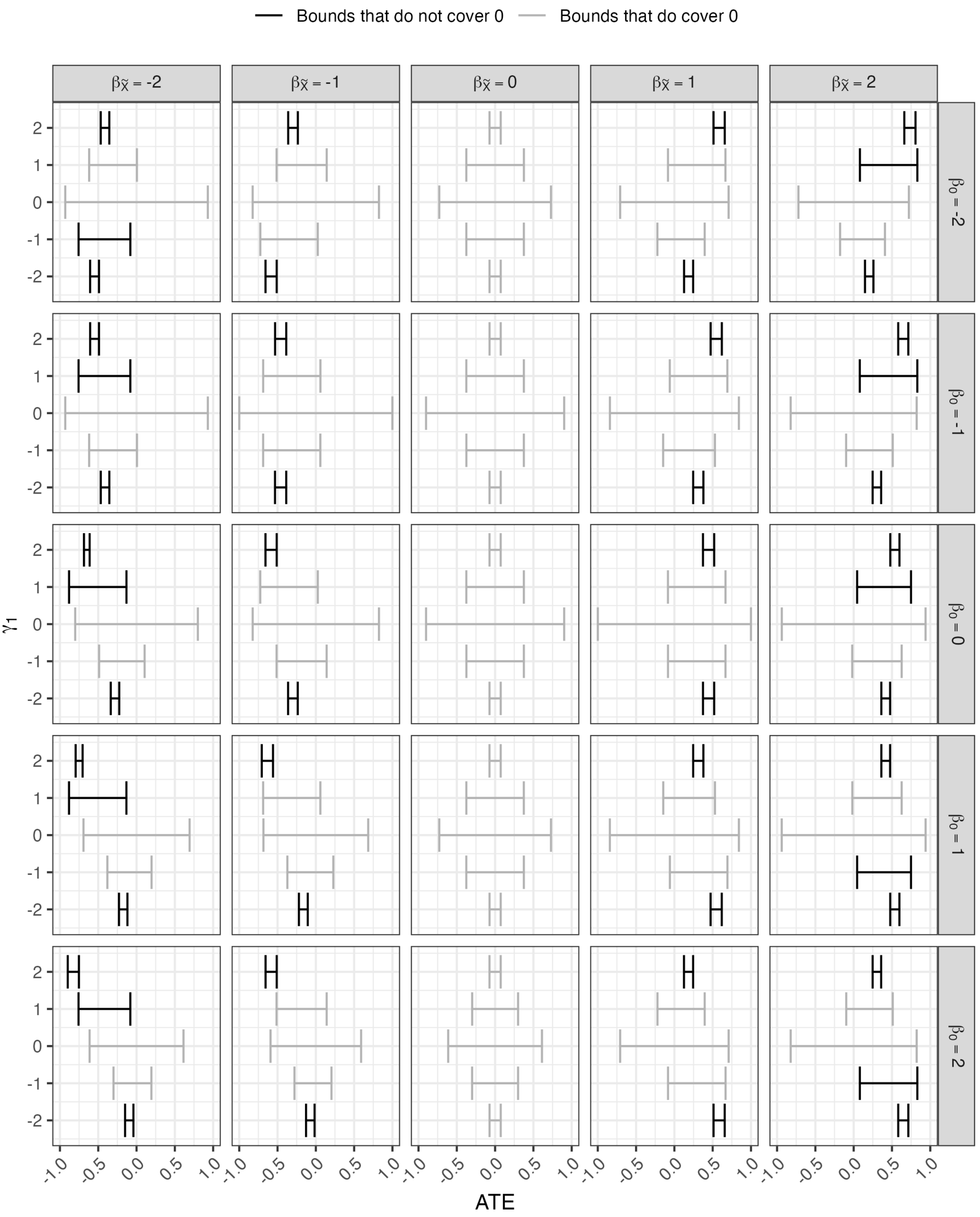

FIGURE H10 Nonparametric bounds based on a dichotomized exposure; here $\gamma_0 = -1$. Columns represent the effect size of the exposure on the logit scale. Rows represent different values of the intercept β_0. The y-axis shows the effect of the instrument on the continuous exposure, and the x-axis shows the average treatment effect.

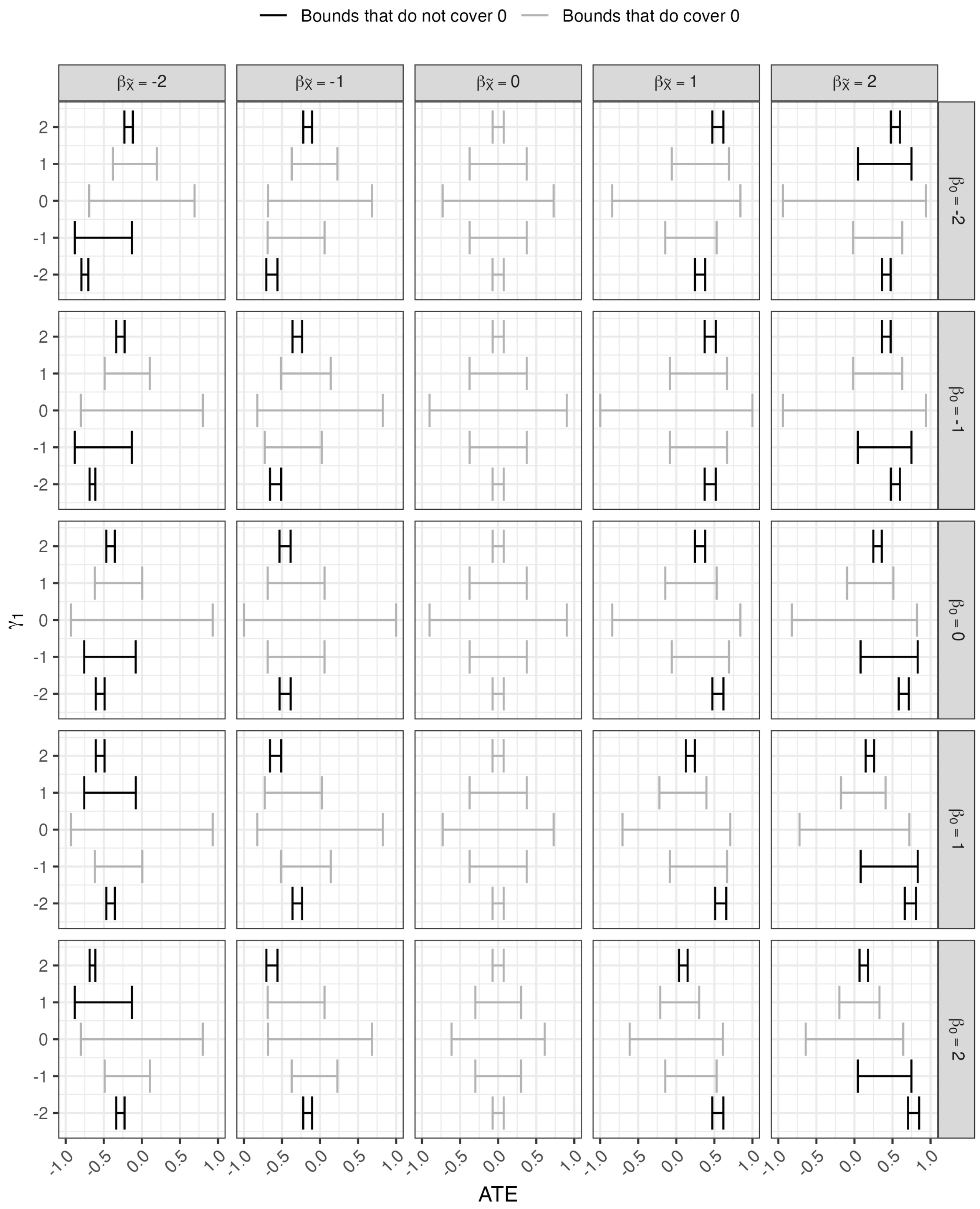

FIGURE H11 Nonparametric bounds based on a dichotomized exposure; here $\gamma_0 = 0$. Columns represent the effect size of the exposure on the logit scale. Rows represent different values of the intercept β_0. The y-axis shows the effect of the instrument on the continuous exposure, and the x-axis shows the average treatment effect.

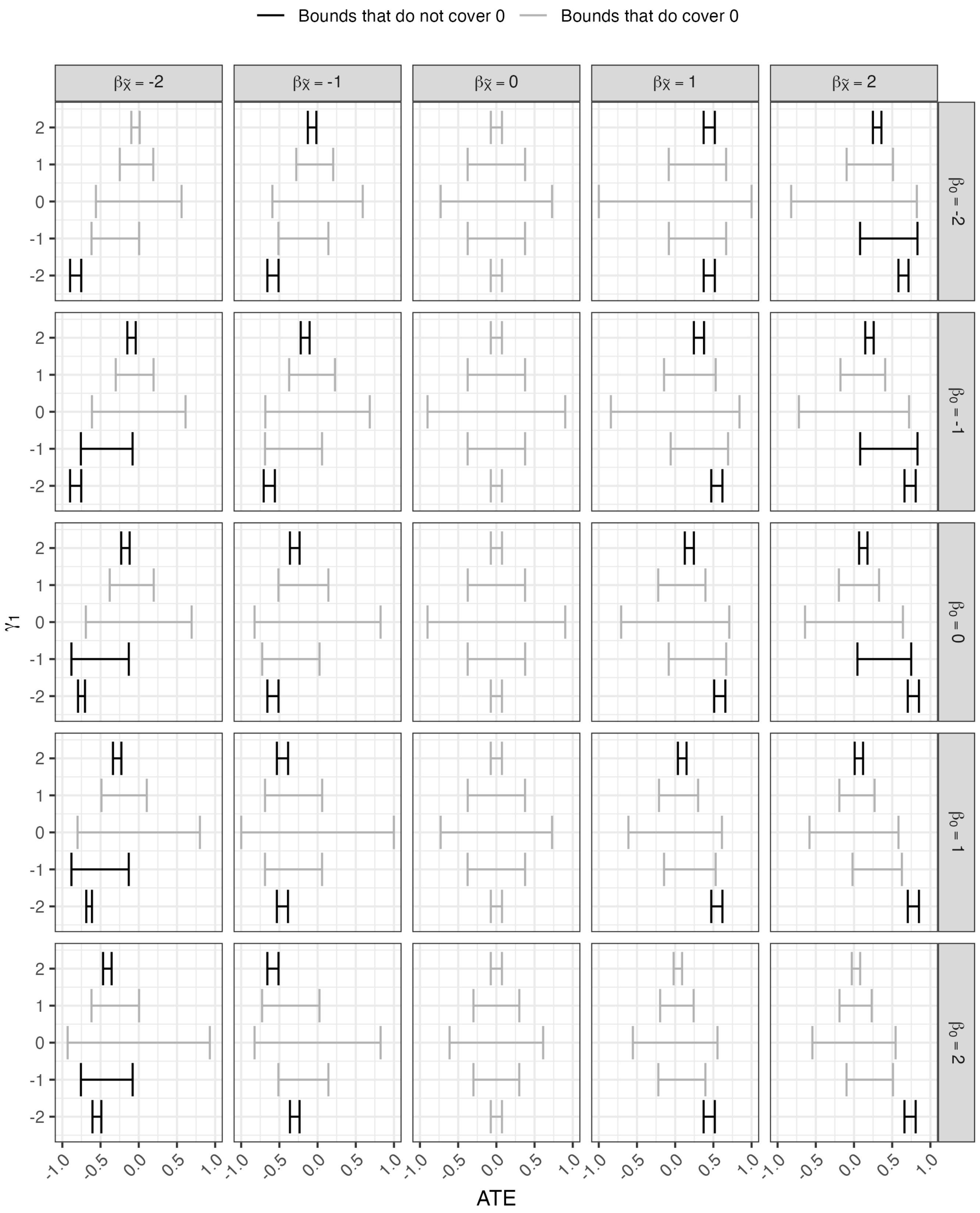

FIGURE H12 Nonparametric bounds based on a dichotomized exposure; here $\gamma_0 = 1$. Columns represent the effect size of the exposure on the logit scale. Rows represent different values of the intercept β_0. The y-axis shows the effect of the instrument on the continuous exposure, and the x-axis shows the average treatment effect.

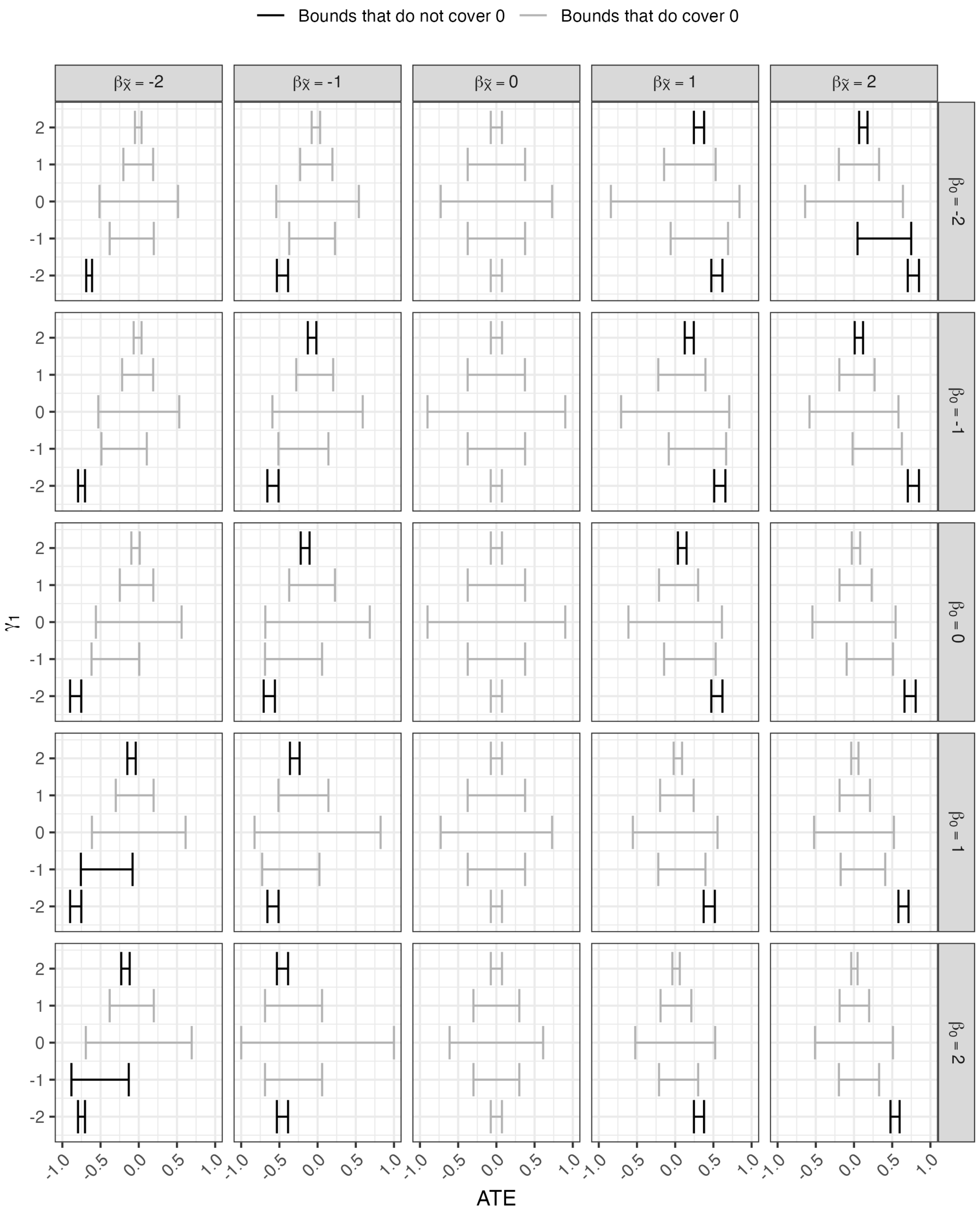

FIGURE H13 Nonparametric bounds based on a dichotomized exposure; here $\gamma_0 = 2$. Columns represent the effect size of the exposure on the logit scale. Rows represent different values of the intercept β_0. The y-axis shows the effect of the instrument on the continuous exposure, and the x-axis shows the average treatment effect.

I FINITE-SAMPLE BEHAVIOR OF BOUNDS WITH ESTIMATED PROBABILITIES

We use the same exposure and outcome models as presented in Section 2.3. In particular, we let the exposure X be binary with

$$\text{logit}(P(X = 1|Z = z, U = u)) = \gamma_0 + \gamma_1 \cdot z + u$$

and the outcome Y be binary with

$$\text{logit}(P(Y = 1|X = x, U = u)) = \beta_0 + \beta_X \cdot x + u.$$

We vary $\gamma_1 \in \{-4, -2, -1 - 0.5, -0.25, -0.1, 0, 0.1, 0.25, 0.5, 1, 2, 4\}$ and $\beta_X \in \{-4, -2, -1, 1, 2, 4\}$, while we set $\gamma_0 = -\gamma_1$, $\beta_0 = -\beta_X/2$ and let U follow a standard Normal. We then generate 5000 data sets with 2000 observations in each. The resulting coverages are presented in Tables I10 and I11. We see that coverage is essentially 100% unless the coefficients are very extreme. In particular, for any values that we might realistically encounter in a two-sample MR study, we would expect 100% coverage.

TABLE I10 Coverage of bounds with sample size 2000 based on 5000 simulated data sets.

γ_1	β_X	ATE	ST	Coverage
-0.5	-2	-0.393	0.205	1
	-1	-0.204	0.204	1
	1	0.204	0.204	1
	2	0.394	0.204	1
-0.25	-2	-0.393	0.103	1
	-1	-0.204	0.103	1
	1	0.204	0.103	1
	2	0.393	0.104	1
-0.1	-2	-0.393	0.041	1
	-1	-0.204	0.042	1
	1	0.204	0.041	1
	2	0.393	0.042	1
0	-2	-0.393	0.001	1
	-1	-0.204	0.001	1
	1	0.204	0.001	1
	2	0.393	0.001	1
0.1	-2	-0.393	0.042	1
	-1	-0.204	0.041	1
	1	0.204	0.041	1
	2	0.393	0.041	1
0.25	-2	-0.393	0.103	1
	-1	-0.204	0.103	1
	1	0.204	0.103	1
	2	0.393	0.103	1
0.5	-2	-0.393	0.204	1
	-1	-0.204	0.204	1
	1	0.204	0.204	1
	2	0.393	0.204	1

TABLE I11 Coverage of bounds with sample size 2000 based on 5000 simulated data sets.

γ_1	β_X	ATE	ST	Coverage
-4	-4	-0.689	0.944	0.8086
	-2	-0.393	0.944	0.8862
	-1	-0.204	0.944	0.8952
	1	0.204	0.944	0.9056
	2	0.394	0.944	0.8682
	4	0.689	0.944	0.8236
-2	-4	-0.689	0.689	1.0000
	-2	-0.393	0.689	1.0000
	-1	-0.204	0.689	1.0000
	1	0.204	0.689	1.0000
	2	0.394	0.689	1.0000
	4	0.689	0.689	1.0000
-1	-4	-0.689	0.394	1.0000
	-2	-0.393	0.394	1.0000
	-1	-0.204	0.393	1.0000
	1	0.204	0.394	1.0000
	2	0.393	0.393	1.0000
	4	0.689	0.394	1.0000
1	-4	-0.689	0.393	1.0000
	-2	-0.393	0.393	1.0000
	-1	-0.204	0.393	1.0000
	1	0.204	0.393	1.0000
	2	0.393	0.394	1.0000
	4	0.689	0.394	1.0000
2	-4	-0.689	0.690	1.0000
	-2	-0.393	0.689	1.0000
	-1	-0.204	0.689	1.0000
	1	0.204	0.689	1.0000
	2	0.393	0.690	1.0000
	4	0.689	0.689	1.0000
4	-4	-0.689	0.944	0.8086
	-2	-0.393	0.944	0.8798
	-1	-0.204	0.944	0.8992
	1	0.204	0.944	0.8952
	2	0.394	0.944	0.8758
	4	0.689	0.944	0.8188

J SAMPLING OF INTERSECTION BOUNDS FROM TWO INSTRUMENTS

To extend our method for sampling plausible joint distributions of $P(X = x, Y = y|Z = z)$ to the scenario where we have multiple instruments available, we simply repeat the one instrument sampling for each instrument. This is equivalent to assuming that the covariances of X and Y given Z_1 are independent of the covariances of X and Y given Z_2. Once we have obtained bounds for each instrument, we take the intersection to get the intersection bounds.

Specifically, say we get bounds $(LB_{1i}, UB_{1i}), i = 1, 2, ..., m$ by sampling m trivariate distributions based on the information we have on (X, Z_1) and (Y, Z_1), and bounds $(LB_{2i}, UB_{2i}), i = 1, 2, ..., m$ by sampling m trivariate distributions based on the information we have on (X, Z_2) and (Y, Z_2). We then create the intersection bounds as $\left(\max_{z\in 1,2} LB_{zi}, \min_{z\in 1,2} UB_{zi}\right), i = 1, 2, ..., m$. This, under the assumption that $\mathrm{Cov}(X, Y|Z_1 = z)$ and $\mathrm{Cov}(X, Y|Z_2 = z)$ are independent of each other, gives us a sample from the posterior distribution of intersection bounds. We can use this to assess the potential usefulness of aggregating information from two sets of trivariate data, (X, Y, Z_1) and (X, Y, Z_2), using intersection bounds.

K ADDITIONAL SUMMARY STATISTICS AND FIGURES FOR ANALYSES

We present expanded results to complement the analyses in Section 5.

We use the `TwoSampleMR` R package[45] to extract and preprocess the data for our analyses. For preprocessing, we followed the defaults of the R pacakge where linkage disequilibrium based clumping ($r^2 \geq 0.001$ within a $10,000$ kb window using $p < 5 \times 10^{-8}$ as the level of significance) were performed such that only independent instruments with significant associations were used in the analysis. Afterwards, we obtain the estimated coefficients corresponding to the effects of the SNPs on the exposure and the outcome from a logistic model. Since estimates of the intercept are not included in these reported results, but the marginal proportions of the outcome, exposure, and allele frequencies are known, we find the intercepts by solving $P(X = 1) = \sum_{z=0}^{2} \mathrm{logit}(\gamma_0 + \hat{\gamma}_j \cdot z) \cdot P(Z_j = z)$ and $P(Y = 1) = \sum_{z=0}^{2} \mathrm{logit}(\Gamma_0 + \hat{\Gamma}_j \cdot z) \cdot P(Z_j = z)$ for Γ_0 and Γ_0, respectively. This allows us to obtain estimates of $P(Y = 1|Z_j = z)$ and $P(X = 1|Z_j = z)$ for every j and $z = 0, 1, 2$.

Data on smoking was obtained from the data entry ID ukb-d-20116_0, data on lung cancer was from data entry ID ukb-d-40001_C349, data on cholesterol was from data entry ID ukb-a-108, and data on heart attack was from data entry ID ukb-a-434.

K.1 Effect of Smoking on Lung Cancer

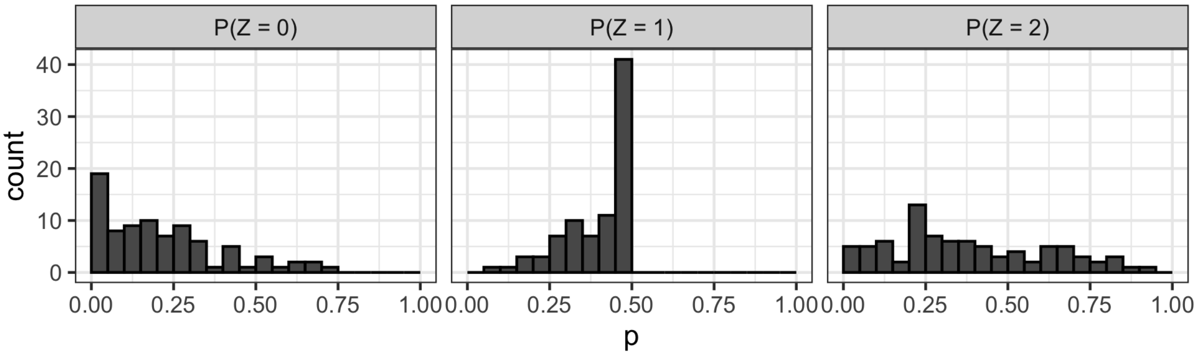

FIGURE K14 Histograms of the marginal distribution of instruments, $P(Z = z), z = 0, 1, 2$, estimated after preprocessing.

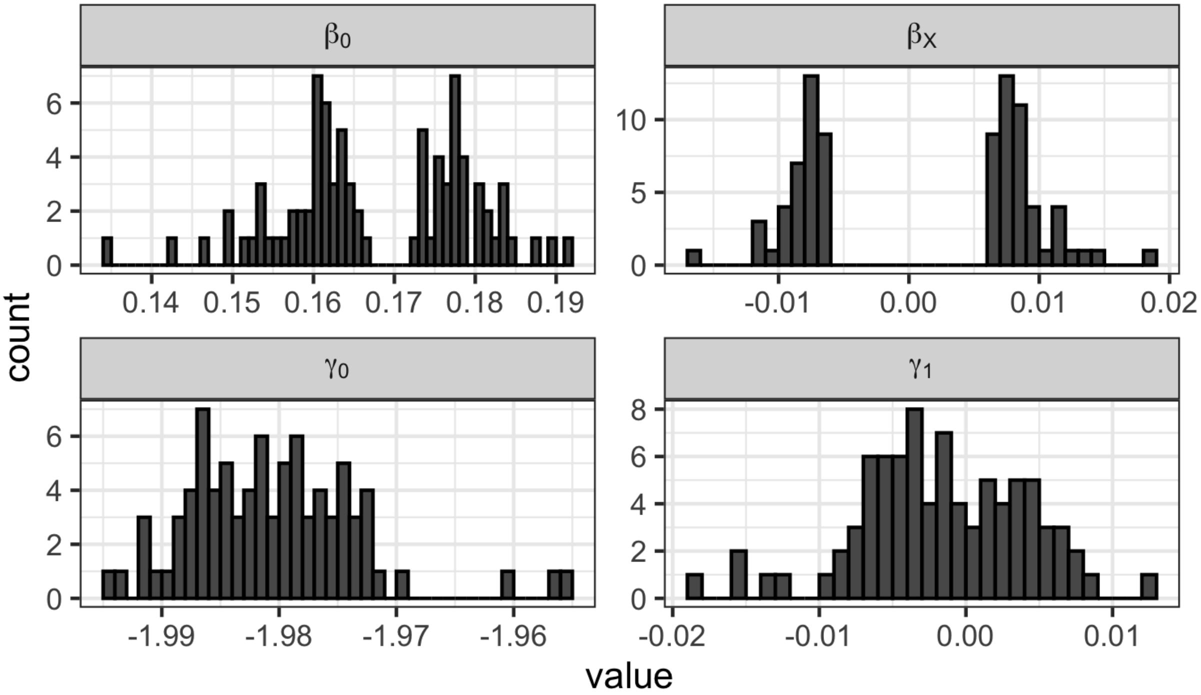

FIGURE K15 Histograms of the coefficients from GWAS results of logistic regression of the SNPs on smoking status and lung cancer status. Intercepts (β_0 and γ_0) are inferred, while slopes (β_X and γ_1) are as reported.

TABLE K13 Coefficients from GWAS results of logistic regression of the SNPs on smoking status and lung cancer status. Intercepts (β_0 and γ_0) are inferred, while slopes (β_X and γ_1) are as reported.

SNP	β_X	β_0	γ_1	γ_0
rs10173733	-0.0065148	0.1773766	0.0033363	-1.987122
rs10193706	-0.0117667	0.1807753	-0.0015310	-1.981684
rs10233018	-0.0076551	0.1771914	0.0050495	-1.988150
rs10274594	0.0078326	0.1617046	-0.0015364	-1.981589
rs1029986	-0.0070208	0.1754303	0.0035498	-1.986088
rs10774625	0.0074868	0.1621777	-0.0084158	-1.974806
rs10813628	-0.0068761	0.1762662	0.0051706	-1.988156
rs10897561	-0.0066917	0.1782117	0.0066835	-1.991747
rs10905461	0.0072731	0.1658787	-0.0058844	-1.980131
rs10914684	0.0077356	0.1591408	-0.0026047	-1.979616
rs10956808	0.0076247	0.1607905	-0.0063546	-1.975802
rs11103667	-0.0086047	0.1835048	0.0063118	-1.993343
rs11127913	0.0081801	0.1596256	-0.0033969	-1.978997
rs11429972	0.0083148	0.1640148	-0.0096129	-1.976695
rs11611651	-0.0119868	0.1914724	0.0013059	-1.985521
rs11631530	-0.0099863	0.1872160	-0.0047887	-1.974691
rs11646575	-0.0082446	0.1788545	0.0012319	-1.984521
rs11693702	-0.0080254	0.1781679	0.0046224	-1.988077
rs117435980	-0.0092037	0.1849986	-0.0054804	-1.973970
rs12042107	0.0071759	0.1631404	-0.0020557	-1.981288

TABLE K13 Coefficients from GWAS results of logistic regression of the SNPs on smoking status and lung cancer status. Intercepts (β_0 and γ_0) are inferred, while slopes (β_X and γ_1) are as reported. *(continued)*

SNP	β_X	β_0	γ_1	γ_0
rs12244388	-0.0104344	0.1834505	0.0019355	-1.985707
rs12450028	-0.0070626	0.1788556	-0.0024536	-1.979923
rs12479064	-0.0080362	0.1823251	-0.0088600	-1.969116
rs12487411	0.0075048	0.1616745	-0.0077980	-1.974913
rs12608052	0.0067542	0.1631129	-0.0048100	-1.978521
rs12725407	0.0081386	0.1564297	-0.0067998	-1.972138
rs12886628	-0.0071010	0.1743626	-0.0018595	-1.981891
rs12910916	-0.0090138	0.1838027	0.0026458	-1.987308
rs13100688	0.0072663	0.1604864	-0.0055464	-1.976186
rs1492546	-0.0068801	0.1757890	0.0040638	-1.986797
rs1499982	-0.0114648	0.1730098	0.0024892	-1.983878
rs1549213	0.0085270	0.1634849	0.0056335	-1.987184
rs1561195	-0.0078947	0.1771393	0.0072232	-1.990046
rs1565735	0.0115901	0.1510915	-0.0072487	-1.971566
rs16951001	-0.0066035	0.1772784	0.0070226	-1.991313
rs17003752	0.0098606	0.1526117	-0.0055424	-1.973591
rs17151637	0.0075112	0.1588020	-0.0027771	-1.979146
rs1899896	-0.0079928	0.1808293	0.0047935	-1.989876
rs2240294	0.0069566	0.1618616	-0.0078381	-1.974429
rs2416770	-0.0064888	0.1756858	-0.0035668	-1.979794
rs264974	0.0093111	0.1600323	-0.0047198	-1.978291
rs2675609	0.0081586	0.1635228	-0.0069708	-1.977953
rs2797116	0.0079136	0.1580011	-0.0039635	-1.977330
rs2867749	0.0069446	0.1601396	-0.0032894	-1.978658
rs299688	-0.0072721	0.1737306	-0.0019058	-1.982055
rs326341	0.0065809	0.1627032	0.0031753	-1.986468
rs35891966	0.0147752	0.1421811	-0.0122161	-1.960473
rs379525	-0.0064906	0.1763327	-0.0018594	-1.981209
rs42417	-0.0070331	0.1739582	0.0003829	-1.983375
rs4566215	0.0066219	0.1634100	-0.0035546	-1.979817
rs4910656	0.0068438	0.1605890	-0.0006962	-1.982221
rs4957528	-0.0084750	0.1731252	0.0036288	-1.984649
rs523528	0.0080708	0.1629116	0.0029251	-1.985564
rs528301	-0.0086008	0.1773068	0.0124616	-1.994333
rs55921136	0.0085950	0.1559000	-0.0069653	-1.972040
rs568599	-0.0067027	0.1757286	0.0043346	-1.987105
rs5850689	0.0119733	0.1608296	-0.0038879	-1.980291
rs60745548	0.0071946	0.1656670	0.0062353	-1.986552
rs6141314	-0.0080616	0.1818108	0.0010534	-1.984733
rs6265	0.0101598	0.1531146	-0.0043806	-1.976031
rs6433897	-0.0072353	0.1734104	-0.0011588	-1.982527
rs6676022	0.0115926	0.1492373	-0.0153059	-1.956268
rs6690680	0.0088409	0.1547067	-0.0050219	-1.974679

TABLE K13 Coefficients from GWAS results of logistic regression of the SNPs on smoking status and lung cancer status. Intercepts (β_0 and γ_0) are inferred, while slopes (β_X and γ_1) are as reported. *(continued)*

SNP	β_X	β_0	γ_1	γ_0
rs6828849	0.0067122	0.1617773	0.0008050	-1.984076
rs71550128	-0.0073950	0.1762278	0.0034139	-1.986200
rs72505558	0.0067437	0.1614885	-0.0009876	-1.981950
rs72678864	0.0097538	0.1534836	-0.0034394	-1.977455
rs7333559	0.0080523	0.1662222	-0.0183846	-1.975467
rs7451586	-0.0066732	0.1775422	0.0027432	-1.986404
rs748828	0.0086213	0.1572389	-0.0047229	-1.976368
rs7528604	0.0068658	0.1618157	-0.0001820	-1.982931
rs7567570	-0.0091324	0.1727617	-0.0002451	-1.983053
rs763053	0.0080618	0.1570972	-0.0069210	-1.972409
rs76608582	0.0182891	0.1347646	-0.0048192	-1.973958
rs772921	0.0072725	0.1600453	-0.0054837	-1.975937
rs77878475	0.0125950	0.1465726	0.0010985	-1.985146
rs7870475	-0.0071900	0.1771594	0.0082598	-1.991835
rs7948789	-0.0161713	0.1894568	0.0009336	-1.984284
rs883403	0.0094240	0.1536556	-0.0014726	-1.980646
rs9375371	-0.0073963	0.1804155	-0.0069852	-1.972929
rs9381917	0.0112569	0.1493838	-0.0155636	-1.955201
rs9423279	0.0076695	0.1643324	0.0046716	-1.986350
rs9487626	0.0131029	0.1648247	-0.0136868	-1.978168
rs9835772	-0.0078024	0.1814198	-0.0031275	-1.978401

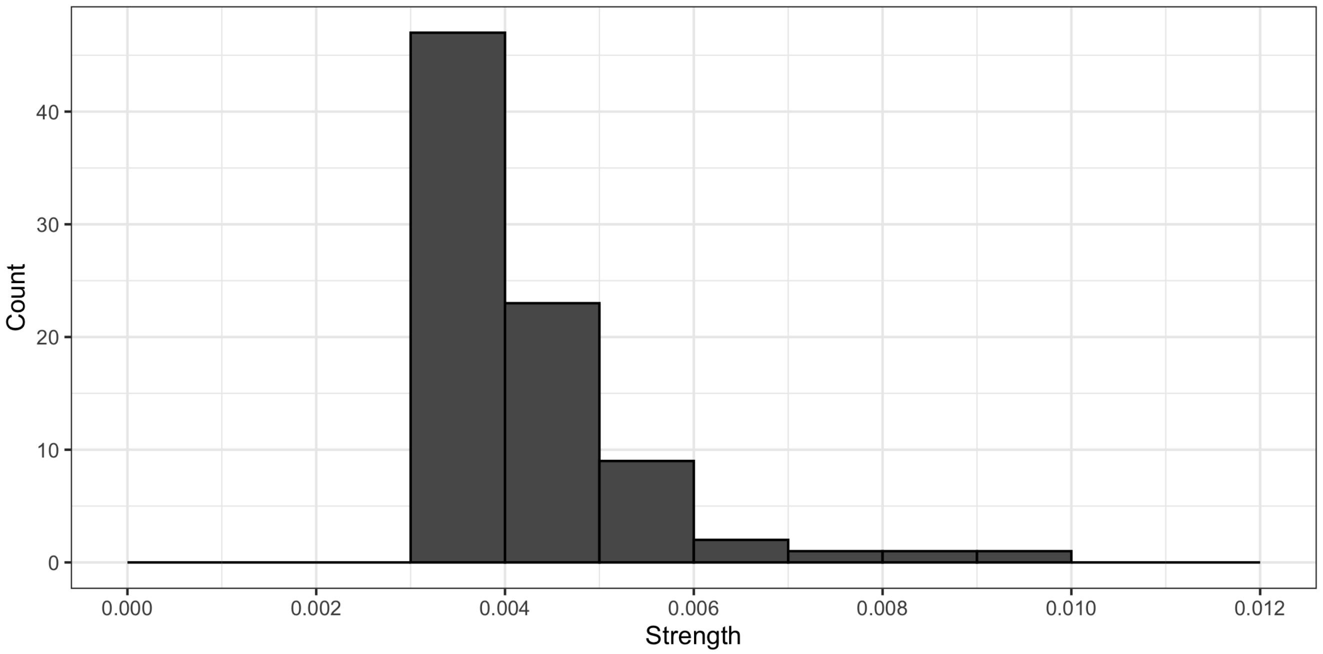

FIGURE K16 Histogram of strengths of IVs on the exposure. Here, SNPs are IVs, and smoking status (ever/never) is exposure. We see that all IVs are very weak, with the largest value just below 0.01.

TABLE K12 Table of the marginal distribution of instruments, $P(Z = z)$, $z = 0, 1, 2$, estimated after preprocessing for analysis.

SNP	P(Z = 2)	P(Z = 1)	P(Z = 0)
rs10173733	0.3562119	0.4812460	0.1625421
rs10193706	0.2254196	0.4987283	0.2758521
rs10233018	0.2458307	0.4999649	0.2542044
rs10274594	0.2540510	0.4999674	0.2459816
rs1029986	0.1723980	0.4856208	0.3419813
rs10774625	0.2457332	0.4999633	0.2543035
rs10813628	0.2349574	0.4995333	0.2655093
rs10897561	0.4140371	0.4588401	0.1271228
rs10905461	0.0654474	0.3807590	0.5537936
rs10914684	0.4570550	0.4380069	0.1049382
rs10956808	0.3337643	0.4879181	0.1783175
rs11103667	0.6528207	0.3103050	0.0368743
rs11127913	0.3717426	0.4759287	0.1523286
rs11429972	0.1128192	0.4461330	0.4410478
rs11611651	0.8323808	0.1599365	0.0076827
rs11631530	0.7779345	0.2081429	0.0139226
rs11646575	0.3149600	0.4925059	0.1925340
rs11693702	0.2849095	0.4977193	0.2173712
rs117435980	0.6998026	0.2734789	0.0267185
rs12042107	0.2025948	0.4950210	0.3023842
rs12244388	0.4404143	0.4464457	0.1131399
rs12450028	0.4293549	0.4517938	0.1188513
rs12479064	0.6268375	0.3297864	0.0433761
rs12487411	0.2788384	0.4984262	0.2227354
rs12608052	0.2306302	0.4992191	0.2701507
rs12725407	0.6546886	0.3088794	0.0364320
rs12886628	0.1124522	0.4457734	0.4417744
rs12910916	0.6206505	0.3343265	0.0450230
rs13100688	0.3932914	0.4676762	0.1390324
rs1492546	0.2022894	0.4949531	0.3027575
rs1499982	0.0221071	0.2531548	0.7247382
rs1549213	0.1285982	0.4600154	0.4113864
rs1561195	0.2279701	0.4989841	0.2730458
rs1565735	0.6376078	0.3217914	0.0406009
rs16951001	0.3380123	0.4867519	0.1752358
rs17003752	0.7420669	0.2387323	0.0192008
rs17151637	0.5166809	0.4042486	0.0790705
rs1899896	0.4934387	0.4180265	0.0885349
rs2240294	0.3093641	0.4936820	0.1969539
rs2416770	0.2199058	0.4980707	0.2820235
rs264974	0.2640248	0.4996173	0.2363579
rs2675609	0.1387352	0.4674731	0.3937917
rs2797116	0.5370791	0.3915554	0.0713655
rs2867749	0.4639468	0.4343792	0.1016740
rs299688	0.0806544	0.4066855	0.5126601
rs326341	0.2745833	0.4988473	0.2265693
rs35891966	0.8609698	0.1338295	0.0052006
rs379525	0.2690001	0.4993042	0.2316957
rs42417	0.0959979	0.4276747	0.4763274
rs4566215	0.2184561	0.4978736	0.2836703
rs4910656	0.4334112	0.4498570	0.1167317
rs4957528	0.0432505	0.3294341	0.6273153
rs523528	0.1717181	0.4853414	0.3429405
rs528301	0.2006916	0.4945891	0.3047192
rs55921136	0.6351822	0.3236020	0.0412158
rs568599	0.2090011	0.4963306	0.2946684
rs5850689	0.1341980	0.4642649	0.4015371
rs60745548	0.0747101	0.3972427	0.5280472
rs6141314	0.5735637	0.3675524	0.0588839
rs6265	0.6582586	0.3061456	0.0355959
rs6433897	0.0693372	0.3879647	0.5426982
rs6676022	0.7713790	0.2138057	0.0148153
rs6690680	0.7094689	0.2656618	0.0248694
rs6828849	0.3395694	0.4863129	0.1741177
rs71550128	0.2008017	0.4946147	0.3045837
rs72505558	0.3617072	0.4794276	0.1588652
rs72678864	0.6825787	0.2872090	0.0302123
rs7333559	0.0439935	0.3315056	0.6245008
rs7451586	0.3541182	0.4819202	0.1639616
rs748828	0.5139770	0.4058898	0.0801332
rs7528604	0.3213716	0.4910497	0.1875787
rs7567570	0.0299625	0.2862686	0.6837689
rs763053	0.6013164	0.3482591	0.0504245
rs76608582	0.9070039	0.0907272	0.0022689
rs772921	0.4315416	0.4507533	0.1177051
rs77878475	0.8356836	0.1569474	0.0073690
rs7870475	0.2763346	0.4986816	0.2249839
rs7948789	0.3767706	0.4740916	0.1491378
rs883403	0.7156415	0.2606289	0.0237296
rs9375371	0.5345687	0.3931467	0.0722846
rs9381917	0.8063218	0.1832649	0.0104133
rs9423279	0.1179428	0.4509704	0.4310869
rs9487626	0.0332246	0.2981030	0.6686724
rs9835772	0.5737177	0.3674477	0.0588346

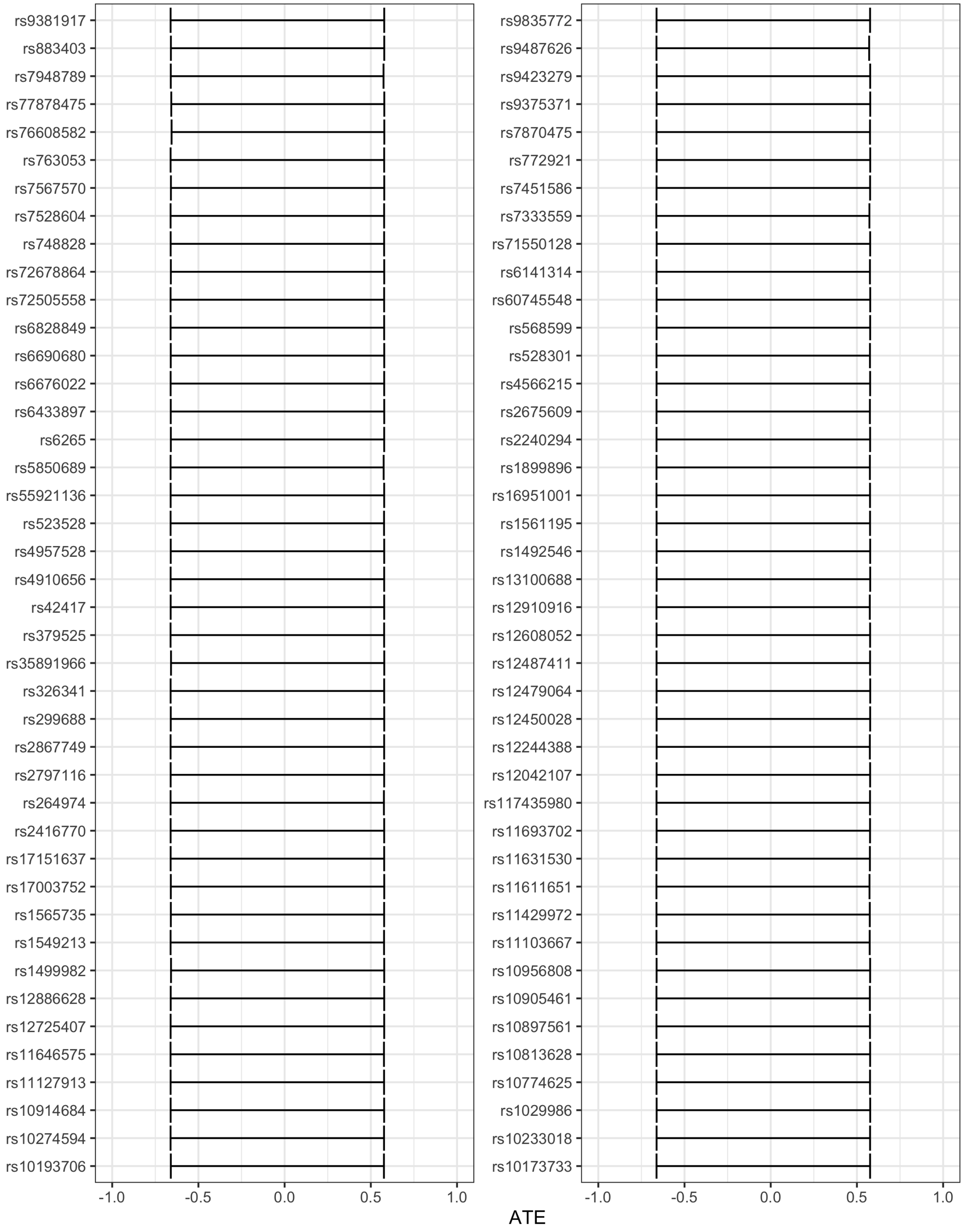

FIGURE K17 Nonparametric two-sample IV bounds on the average treatment effect of smoking on the incidence of lung cancer.

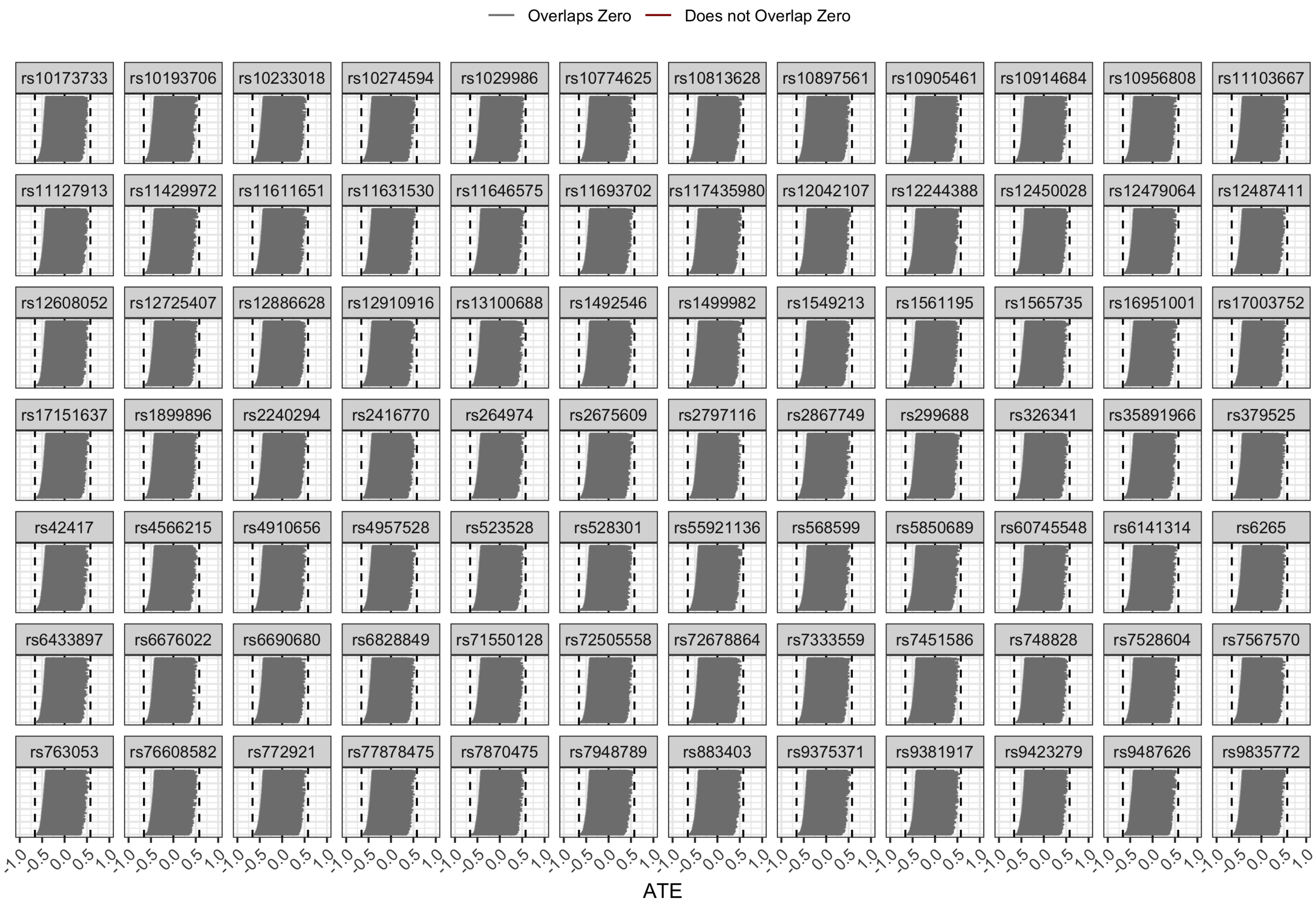

FIGURE K18 500 sets of bounds of the average treatment effect of smoking on lung cancer for each of the 84 SNPs. Each bound is based on a set of values for the trivariate distribution randomly sampled. Bounds are color coded to show if they overlap 0 (grey) or do not (red). All bounds overlap 0.

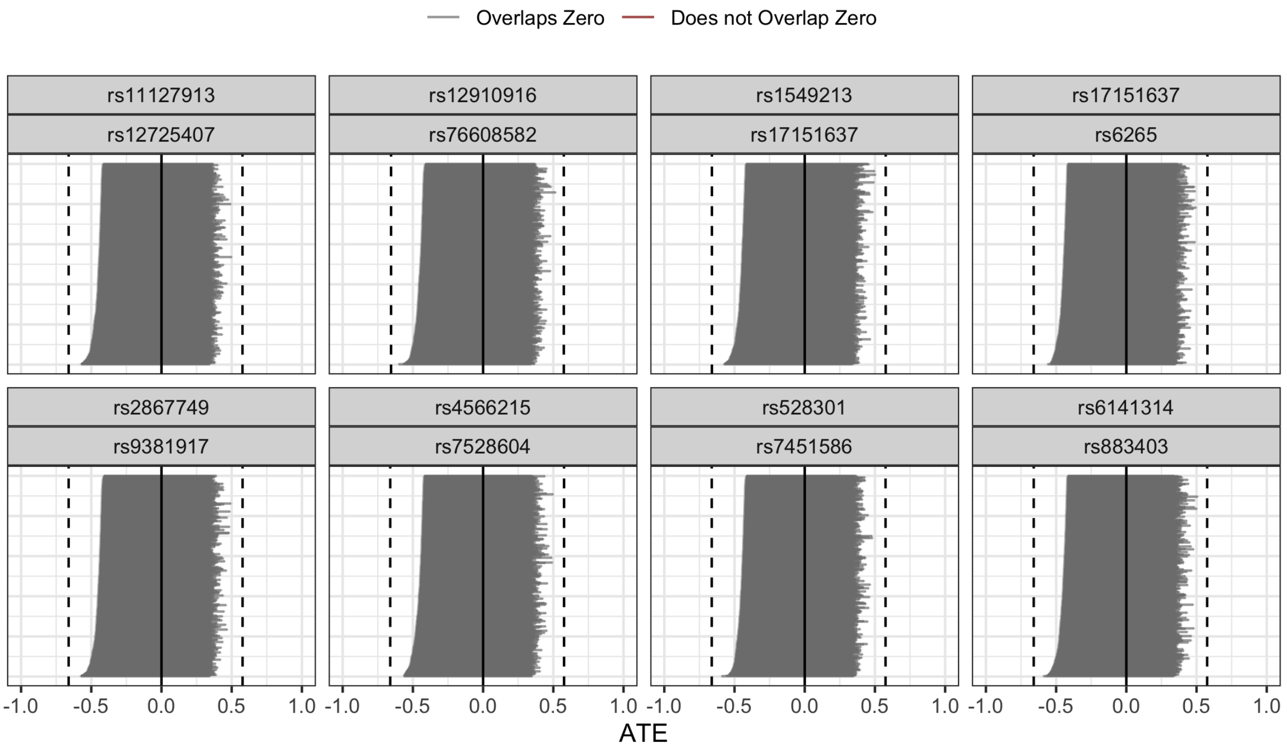

FIGURE K19 Intersection bounds of the average treatment effect of smoking on lung cancer based on randomly sampled trivariate distributions from pairs of SNPs. These 8 pairs were randomly chosen from all possible pairs.

K.2 Effect of High Cholesterol on Heart Attack

TABLE K15 Coefficients from GWAS results of logistic regression of the SNPs on high cholesterol and heart attack status. Intercepts (β_0 and γ_0) are inferred, while slopes (β_X and γ_1) are as reported.

SNP	β_X	β_0	γ_1	γ_0
rs10096633	-0.0089830	-3.727152	-0.0012995	-1.966860
rs10260606	0.0076950	-3.755485	0.0007029	-1.970288
rs10410835	0.0071078	-3.749661	0.0007948	-1.969894
rs10504255	-0.0056764	-3.739063	-0.0000742	-1.969088
rs10804330	-0.0050169	-3.737181	-0.0012539	-1.967709
rs112019714	0.0251675	-3.791824	0.0025525	-1.974100
rs11580878	-0.0051399	-3.737725	-0.0006621	-1.968472
rs11591147	-0.0476105	-3.649365	-0.0054389	-1.958449

TABLE K15 Coefficients from GWAS results of logistic regression of the SNPs on high cholesterol and heart attack status. Intercepts (β_0 and γ_0) are inferred, while slopes (β_X and γ_1) are as reported. *(continued)*

SNP	β_X	β_0	γ_1	γ_0
rs117733303	0.0311528	-3.804047	0.0116909	-1.992088
rs12471811	0.0084776	-3.758037	0.0000048	-1.969147
rs1260326	-0.0102312	-3.734879	-0.0003941	-1.968828
rs12740374	-0.0183231	-3.714419	-0.0025251	-1.965207
rs12916	0.0104793	-3.755479	0.0006700	-1.969941
rs1367117	0.0155585	-3.763513	0.0011495	-1.970658
rs1601935	-0.0061378	-3.738671	-0.0007014	-1.968655
rs1883025	-0.0069826	-3.732469	-0.0013153	-1.967173
rs1883711	0.0241076	-3.789616	0.0026734	-1.974319
rs2125345	-0.0056374	-3.734933	-0.0009408	-1.967809
rs2237107	-0.0070166	-3.731732	-0.0007194	-1.967993
rs2244608	0.0070205	-3.752512	0.0010406	-1.970563
rs2618567	-0.0047485	-3.739660	-0.0007455	-1.968630
rs2738447	0.0081671	-3.749563	0.0016947	-1.970520
rs28601761	-0.0140739	-3.726664	-0.0011169	-1.967847
rs28807203	-0.0106943	-3.722554	-0.0002164	-1.968726
rs3127580	0.0076693	-3.755804	0.0022978	-1.973006
rs34042070	0.0094413	-3.758272	0.0002698	-1.969577
rs34707604	0.0058521	-3.751591	0.0002016	-1.969438
rs3918226	0.0081783	-3.757916	0.0028105	-1.974301
rs4299376	-0.0111342	-3.735719	-0.0012431	-1.968335
rs4470903	0.0067035	-3.753387	0.0014579	-1.971420
rs456598	0.0065720	-3.754166	0.0005768	-1.970127
rs4704727	0.0074887	-3.747988	0.0007432	-1.969643
rs472495	0.0064154	-3.747379	0.0004743	-1.969469
rs56299331	0.0057258	-3.752033	0.0001068	-1.969308
rs57180587	0.0081592	-3.756830	0.0013685	-1.971475
rs58542926	-0.0146353	-3.715853	-0.0013536	-1.966636
rs58691354	0.0074756	-3.755521	0.0000196	-1.969171
rs59950280	0.0058286	-3.750690	0.0004805	-1.969780
rs6090040	-0.0055812	-3.737545	-0.0007168	-1.968450
rs622871	0.0065093	-3.746991	0.0013161	-1.969966
rs635634	0.0098788	-3.758987	0.0014151	-1.971442
rs6458349	0.0056558	-3.746031	0.0007529	-1.969556
rs6511720	-0.0261322	-3.696906	-0.0030216	-1.963813
rs7012637	0.0047984	-3.747932	0.0002456	-1.969396
rs7213086	0.0047773	-3.747169	0.0007846	-1.969840
rs73534263	0.0071810	-3.755717	0.0000767	-1.969275
rs7412	-0.0374088	-3.674234	-0.0038000	-1.962153
rs74617384	0.0190473	-3.777927	0.0069894	-1.981990
rs7534572	0.0081187	-3.748658	0.0005830	-1.969551
rs7707394	0.0061511	-3.750841	0.0000817	-1.969243
rs77542162	0.0253674	-3.792474	0.0020548	-1.973154

TABLE K15 Coefficients from GWAS results of logistic regression of the SNPs on high cholesterol and heart attack status. Intercepts (β_0 and γ_0) are inferred, while slopes (β_X and γ_1) are as reported. *(continued)*

SNP	β_X	β_0	γ_1	γ_0
rs799157	-0.0108031	-3.741956	-0.0003979	-1.969103
rs9376091	-0.0053004	-3.735070	-0.0005561	-1.968317
rs964184	-0.0215630	-3.737246	-0.0013629	-1.968778

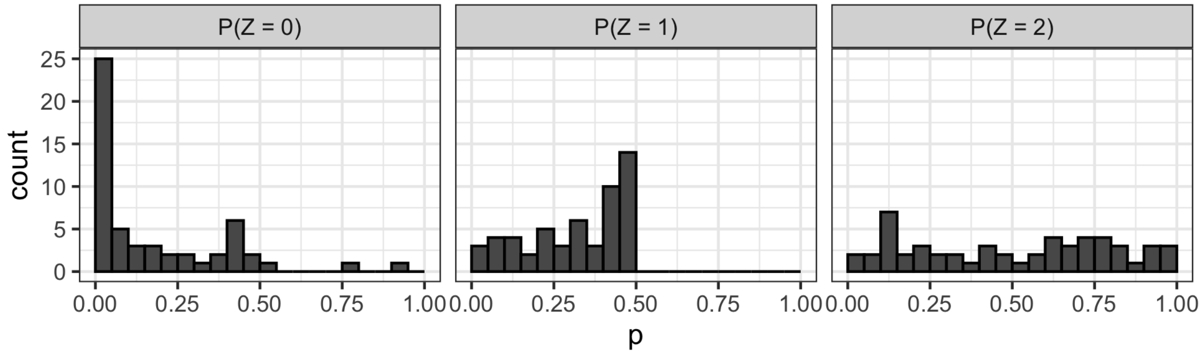

FIGURE K20 Histograms of the marginal distribution of instruments, $P(Z = z), z = 0, 1, 2$, estimated after preprocessing.

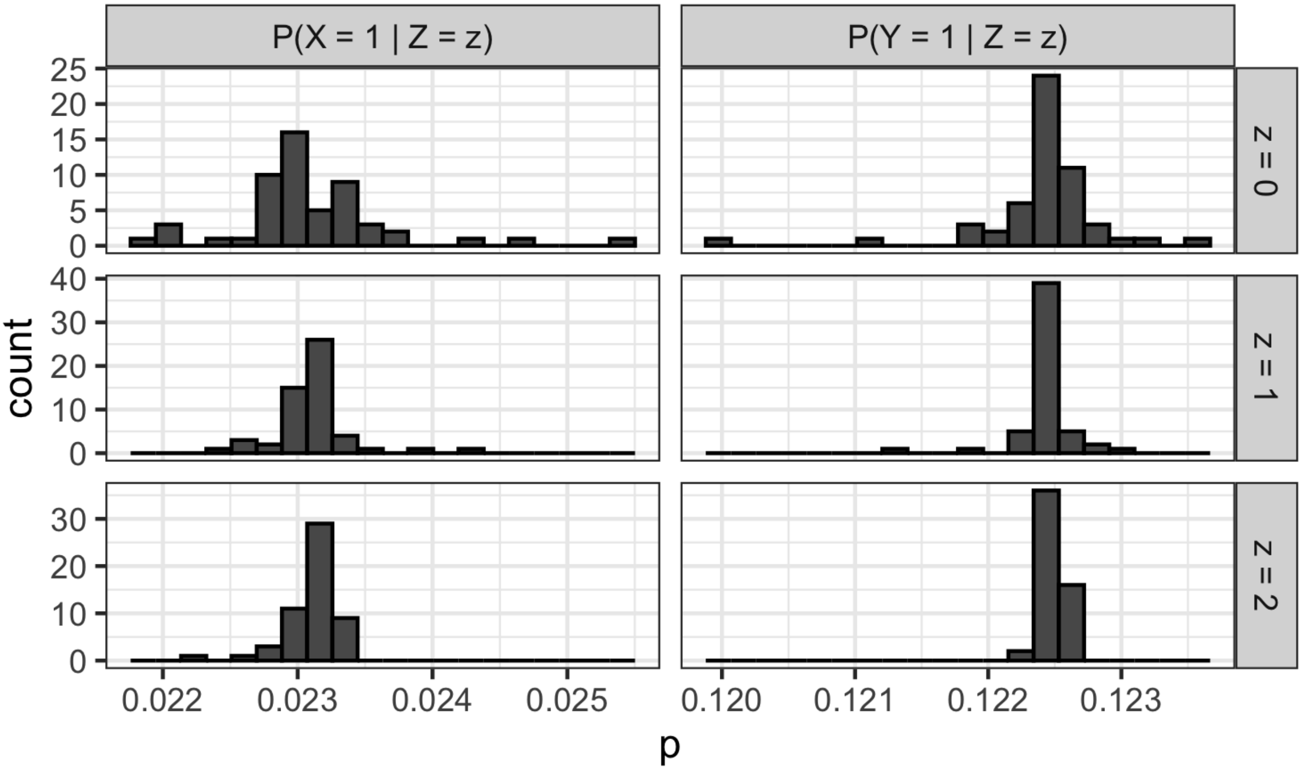

FIGURE K21 Histograms of the marginal conditional probabilities $P(X = 1|Z = z), z = 0, 1, 2$ and $P(Y = 1|Z = z), z = 0, 1, 2$.

TABLE K14 Table of the marginal distribution of instruments, $P(Z = z)$, $z = 0, 1, 2$, estimated after preprocessing for analysis.

SNP	P(Z = 2)	P(Z = 1)	P(Z = 0)
rs10096633	0.7682873	0.2164654	0.0152473
rs10260606	0.6689457	0.2978906	0.0331637
rs10410835	0.2261041	0.4987999	0.2750961
rs10504255	0.1141345	0.4474070	0.4384585
rs10804330	0.3246447	0.4902626	0.1850927
rs112019714	0.9445278	0.0546808	0.0007914
rs11580878	0.2532012	0.4999796	0.2468192
rs11591147	0.9653935	0.0343018	0.0003047
rs117733303	0.9629825	0.0366685	0.0003491
rs12471811	0.7974669	0.1910863	0.0114469
rs1260326	0.1542518	0.4769944	0.3687538
rs12740374	0.6060342	0.3448956	0.0490702
rs12916	0.3593703	0.4802094	0.1604203
rs1367117	0.4370916	0.4480749	0.1148336
rs1601935	0.1186871	0.4516457	0.4296671
rs1883025	0.5579089	0.3780482	0.0640429
rs1883711	0.9385769	0.0604497	0.0009733
rs2125345	0.4990744	0.4147551	0.0861704
rs2237107	0.6333104	0.3249953	0.0416944
rs2244608	0.4686429	0.4318641	0.0994929
rs2618567	0.1161249	0.4492923	0.4345829
rs2738447	0.1661712	0.4829396	0.3508892
rs28601761	0.3342690	0.4877820	0.1779490
rs28807203	0.9046336	0.0929773	0.0023890
rs3127580	0.7081492	0.2667336	0.0251172
rs34042070	0.6625016	0.3028808	0.0346176
rs34707604	0.5518930	0.3820040	0.0661030

SNP	P(Z = 2)	P(Z = 1)	P(Z = 0)
rs3918226	0.8434773	0.1498658	0.0066569
rs4299376	0.1044835	0.4375111	0.4580055
rs4470903	0.6122421	0.3404338	0.0473241
rs456598	0.7353800	0.2443260	0.0202940
rs4704727	0.1153479	0.4485623	0.4360899
rs472495	0.1219232	0.4545036	0.4235732
rs56299331	0.6368870	0.3223300	0.0407830
rs57180587	0.7289642	0.2496596	0.0213762
rs58542926	0.8541959	0.1400626	0.0057415
rs58691354	0.7129641	0.2628159	0.0242201
rs59950280	0.4469685	0.4431771	0.1098545
rs6090040	0.2300488	0.4991705	0.2707808
rs622871	0.0988228	0.4310763	0.4701008
rs635634	0.6627002	0.3027276	0.0345722
rs6458349	0.0768498	0.4007364	0.5224138
rs6511720	0.7764852	0.2093975	0.0141172
rs7012637	0.2755284	0.4987592	0.2257124
rs7213086	0.2001050	0.4944520	0.3054430
rs73534263	0.7971401	0.1913739	0.0114861
rs7412	0.8445834	0.1488576	0.0065590
rs74617384	0.8447171	0.1487357	0.0065473
rs7534572	0.1255675	0.4575751	0.4168575
rs7707394	0.4169078	0.4575523	0.1255398
rs77542162	0.9546715	0.0448029	0.0005257
rs799157	0.0018869	0.0831041	0.9150089
rs9376091	0.5451282	0.3863995	0.0684722
rs964184	0.0174433	0.2292594	0.7532973

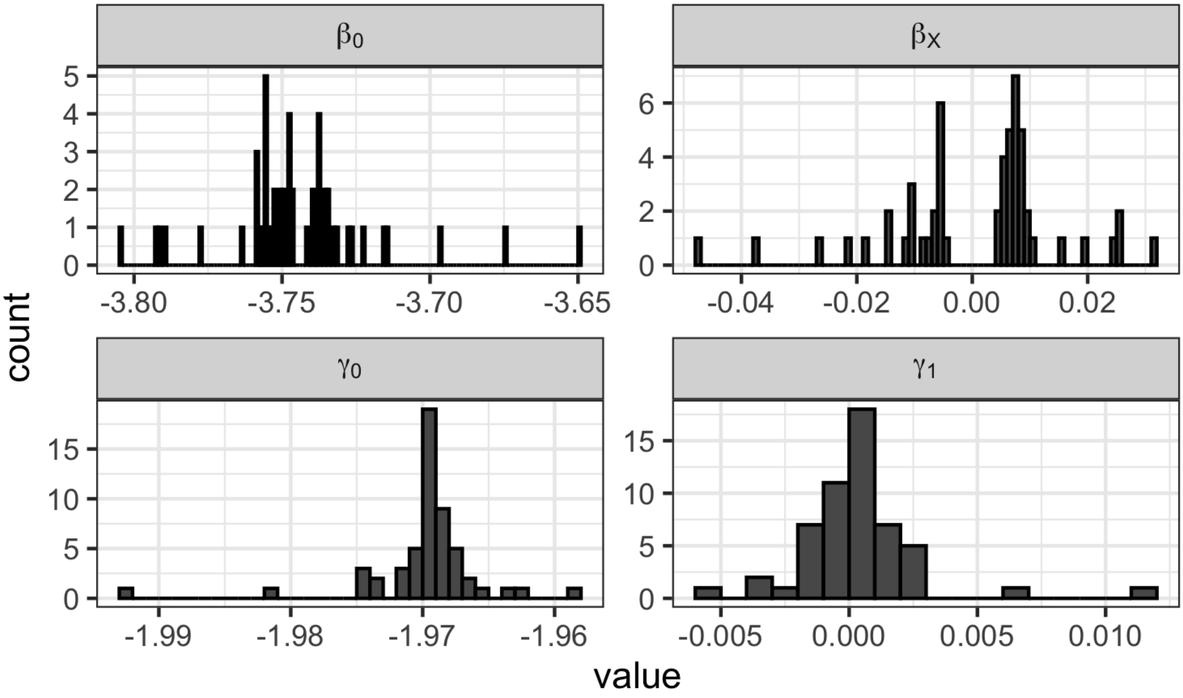

FIGURE K22 Histograms of the coefficients from GWAS results of logistic regression of the SNPs on high cholesterol and heart attack, respectively. Intercepts (β_0 and γ_0) are inferred, while slopes (β_X and γ_1) are as reported.

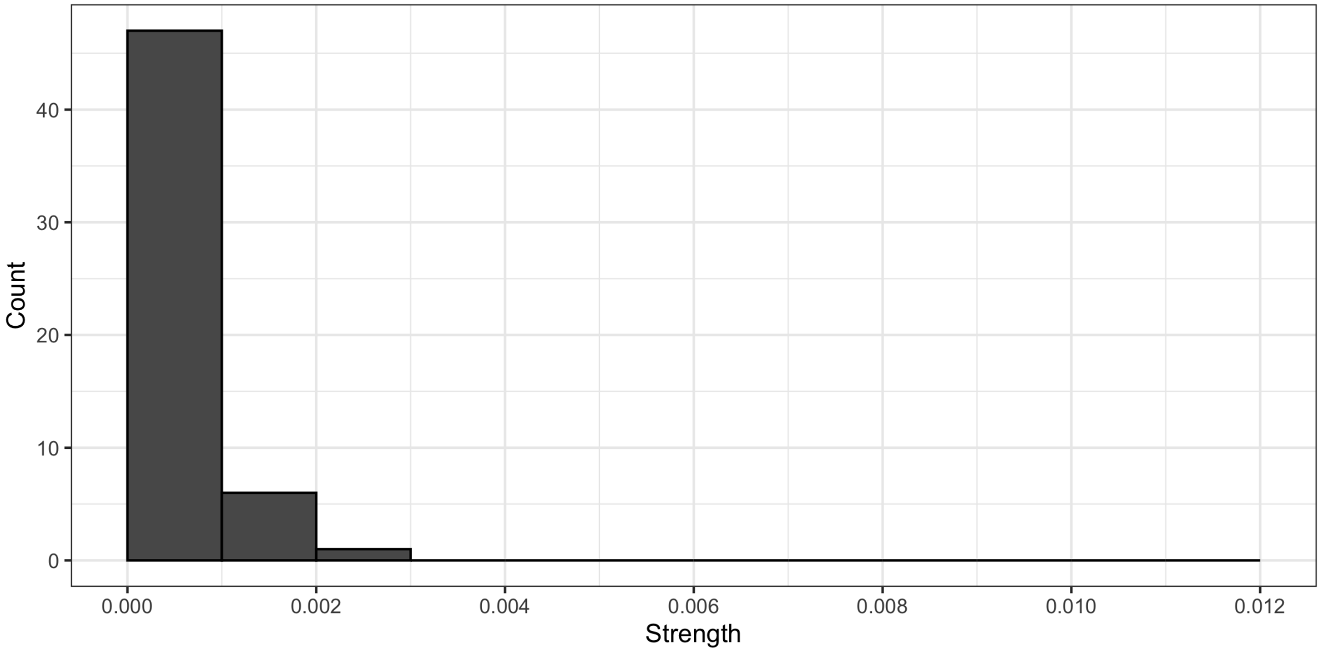

FIGURE K23 Histogram of strengths of IVs on the exposure. Here, SNPs are IVs, and high cholesterol is the exposure. We see that all IVs are very weak, with the largest value below 0.003.

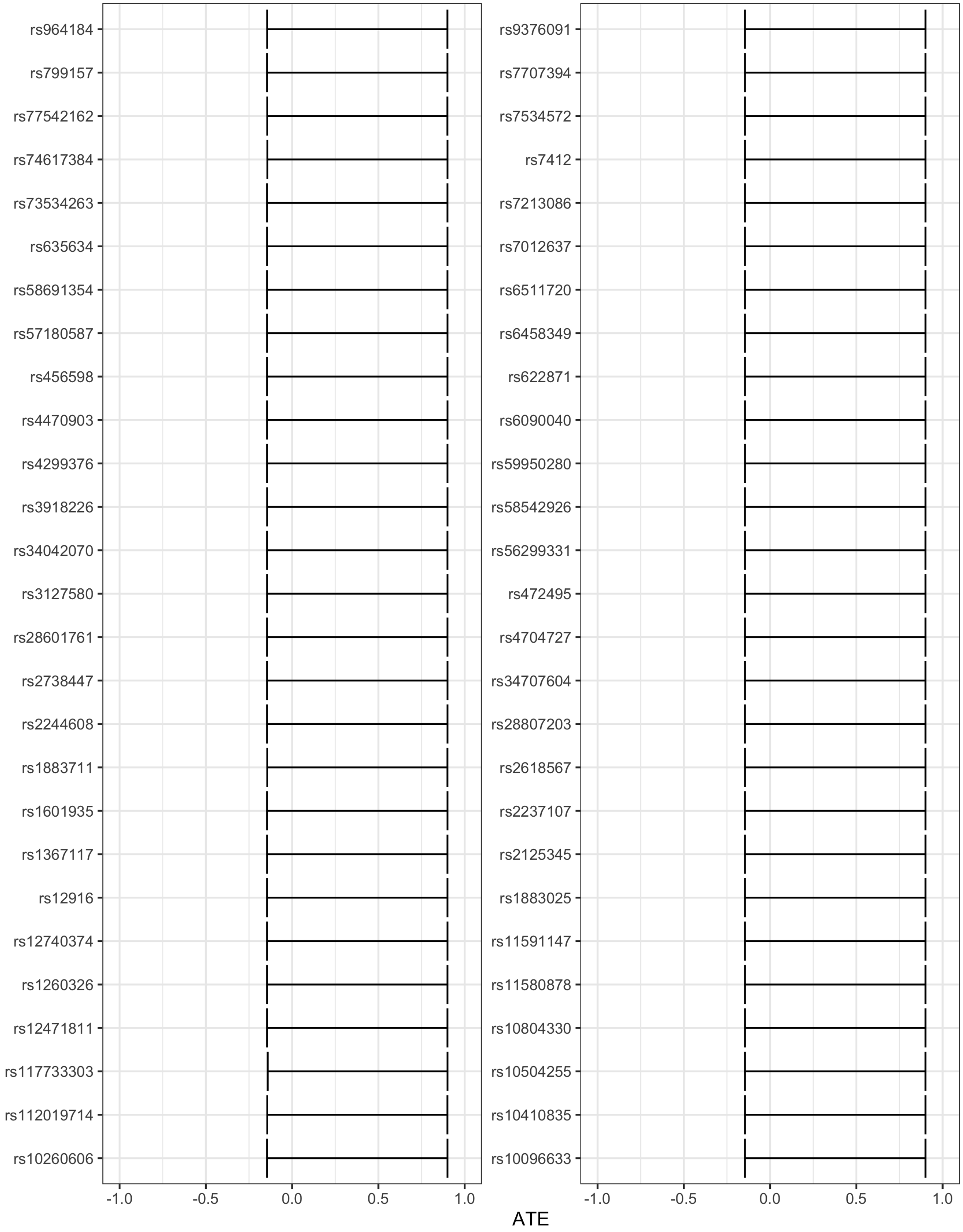

FIGURE K24 Nonparametric two-sample IV bounds on the average treatment effect of high cholesterol on the incidence of heart attack.

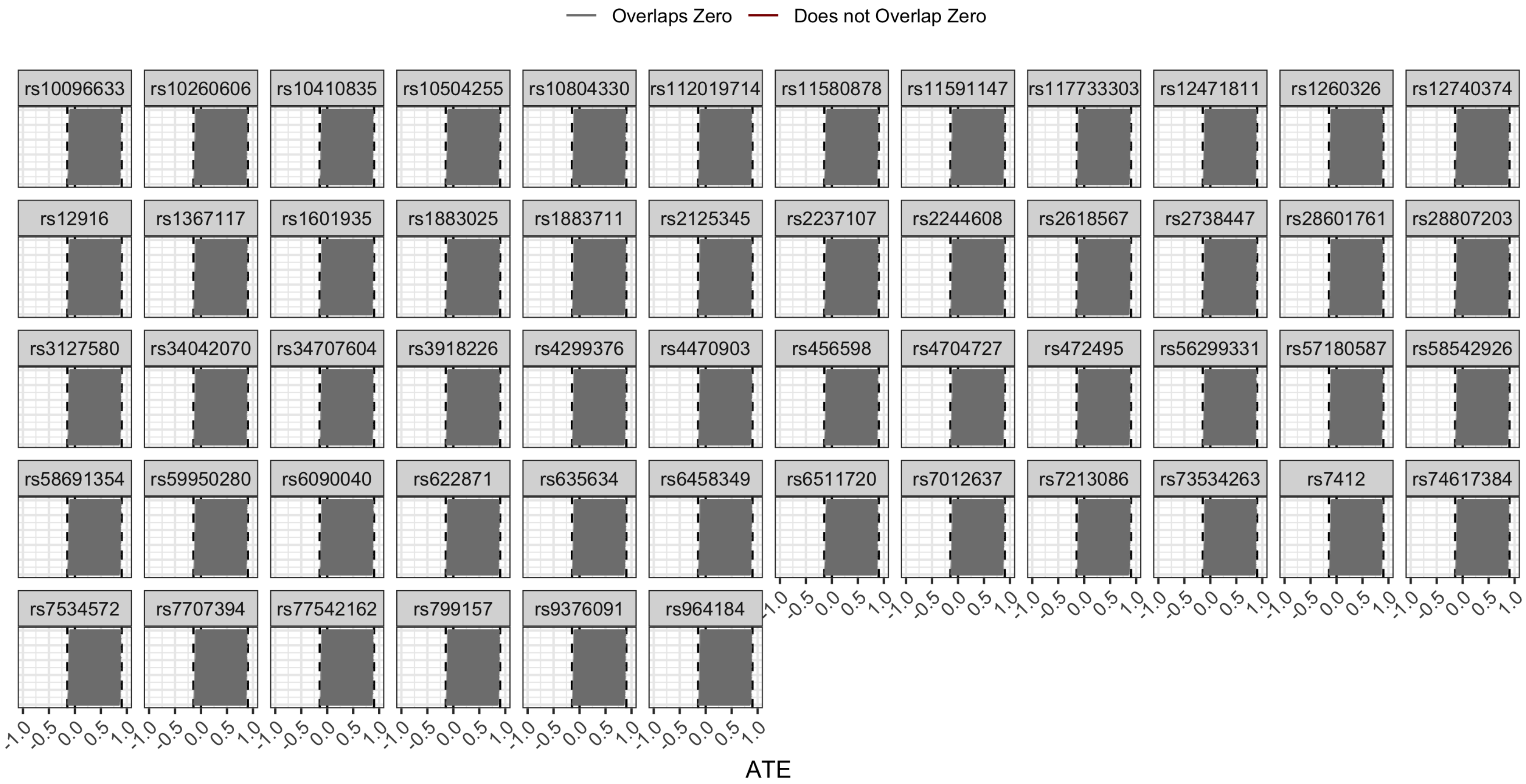

FIGURE K25 500 sets of bounds of the average treatment effect of high cholesterol on heart attack for each of the 54 SNPs. Each bound is based on a set of values for the trivariate distribution randomly sampled. Bounds are color coded to show if they overlap 0 (grey) or do not (red). All bounds overlap 0.

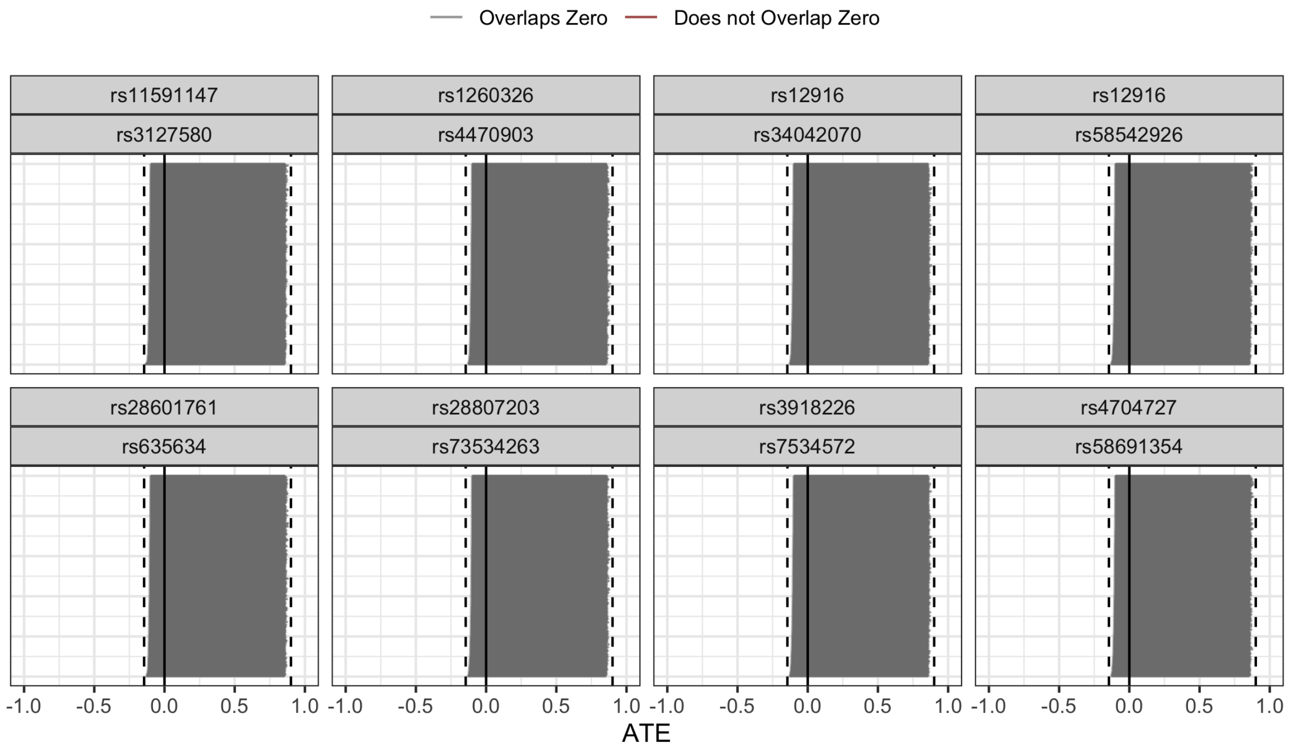

FIGURE K26 Intersection bounds of the average treatment effect of high cholesterol on heart attack based on randomly sampled trivariate distributions from pairs of SNPs. These 8 pairs were randomly chosen from all possible pairs.

References

1. Davey Smith G, Ebrahim S. ‘Mendelian Randomization’: Can Genetic Epidemiology Contribute to Understanding Environmental Determinants of Disease?. *International Journal of Epidemiology* 2003; 32(1): 1–22. doi: 10.1093/ije/dyg070
2. Lawlor DA, Harbord RM, Sterne JAC, Timpson N, Davey Smith G. Mendelian Randomization: Using Genes as Instruments for Making Causal Inferences in Epidemiology. *Statistics in Medicine* 2008; 27(8): 1133–1163. doi: 10.1002/sim.3034
3. Burgess S, Thompson SG. *Mendelian Randomization: Methods for Using Genetic Variants in Causal Estimation*. Boca Raton: Chapman and Hall/CRC. 1st edition ed. 2015.
4. Burgess S, Butterworth A, Thompson SG. Mendelian Randomization Analysis with Multiple Genetic Variants Using Summarized Data. *Genetic Epidemiology* 2013; 37(7): 658–665. doi: 10.1002/gepi.21758
5. Burgess S, Scott RA, Timpson NJ, Davey Smith G, Thompson SG, EPIC- InterAct Consortium . Using Published Data in Mendelian Randomization: A Blueprint for Efficient Identification of Causal Risk Factors. *European Journal of Epidemiology* 2015; 30(7): 543–552. doi: 10.1007/s10654-015-0011-z
6. Davies NM, Holmes MV, Smith GD. Reading Mendelian Randomisation Studies: A Guide, Glossary, and Checklist for Clinicians. *BMJ* 2018; 362. doi: 10.1136/bmj.k601
7. Bowden J, Del Greco M F, Minelli C, Davey Smith G, Sheehan NA, Thompson JR. Assessing the Suitability of Summary Data for Two-Sample Mendelian Randomization Analyses Using MR-Egger Regression: The Role of the I2 Statistic. *International Journal of Epidemiology* 2016; 45(6): 1961–1974. doi: 10.1093/ije/dyw220
8. Bowden J, Davey Smith G, Haycock PC, Burgess S. Consistent Estimation in Mendelian Randomization with Some Invalid Instruments Using a Weighted Median Estimator. *Genetic Epidemiology* 2016; 40(4): 304–314. doi: 10.1002/gepi.21965
9. Verbanck M, Chen CY, Neale B, Do R. Detection of Widespread Horizontal Pleiotropy in Causal Relationships Inferred from Mendelian Randomization between Complex Traits and Diseases. *Nature Genetics* 2018; 50(5): 693–698. doi: 10.1038/s41588-018-0099-7
10. Zhao Q, Wang J, Hemani G, Bowden J, Small DS. Statistical Inference in Two-Sample Summary-Data Mendelian Randomization Using Robust Adjusted Profile Score. *Annals of Statistics* 2020; 48(3): 1742–1769. doi: 10.1214/19-AOS1866
11. Burgess S, Small DS, Thompson SG. A Review of Instrumental Variable Estimators for Mendelian Randomization. *Statistical Methods in Medical Research* 2017; 26(5): 2333–2355. doi: 10.1177/0962280215597579
12. Slob EAW, Burgess S. A Comparison of Robust Mendelian Randomization Methods Using Summary Data. *Genetic Epidemiology* 2020; 44(4): 313–329. doi: 10.1002/gepi.22295
13. Balke A, Pearl J. Bounds on Treatment Effects from Studies with Imperfect Compliance. *Journal of the American Statistical Association* 1997; 92(439): 1171–1176. doi: 10.1080/01621459.1997.10474074
14. Cheng J, Small DS. Bounds on Causal Effects in Three-Arm Trials with Non-Compliance. *Journal of the Royal Statistical Society. Series B (Statistical Methodology)* 2006; 68(5): 815–836.
15. Manski CF. Nonparametric Bounds on Treatment Effects. *The American Economic Review* 1990; 80(2): 319–323.
16. Richardson TS, Robins JM. ACE Bounds; SEMs with Equilibrium Conditions. *Statistical Science* 2014; 29(3): 363–366. doi: 10.1214/14-STS485
17. Robins JM. The Analysis of Randomized and Nonrandomized AIDS Treatment Trials Using A New Approach to Causal Inference in Longitudinal Studies. *Health Service Research Methodology: A Focus On AIDS* 1989: 113–159.
18. Ramsahai RR. Causal Bounds and Observable Constraints for Non-Deterministic Models. *J. Mach. Learn. Res.* 2012; 13: 829–848.

19. Swanson SA, Hernán MA, Miller M, Robins JM, Richardson TS. Partial Identification of the Average Treatment Effect Using Instrumental Variables: Review of Methods for Binary Instruments, Treatments, and Outcomes. *Journal of the American Statistical Association* 2018; 113(522): 933–947. doi: 10.1080/01621459.2018.1434530

20. Didelez V, Sheehan N. Mendelian Randomization as an Instrumental Variable Approach to Causal Inference. *Statistical Methods in Medical Research* 2007; 16(4): 309–330. doi: 10.1177/0962280206077743

21. Swanson SA. Commentary: Can We See the Forest for the IVs? Mendelian Randomization Studies with Multiple Genetic Variants. *Epidemiology* 2017; 28(1): 43–46. doi: 10.1097/EDE.0000000000000558

22. Burgess S, Labrecque JA. Mendelian Randomization with a Binary Exposure Variable: Interpretation and Presentation of Causal Estimates. *European Journal of Epidemiology* 2018; 33(10): 947–952. doi: 10.1007/s10654-018-0424-6

23. Rubin DB. Estimating Causal Effects of Treatments in Randomized and Nonrandomized Studies.. *Journal of Educational Psychology* 1974; 66(5): 688–701. doi: 10.1037/h0037350

24. Splawa-Neyman J. On the Application of Probability Theory to Agricultural Experiments. Essay on Principles. Section 9.. *Statistical Science* 1923; 5(4): 465–472. Translated in 1990.

25. Cox DR. *Planning of Experiments*. Planning of ExperimentsOxford, England: Wiley . 1958.

26. Rubin DB. Randomization Analysis of Experimental Data: The Fisher Randomization Test Comment. *Journal of the American Statistical Association* 1980; 75(371): 591–593. doi: 10.2307/2287653

27. Wang L, Tchetgen Tchetgen E. Bounded, Efficient and Multiply Robust Estimation of Average Treatment Effects Using Instrumental Variables. *arXiv:1611.09925 [stat]* 2018.

28. Stock JH, Wright JH, Yogo M. A Survey of Weak Instruments and Weak Identification in Generalized Method of Moments. *Journal of Business & Economic Statistics* 2002; 20(4): 518–529. doi: 10.1198/073500102288618658

29. Burgess S. Sample Size and Power Calculations in Mendelian Randomization with a Single Instrumental Variable and a Binary Outcome. *International Journal of Epidemiology* 2014; 43(3): 922–929. doi: 10.1093/ije/dyu005

30. Burgess S, Thompson SG. Improving Bias and Coverage in Instrumental Variable Analysis with Weak Instruments for Continuous and Binary Outcomes. *Statistics in Medicine* 2012; 31(15): 1582–1600. doi: 10.1002/sim.4498

31. Millard LAC, Munafò MR, Tilling K, Wootton RE, Smith GD. MR-pheWAS with Stratification and Interaction: Searching for the Causal Effects of Smoking Heaviness Identified an Effect on Facial Aging. *PLOS Genetics* 2019; 15(10): e1008353. doi: 10.1371/journal.pgen.1008353

32. Manolio TA. Genomewide Association Studies and Assessment of the Risk of Disease. ; 363(2): 166–176. doi: 10.1056/NEJMra0905980

33. Diemer EW, Labrecque J, Tiemeier H, Swanson SA. Application of the Instrumental Inequalities to a Mendelian Randomization Study With Multiple Proposed Instruments. *Epidemiology* 2020; 31(1): 65. doi: 10.1097/EDE.0000000000001126

34. Cholesterol Treatment Trialists' (CTT) Collaborators . The Effects of Lowering LDL Cholesterol with Statin Therapy in People at Low Risk of Vascular Disease: Meta-Analysis of Individual Data from 27 Randomised Trials. *The Lancet* 2012; 380(9841): 581–590. doi: 10.1016/S0140-6736(12)60367-5

35. Cornfield J, Haenszel W, Hammond EC, Lilienfeld AM, Shimkin MB, Wynder EL. Smoking and Lung Cancer: Recent Evidence and a Discussion of Some Questions. *JNCI: Journal of the National Cancer Institute* 1959; 22(1): 173–203. doi: 10.1093/jnci/22.1.173

36. Schmitt J, Seidler A, Diepgen TL, Bauer A. Occupational Ultraviolet Light Exposure Increases the Risk for the Development of Cutaneous Squamous Cell Carcinoma: A Systematic Review and Meta-Analysis. *Br J Dermatol* 2011; 164(2): 291–307. doi: 10.1111/j.1365-2133.2010.10118.x

37. Loh PR, Bhatia G, Gusev A, et al. Contrasting Genetic Architectures of Schizophrenia and Other Complex Diseases Using Fast Variance-Components Analysis. *Nature Genetics* 2015; 47(12): 1385–1392. doi: 10.1038/ng.3431

38. Shi H, Kichaev G, Pasaniuc B. Contrasting the Genetic Architecture of 30 Complex Traits from Summary Association Data. *The American Journal of Human Genetics* 2016; 99(1): 139–153. doi: 10.1016/j.ajhg.2016.05.013

39. Nj T, Cmt G, N S, Dj L, Jb R. Genetic Architecture: The Shape of the Genetic Contribution to Human Traits and Disease.. *Nature reviews. Genetics* 2017; 19(2): 110–124. doi: 10.1038/nrg.2017.101

40. Yang J, Benyamin B, McEvoy BP, et al. Common SNPs Explain a Large Proportion of the Heritability for Human Height. *Nature Genetics* 2010; 42(7): 565–569. doi: 10.1038/ng.608

41. on Smoking SGAC, ealth, United States. . *Smoking and Health: Report of the Advisory Committee to the Surgeon General of the Public Health Service*. Public Health Service PublicationU.S. Department of Health, Education, and Welfare, Public Health Service . 1964.

42. Holmes MV, Asselbergs FW, Palmer TM, et al. Mendelian Randomization of Blood Lipids for Coronary Heart Disease. *European Heart Journal* 2015; 36(9): 539–550. doi: 10.1093/eurheartj/eht571

43. Richardson TG, Sanderson E, Palmer TM, et al. Evaluating the Relationship between Circulating Lipoprotein Lipids and Apolipoproteins with Risk of Coronary Heart Disease: A Multivariable Mendelian Randomisation Analysis. *PLOS Medicine* 2020; 17(3): e1003062. doi: 10.1371/journal.pmed.1003062

44. Cholesterol Treatment Trialists' (CTT) Collaborators . Efficacy and Safety of Cholesterol-Lowering Treatment: Prospective Meta-Analysis of Data from 90 056 Participants in 14 Randomised Trials of Statins. *The Lancet* 2005; 366(9493): 1267–1278. doi: 10.1016/S0140-6736(05)67394-1

45. Hemani G, Zheng J, Elsworth B, et al. The MR-Base platform supports systematic causal inference across the human phenome. *eLife* 2018; 7: e34408. doi: 10.7554/eLife.34408

46. Stock J, Yogo M. *Testing for Weak Instruments in Linear IV Regression*. New York: Cambridge University Press . 2005.
